# Vitamin B_12_ and Micellar Solution Enable Regioselective Ring Opening of Epoxides and Aziridines with Electrophilic Olefins

**DOI:** 10.1021/acs.orglett.5c01376

**Published:** 2025-05-23

**Authors:** Kitti Franciska Szabó, Tomasz Wdowik, Aleksandra Krzeszewska, Krzysztof Mazurek, Martin P. Andersson, Dorota Gryko

**Affiliations:** † Institute of Organic Chemistry, 154690 Polish Academy of Sciences, Kasprzaka 44/52, 01-224 Warsaw, Poland; ‡ Center for Integrative Petroleum Research, 48080 King Fahd University of Petroleum and Minerals, Dhahran 31261, Kingdom of Saudi Arabia

## Abstract

Vitamin B_12_, a water-soluble cobalt complex, is inherently predisposed to catalyze reactions under aqueous conditions. Despite its potential, adopting this strategy for transformations of hydrophobic reagents has been challenging, because of their low aqueous solubility. Here, we demonstrate that vitamin B_12_ promotes the reaction of epoxides and aziridines with electrophilic olefins in a micellar system. The desired products are obtained efficiently in a fully regioselective manner. This green catalytic approach further advances the use of vitamin B_12_ in sustainable catalysis providing a valuable method to synthesize important intermediates.

## Graphical abstract

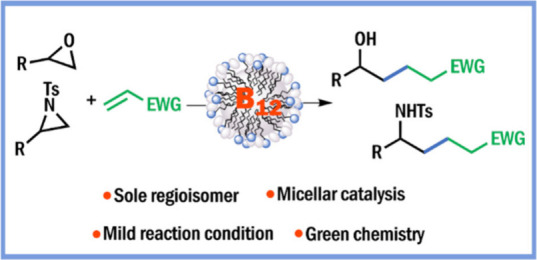

Three-membered heterocycles, epoxides, and aziridines, are versatile synthetic intermediates in organic synthesis, with broad applications ranging from polymer chemistry to chemical biology.^1–7^ Along this line, the ring-opening reactions with nucleophiles that often enable further chemical transformations are highly prized. Classical methods for epoxide and aziridine ring-opening mostly rely on acid or base catalysis,^8–10^ involve transition metal complexes^11,12^ or organocatalysts.^13^ These approaches, however, often suffer from harsh reaction conditions, poor selectivity or low yields. Thus, greener, selective methods for their transformations are highly desired.

In recent years, sustainable ring-opening protocols, including photochemical transformations, have attracted substantial attention.^5^ For example, in 2020, Doyle et al. reported photocatalyzed cross-electrophile coupling of epoxides and aziridines with aryl iodides in the presence of 4-CzIPN/Ni.^14,15^ Notably, both aliphatic and aromatic derivatives yielded phenylamine derivatives, but only for alkyl substituted aziridines, these reactions were fully regioselective. Side reactions such as homocoupling of aryl iodides and epoxide rearrangements represented a challenge and required careful ligand selection. Subsequent advances led to asymmetric variants allowing them to obtain linear products with moderate to high enantioselectivity.^16^ Further improvements included a photocatalytic aziridine ring-opening reaction employing acetals as alkyl radical sources.^17^ In 2021, our group developed a dual vitamin B_12_/Ni catalytic system for the regioselective ring-opening of aryl and alkyl epoxides with aryl halides (Scheme 1A).^18^ It is the vitamin that governs the regioselectivity of the ring-opening on the less hindered side of the epoxide. Subsequently, this methodology was extended to include the ring-opening of oxetane derivatives, which required the addition of a Lewis acid.^19^ The West group reported vitamin a B_12_/HAT-catalyzed reduction of epoxides selectively yielding Markovnikov alcohols (Scheme 1A).^20^ However, these photochemical transformations are typically conducted in organic solvents that pose environmental and safety concerns.^21^ Therefore, we sought to use an alternative reaction medium, namely micellar solutions, which not only enhance solubilization but also may improve regioselectivity.^22–25^ In this context, our group has recently demonstrated that micellar solutions are not only compatible with vitamin B_12_-catalyzed radical addition/1,2-aryl migration reactions, but are also essential for achieving high yields of the desired product (Scheme 1B).^26^

**Scheme 1:**
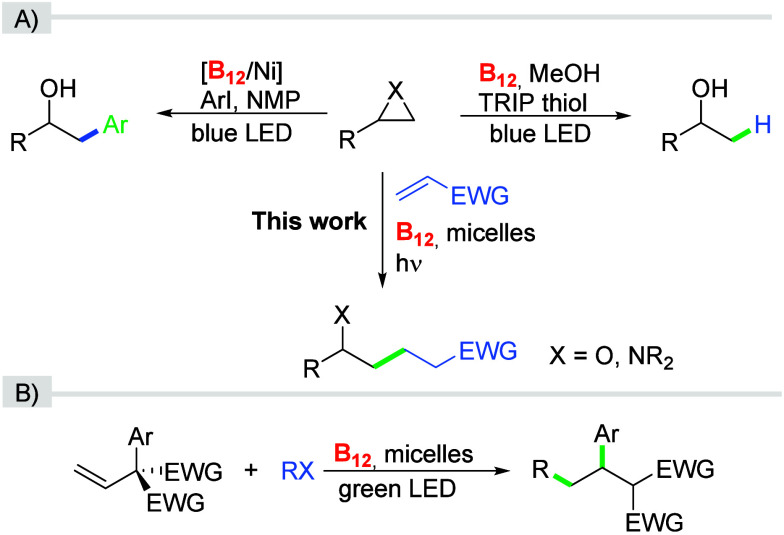
(A) Photochemical Functionalizations of Epoxides and Aziridines; (B) Vitamin B_12_-Catalyzed Radical Addition/1,2-aryl Migration Reaction in Micelles

Recognizing the potential of micellar systems to direct reactivity, we have strived to investigate whether they could facilitate the ring-opening of epoxides and aziridines (Scheme 1A). Given that these strained three-membered heterocycles are fundamental building blocks, exploring their regioselective transformations under micellar conditions aligns well with our goal of developing sustainable methodologies. What is more, the role of micellar solutions in vitamin B_12_-catalyzed reactions remains underexplored.

Vitamin B_12_, in its reduced Co­(I) form, is known to open the epoxide^18,27^ ring from the less hindered side due to its “supernucleophilicity” in organic solvents. Based on our previous studies, for our preliminary studies we selected the ring-opening of 2-(phenoxymethyl)­oxirane (**1**) with acrylonitrile (**2**) as a model reaction. Initially, hydrophobic heptamethyl cobyrinate (HME) as catalyst, Zn/NH_4_Cl as a reducing agent were used in acetonitrile under blue LED irradiation. The reaction afforded desired product **3** in 43% yield (Table 1, entry 1). Replacement of HME with native vitamin B_12_ and organic solvent with micellar solution (dodecyl trimethylammonium chloride, DTAC, as a surfactant) improved the yield up to 59% (entry 2). All tested cationic surfactants performed well, except for 1-hexadecylpyridinium bromide, which afforded only 31% of the desired product (see Supporting Information (SI) for more details). The use of DTAC eliminated also the need for NH_4_Cl, which is typically required for B_12_-catalyzed reactions.^28^ On the contrary, anionic surfactants, such as potassium laurate and sodium lauryl sulfate (SLES), were less efficient in both reactions. Increasing the amount of the surfactant and vitamin B_12_ further improved yields (entries 2 and 3), while any changes in their concentrations decreased the efficacy of the reaction (entry 4). The addition of alcohol cosolvents significantly improved yields (entry 5), with ethanol proving the most effective (see SI). Alcohols are believed to integrate into the micellar interface, increasing its flexibility and enhancing the capacity of the hydrophobic microenvironment within the aqueous solution. This adjustment likely improves the permeability of the interface to organic compounds.^27^ All other reaction parameters, including light source, light power, and substrate ratios, were also optimized (see SI). Under the optimized conditions—native vitamin B_12_, Zn as a reductant, DTAC as a surfactant, and a H_2_O/EtOH (9:1) solvent mixture, irradiated with blue light (446 nm)—the model reaction yielded the desired product in 85% yield (entry 6).

**Table 1:**
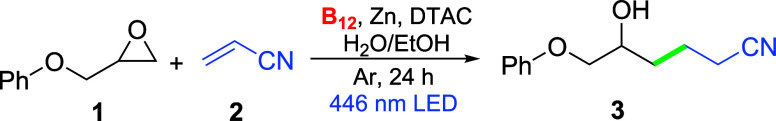
Optimization Studies of the Ring Opening of Epoxide (**1**) with Acrylonitrile (**2**)­^a^

entry	deviation from standard conditions	yield **3** (%)^b^
1	HME, MeCN	43
2	2.5 equiv DTAC	59
3	2.5 mol% B_12_	66
4	0.03 M	70
5	no additives	76
**6**	**none**	**85**
7	no light	n.d.
8	no B_12_	n.d.
9	no Zn	n.d.

a Conditions: epoxide (**1**, 0.2 mmol), acrylonitrile (2, 1.5 equiv), B_12_ (5 mol %), Zn (3 equiv), DTAC (5 equiv), H_2_O/EtOH (9:1, v/v, c = 0.04 M), blue LEDs (446 nm, 3 W), 24 h.

b Yields determined by GC FID analysis, n.d. = not detected.

In parallel, the conditions for the aziridine ring-opening were optimized, building upon the selected parameters for epoxides (detailed optimization data can be found in the SI, with key differences highlighted in Table 2). Using 2-butyl-1-tosylaziridine (**4**) and acrylonitrile (**2**) as model substrates, we found that reducing the amount of DTAC to 3.5 equiv and vitamin B_12_ to 2.5 mol % proved beneficial, enabling the synthesis of protected amine **5** in 57% (entries 1–2). Switching the light source from blue (446 nm) to green (525 nm) further improved the yield to 61% (entry 3).

**Table 2:**
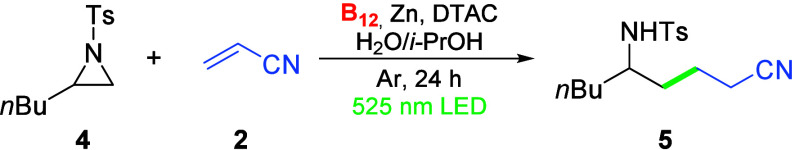
Optimization Studies of the Ring Opening of Aziridine (**4**) with Acrylonitrile (**2**)­^a^

entry	deviation from standard conditions	yield **5** (%)^b^
1	3.5 equiv DTAC	57
2	2.5 mol% B_12_	57
3	green LED (40 W)	61
**4**	***i-*PrOH**	**83, 80^c^**
5	no DTAC	29

a Conditions: aziridine (**4**, 0.2 mmol), acrylonitrile (2, 1.5 equiv), B_12_ (2.5 mol %), Zn (3 equiv), DTAC (3.5 equiv), H_2_O/*i*PrOH (9:1, v/v, c = 0.04 M), green LEDs (525 nm, 40 W), 24 h.

b Yields determined by GC FID analysis.

c Isolated yield.

Finally, the replacement of EtOH with *i*-PrOH as a cosolvent had the most significant impact (83%, entry 4). In this case, the model reaction proceeded even in the absence of DTAC, but given that both starting materials are liquids, it is plausible that this reaction partially occurs as an ‘on-water’ process (entry 5).

Vitamin B_12_ as a hydrophilic molecule should be found in the aqueous phase, but our theoretical studies indicate that in the Co­(I)-form it is located at the micellar interface. Other and our studies clearly indicate that reactions in micellar solutions are strongly affected by the philicity of starting materials, in contrast to those performed in organic solvents.^26,29^ For that reason, the alkyl chain length and functional groups that significantly influence the location of molecules within micellar solutions, affect the efficacy of the reaction. Consequently, we tested the behavior of structurally diverse epoxides but not tetra and three substituted, as for it has already been documented that these are not suitable substrates for vitamin B_12_-catalyzed reactions (Scheme 2A).^18^ These reactions yielded Markovnikov alcohol products in moderate to high yields (33–85%) in a fully regioselective manner. 2-(Phenoxymethyl)oxirane with (vinylsulfonyl)­benzene as the acceptor afforded product **6** but in lower yields, while other ester-derived acceptors remained unreactive toward the epoxide (see SI for details). These starting materials are less polar than acrylonitrile, and presumably they are buried deeper inside the micelle and become less accessible to the radical, presumably formed within the Stern layer. In such small molecules as Michael acceptors, philicity and, as a result, a location in the micelle is governed by an EWG group. For phenyloxirane, additional optimization was required, reducing the amount of surfactant and increasing acrylonitrile to 5 equiv was required to achieve a yield of 48% (**7**). A fluorine substituent in the *para* position of the aromatic ring (**8**) also afforded the desired product in 53% yield as a sole regioisomer. These both oxiranes are more hydrophobic substrates, therefore their contact with catalytically active vitamin B_12_ located at the interface is less favorable. Interestingly, the yield with *n*-butyl epoxide increased significantly to 73% (**9**), possibly because its shorter chain allows for greater mobility and facilitates proper orientation. The longer hydrophobic decyl derivative, provided nitrile **10** in 40% yield, probably due to the not optimal alignment of the starting materials within the micelle in contrast to reactions with arylbromides. Naphthalene and phenylethyl carbamate derivatives gave desired products **11**, **12** in lower yields of 41% and 40%, respectively. The benzylcarbamate and phenylsulfonyl derivatives were well tolerated as (**13** and **14** formed in good yields of 53% and 63%, respectively). Polar substituents present in these substrates have a stronger affinity for the interface, and as a result, the reaction occurs efficiently.

**Scheme 2:**
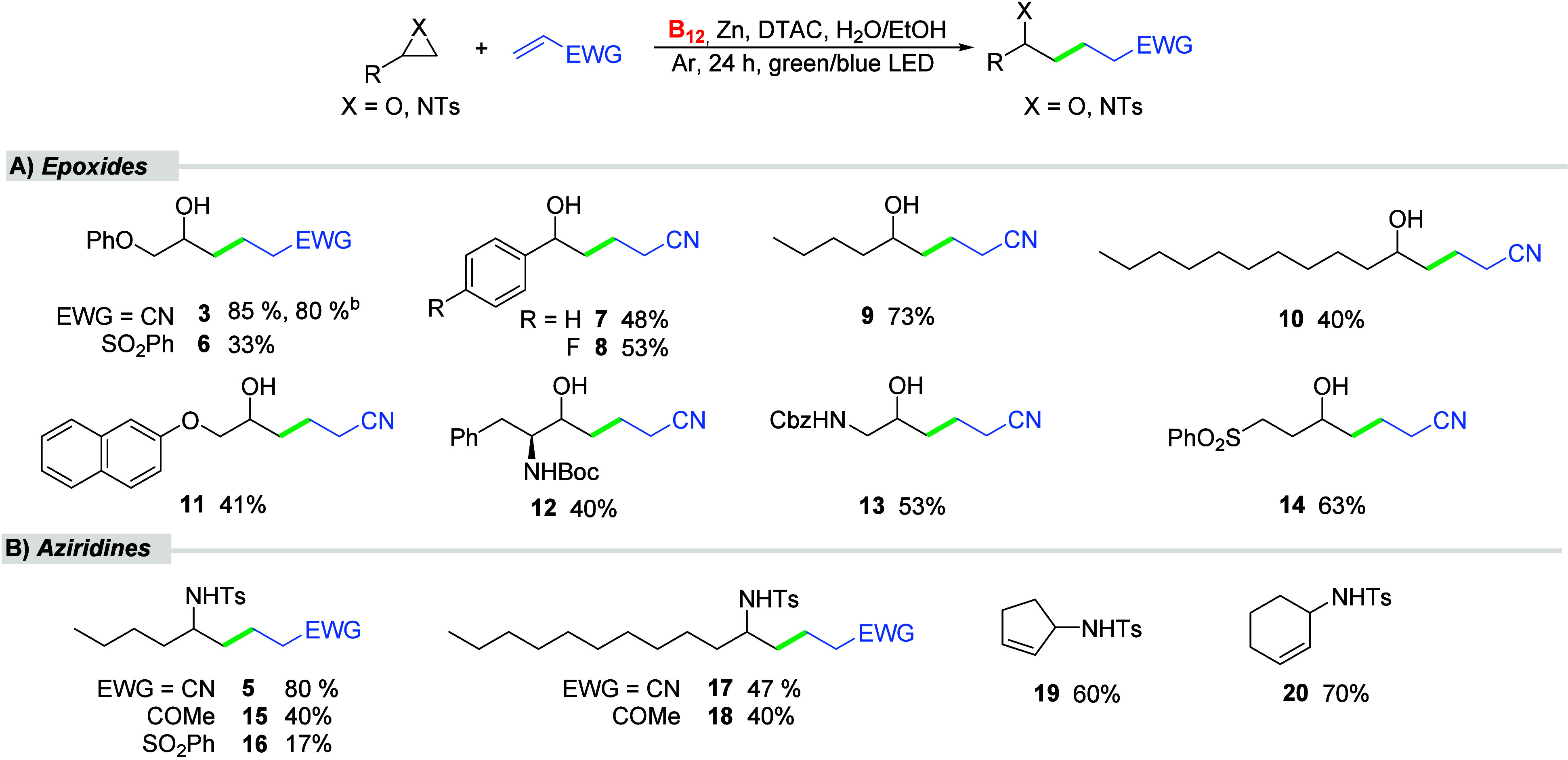
Scope of the Giese Addition of Epoxides and Aziridines to Electrophilic Alkenes^a^

a Reaction conditions: A) epoxide (0.2 mmol), alkene (**2**, 1.5 equiv), B_12_ (5 mol %), Zn (3 equiv), DTAC (5 equiv), H_2_O/EtOH (9:1, v/v) *c* = 0.04 M, blue LEDs (446 nm, 3 W), time 24 h. B) aziridine (0.2 mmol), alkene (1.5 equiv), B_12_ (2.5 mol %), Zn (3 equiv), DTAC (3.5 equiv), H_2_O/*i*PrOH (9:1, v/v) *c* = 0.04 M, green LEDs (525 nm, 40 W), 24 h.
b Reaction performed on a 1 mmol scale.

Furthermore, to prove the synthetic utility of the developed method, the model reaction was performed on a 1 mmol scale. The desired product was isolated in 80% yield.

Similar observations were made for the reaction of aziridines (Scheme 2B). The reaction of 2-butyl-1-tosylaziridine with a ketone-derived Michael acceptor afforded the desired product **15** in 40% yield, while for vinylsulfonylbenzene as the acceptor the yield substantially diminished (**16**). A broad range of other acrylates proved unreactive under these conditions (see SI). Therefore, the functional groups of acrylates have a substantial impact on their organization within the micelles. Increasing the alkyl chain length of the aziridines (**17–18**) gave comparable results regardless of the use of different Michael acceptors.

Further tests with various aziridines (see SI) revealed additional limitations of our method. Intriguingly, under the developed conditions, aryl aziridines and *N-*methyl- or *N-*dodecyl-substituted aziridines remained unreactive, with no conversion of starting materials nor the formation of ring-opened products observed. We hypothesize that the reaction occurs within the Stern layer, where the Co-catalyst’s active form is located.^26^ This requires the nitrogen atom to be oriented toward the interface, and hydrophobic protecting groups interfere with this necessary orientation within the micellar solution. Intriguingly, the azabicyclo derivatives, azabicyclohexane (**19**) and azabicycloheptane (**20**) led to allylamines of 60% and 70% in yields, respectively. Scheffold and Zhang reported the plausible mechanism for this process.^30^ The aziridine ring is opened via the S_N_2 mechanism to generate a Co­(III)­cycloalkyl derivative. Subsequent elimination occurs to yield the allylamine derivative.

Based on the mechanistic insights gained and prior reports, we propose the following reaction mechanism analogous to that observed in organic solvents (Figure 1A).^31–33^ In the presence of Zn/DTAC, vitamin B_12_ is reduced to “supernucleophilic” Co­(I) species. The efficient reduction of Co­(III) to Co­(I) is indicated by a color change from pink to deep brown (see SI). This Co­(I) species then attacks the less hindered side of the epoxide or aziridine ring, resulting in ring opening in a Markovnikov fashion and forming a Co­(III)-alkyl anion intermediate **A**. Subsequently, protonation of intermediate **A** leads to the formation of alkylcobalamin intermediate **B**. Upon light irradiation, the homolytic cleavage of **B** generates a carbon-centered alkyl radical **C** and a Co­(II) complex. The alkyl radical **C** is then captured by electron-deficient acrylonitrile to form radical intermediate **D**, which is further protonated to yield the final product.

**Figure 1:**
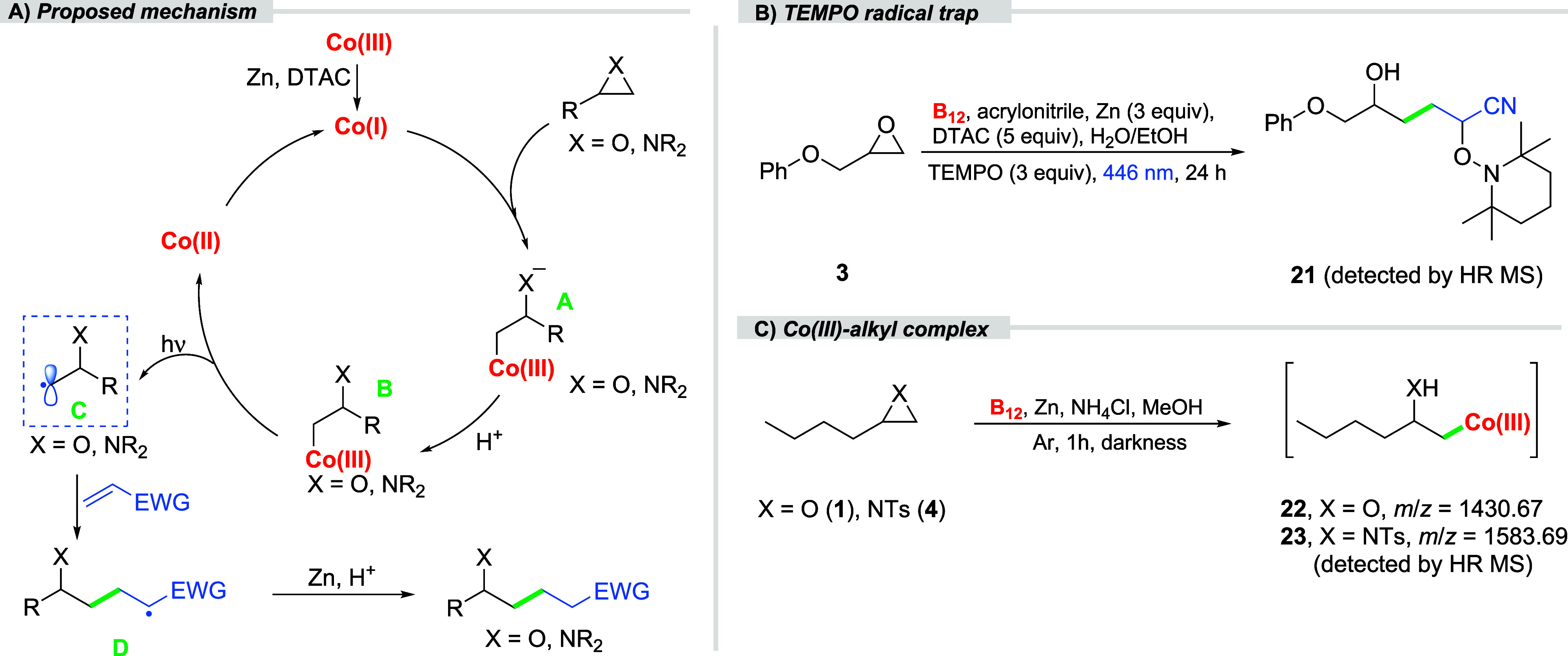
Mechanistic investigations: A) proposed reaction mechanism, B) detection of the product/TEMPO adduct, C) key mechanistic intermediates detected.

Indeed, control experiments confirmed that light, vitamin B_12_, and Zn are essential for the reaction to proceed (Table 1, entries 7–9; see SI). To further support the mechanistic pathway, a radical trap experiment with TEMPO was performed. It allowed to detect adduct **21** by HRMS analysis. (Figure 1B; see SI). This strongly suggests that a radical mechanism is at play and that radical **D** is an intermediate in the catalytic cycle. In the absence of acrylonitrile and light, ESI-MS analysis detected alkylcobalamin intermediates **22–23**, further proving the proposed mechanism (Figure 1C).

In summary, we have developed a regioselective epoxide- and aziridine ring-opening reaction catalyzed by vitamin B_12_ in the micellar system. This method successfully converted alkyl-/aryl epoxides and alkyl aziridines in the presence of acrylate derivatives to the desired products with moderate to good yields, forming a single regioisomer. Mechanistic studies support our proposed reaction pathway, which involves the initial ring-opening of strained molecules by the Co­(I) species, followed by homolytic cleavage of the Co­(III)–C bond to produce alkyl radicals.

Our work advances the use of vitamin B_12_ in catalysis, offering a sustainable strategy for the synthesis of important molecular structures and emphasizes the need for further insights into micellar catalysis which is now ongoing in our laboratory.

## Supplementary Material



## Data Availability

The data underlying this study are available in the published article and the Supporting Information.

## Notes

Full description of optimization and mechanistic studies, general procedures, compound characterization (NMR, HRMS), and NMR spectra (PDF)
