# Supplementary material for: Vitamin B12 and Micellar Solution Enable Regioselective Ring Opening of Epoxides and Aziridines with Electrophilic Olefins
Source: Org Lett. 2025 May 23;27(22):5642–6. doi: 10.1021/acs.orglett.5c01376 (PMC12150321; doi:10.1021/acs.orglett.5c01376)

Supporting Information

Vitamin B_{12} and Micellar Solution Enable Regioselective Ring Opening of Epoxides and Aziridines with Electrophilic Olefins

Kitti Franciska Szabó, Tomasz Wdowik, Aleksandra Krzeszewska, Krzysztof Mazurek, Martin P. Andersson, and Dorota Gryko*

Institute of Organic Chemistry Polish Academy of Science
Kasprzaka 44/52, 01-224 Warsaw, Poland

Center for Integrative Petroleum Research, King Fahd University of Petroleum and Minerals, Dhahran 31261, Kingdom of Saudi Arabia

e-mail: dorota.gryko@icho.edu.pl

Table of Contents

1. General information **4**

2. Setup for photoreactions **6**

3. Surfactants and their abbreviations **7**

4. Background experiments of alkyl epoxides with olefins **8**

5. Optimization of reactions parameters of alkyl epoxides with olefins **9**

5.1. The influence of light 9

5.2. Screening of surfactants 10

A) HME-catalyzed reactions 10

B) B_{12}-catalyzed reactions 10

C) The amount of the surfactant 10

5.3. B_{12} – Impact of the catalyst loading 11

5.4. Optimization of the substrates' ratio 11

5.5. The influence of the amount of Zn 11

5.6. Concentration of epoxide (1) 12

5.7. Screening of additives 12

6. Optimization of reactions parameters of aryl epoxides with olefins **13**

6.1. The influence of light 13

6.2. Screening of surfactant 13

6.3. The influence of the amount of Zn 14

6.4. Screening of additives 14

6.5. The amount of EtOH 14

6.6. B_{12} – Impact of the catalyst loading 15

6.7. Optimization of the substrates' ratio 15

7. Background experiments for alkyl aziridine reaction with olefin **16**

8. Optimization of reactions parameters of alkyl aziridines with olefins **17**

8.2. The influence of light 17

8.3. Screening of surfactants 18

B) The amount of the surfactant 18

8.4. B_{12} – Impact of the catalyst loading 19

8.5. Optimization of the substrates' ratio 19

8.6. The influence of the amount of Zn 19

8.7. Concentration of aziridine 20

8.8. Screening of additives 20

9. Mechanistic consideration **21**
9.1. Proposed mechanism 21
9.2. Kinetic studies of the model alkyl epoxide 22
9.3. Co(III)-alkyl complex formation 23
9.4. Experiment with a radical trap 26
10. Preparation of starting materials (S1-S12) and characterization of new compounds **28**
10.1. Synthesis of starting materials 28
10.2. Scope limitation: unsuccessful starting materials 29
11. General Procedures **30**
11.2 Procedure for the 1 mmol scale synthesis of 5-hydroxy-6-phenoxyhexanenitrile (3) 31
12. Scope and characterization of new compounds **32**
12.1. Epoxides 32
12.2. Aziridines 36
13. NMR Spectra **40**
5-hydroxy-6-phenoxyhexanenitrile (3) 40
1-phenoxy-5-(phenylsulfonyl)pentan-2-ol (6) 41
5-hydroxy-5-phenylpentanenitrile (7) 42
5-(4-fluorophenyl)-5-hydroxypentanenitrile (8) 43
5-hydroxynonanenitrile (9) 44
5-hydroxypentadecanenitrile (10) 45
5-hydroxy-6-(naphthalen-2-yloxy)hexanenitrile (11) 46
tert-butyl ((2S)-6-cyano-3-hydroxy-1-phenylhexan-2-yl)carbamate (12) 47
benzyl (5-cyano-2-hydroxypentyl)carbamate (13) 48
5-hydroxy-7-(phenylsulfonyl)heptanenitrile (14) 49
N-(1-cyanooctan-4-yl)-4-methylbenzenesulfonamide (5) 50
4-methyl-*N*-(9-oxodecan-5-yl)benzenesulfonamide (15) 51
4-methyl-*N*-(1-(phenylsulfonyl)octan-4-yl)benzenesulfonamide (16) 52
N-(1-cyanotetradecan-4-yl)-4-methylbenzenesulfonamide (17) 53
4-methyl-*N*-(2-oxohexadecan-6-yl)benzenesulfonamide (18) 54
N-(cyclopent-2-en-1-yl)-4-methylbenzenesulfonamide (19) 55
N-(cyclohex-2-en-1-yl)-4-methylbenzenesulfonamide (20) 55

1. General information

General Procedures. Unless otherwise noted, reactions were performed without the exclusion of air or moisture. Photochemical reactions were performed in 10 mL glassy vials sealed with aluminum caps containing a rubber septa or glass reaction tube (inner diameter = 18 mm). Reactions were monitored by gas chromatography (GC, specification below) or thin-layer chromatography (TLC) on Merck silica gel (GF254, 0.20 mm thickness), visualizing with UV-light or ceric ammonium molybdate (CAM)/Hanessian's stain. Colum chromatography was performed using Merck silica gel 60 (230-400 mesh) or commercially available cartridges with a CombiFlash. GC yields were calibrated using dodecane or mesitylene as an internal standard.

Materials. Commercial reagents and solvents were purchased from Sigma-Aldrich, Alfa Aesar, Fluorochem, and TCI, and used as received unless otherwise noted. Dry solvents: dimethyl sulfoxide (DMSO), dichloromethane (CH_2Cl_2), tetrahydrofuran (THF), acetonitrile (CH_3CN) were taken from Solvent Purification System (SPS). Deuterated solvent ($CDCl_3$) was purchased from Eurisotop. 4-(Phenylsulfonyl)-1,2-epoxybutane, and calatyst: (CN)(H_2O)Cby(OMe)$_7$ was synthesized according to literature procedures.[1]

Before the reaction, zinc was activated by the following method: a) washing with 10% HCl, b) grinding, c) washing with H_2O, EtOH, and Et_2O, d) drying in a vacuum.

Instrumentation.

- **NMR Spectroscopy:** ^{1}H and ^{13}C NMR spectra were recorded at 25 °C on a Bruker 400 MHz, 500 MHz or Varian 600 MHz instrument with TMS as an internal standard. NMR chemical shifts are reported in ppm and referenced to the residual solvent peak of $CDCl_3$ (7.26 ppm - ^{1}H NMR and 77.0 ppm - ^{13}C NMR). Multiplicities are indicated by singlet (s), doublet (d), triplet (t), quartet (q), multiplet (m) and broad (br). Coupling constants (*J*) are reported in Hertz. All data analysis was performed using MestReNova software package.

- **GC/MS Chromatography:** GC-MS analyses were performed using Shimadzu GCMS-QP2010 SE gas chromatograph with FID detector and Zebron ZB 5MSi column. (length: 30.0 m; thickness: 0.25 um, diameter: 0.25 mm).
  GC program time: 12.39 min; pressure: 121.8 kPa; total flow: 30.3 mL/min; column flow: 1.30 mL/min; linear velocity: 33.1 cm/s; purge flow: 3.0 mL/min; split ratio: 20.0.

	rate	temperature [°C]	hold time
0	-	100.0	1.00
1	40.00	180.0	1.50
2	40.00	260.0	1.50
3	45.00	300.0	1.00
4	50.00	325.0	2.00

- **High Resolution Mass Spectrometry:** High-resolution mass spectra (HRMS) were recorded on a Waters AutoSpec Premier instrument using electron ionization (EI) or a Waters SYNAPT G2-S HDMS instrument using electrospray ionization (ESI) with time of flight detector (TOF).

[1] Ociepa, M.; Wierzba, A. J.; Turkowska, J.; Gryko, D. Polarity-Reversal Strategy for the Functionalization of Electrophilic Strained Molecules via Light-Driven Cobalt Catalysis. *J. Am. Chem. Soc.* **2020**, *142* (11), 5355–5361. https://doi.org/10.1021/jacs.0c00245.

- **Low Resolution Mass Spectrometry:** Low-resolution mass spectra (LRMS) were recorded on an Applied Biosystems API 365 mass spectrometer using electrospray ionization (ESI) technique.

- **CombiFlash:** Products were purified using CombiFlash NextGen 300+ system with 12-inch display, 1-300 mL/min, 300 psi (with automatic injection valve).

  Flash program: time: 30.0; column: silica 4g; flow rate: 13 mL/min; automatic peak hold: on.

entry	time [min]	hexane [%]	AcOEt [%]
1	0	100	0
2	5	100	0
3	7	92	8
4	12	92	8
5	13	90	10
6	14	90	10
7	15	80	20
8	21	80	20
9	30	0	100

2. Setup for photoreactions

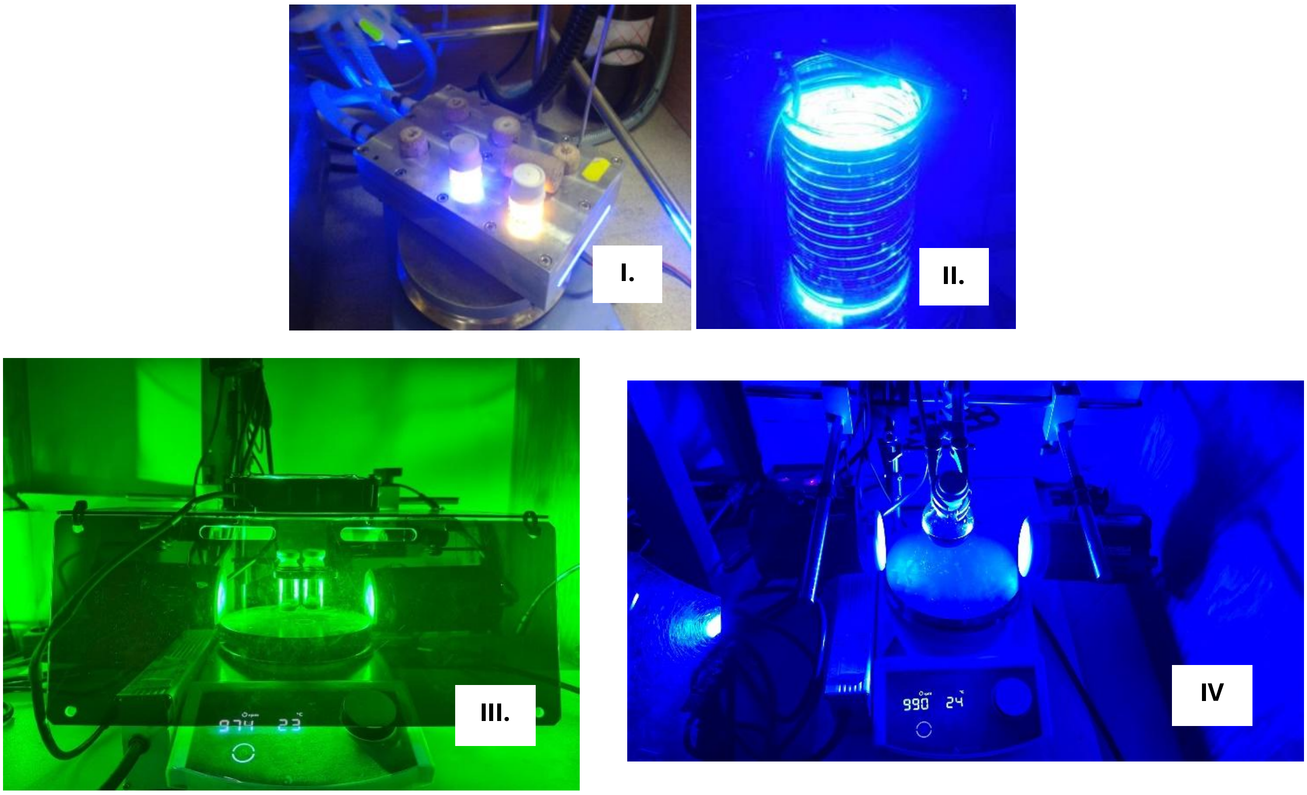

Figure 1. Photograph of photoreactors

<u>Characteristics of photoreactors:</u>

I. Single diode (LT-2855 royal blue, λmax: 446 nm, 3W), controlled by mini chiller set up at 25 ˚C, reactions in 10 mL vials, distance from the reaction vessel: 6 mm.

II. Reactions were carried out in homemade photoreactors made of 400 mL beakers covered on the inside with LED tape. A cooling fan with an adjustable spin rate was used to maintain ambient temperature inside the photoreactor.

<u>Blue LED tape:</u> 8 mm SMD3528 LED strip, 60 LED diodes/m Power consumption: 4.8 W/m blue light – λmax = 460 nm, 4.5 lm.

III and IV. Commercially available Kessil lamps were used. Blue (emission maximum at 440 nm) and green (emission maximum at 525 nm) LED light was supplied to each reaction vial using two Kessil lamps (each with a total intensity of 40 W at 100% power), placed on opposite sides with specially design cooling fan system.

Green Kessil lamps were used for the aziridine ring-opening reactions, while blue Kessil lamps were used for the 1 mmol scale-up reaction.

3. Surfactants and their abbreviations

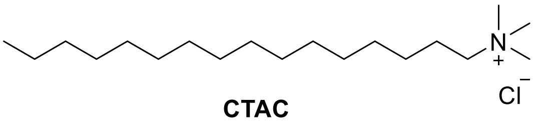

CTAC

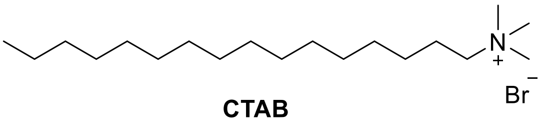

CTAB

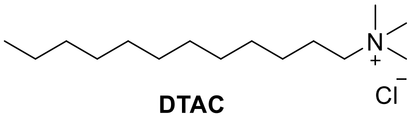

DTAC

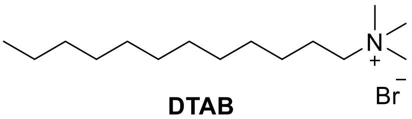

DTAB

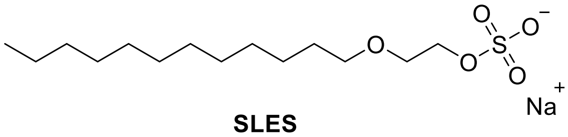

SLES

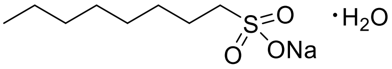

Sodium 1-octasulfonate monohydrate

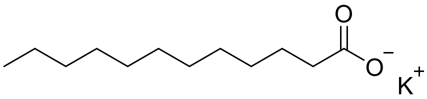

Potassium laurate

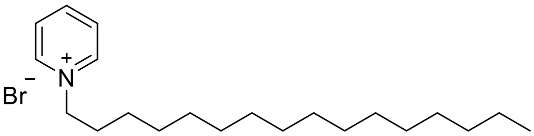

Hexadecylpyridinium bromide

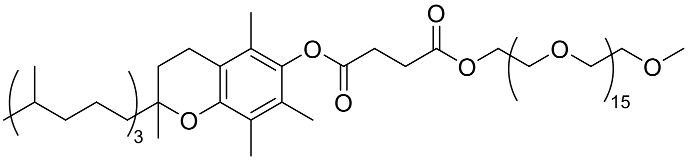

TPGS-750-M

4. Background experiments of alkyl epoxides with olefins

Model reaction:

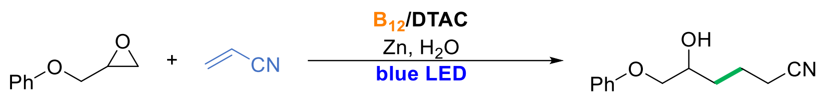

1 2 3

Entry	Deviation from the Standard Conditions	Yield of **3**[b] [%]
**1**	**none**	**85**
2	No B_{12}	0
3	No Zn	0
4	Air atmosphere	0
5	No light	0
6	No DTAC	47
7	No light, 50 °C	traces

Reaction conditions: epoxide **(1)** (0.2 mmol, 1.0 equiv.), acrylonitrile (**2**) (16 mg, 1.5 equiv.), Zn (40 mg, 3.0 equiv.), B_{12} (13.5 mg, 5.0 mol%), DTAC (264 mg, 5.0 equiv.), EtOH (0.5 ml), H_2O (4.5 mL), blue LED, 16h. [b]Yields determined by GC analysis.

5. Optimization of reactions parameters of alkyl epoxides with olefins

Procedure: Each reaction was performed in a glass vial (10 mL) sealed with an aluminum cap with a rubber septum equipped with a magnetic stirring bar. It was charged with activated Zn^0 dust (40 mg, 0.6 mmol, 3.0 equiv.), DTAC (264 mg, 5.0 equiv.) and B_{12} (5.0 mol%, 13.5 mg). Then water (4.5 mL) and ethanol (0.5 mL) were added. The resulting mixture was degassed by purging with argon with simultaneous sonication in an ultrasonic bath for 15 min. An epoxide (0.2 mmol, 1.0 equiv.) was added dropwise *via* a syringe followed by a Michael acceptor (1.5 equiv.). The resulting mixture was irradiated with blue LED light (single diode, 3 W; λ = 460 nm at room temperature) for 16 h. The resulting mixture was diluted with AcOEt (~ 3 mL) and washed with brine (20 mL). The organic phase was dried over Na_2SO_4, then filtered through the cotton wool and concentrated *in vacuo*. A crude product was purified by means of column chromatography.

Model reaction:

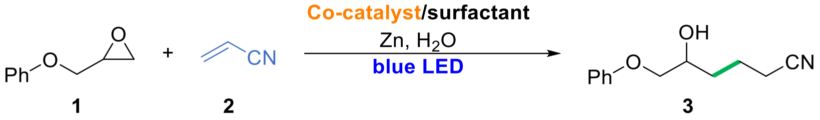

Reaction conditions: epoxide **(1)** (0.2 mmol, 1.0 equiv.), acrylonitrile (**2**) (16 mg, 1.5 equiv.), Zn (40 mg, 3.0 equiv.), Co- catalyst (5.0 mol%), DTAC (264 mg, 5.0 equiv.), EtOH (0.5 ml), H_2O (4.5 mL), blue LED, 16 h.

5.1. The influence of light

Entry	Light	Yield of **3**[b] [%]
1	White LEDs (tape)	19
2	Blue LEDs (tape)	42
3	Green LEDs (tape)	37
4	Violet LEDs (tape)	40
**5**	**Blue LED (single diode, 3 W)**	**43**
6	Green LED (single diode, 3 W)	14
7	Blue LED (single diode, 7 W)	24
8	Blue LED (single diode, 25 W)	39
9	Blue Kessil lamp (40 W)	<10

Reaction conditions: epoxide (**1**) (0.2 mmol, 1.0 equiv.), acrylonitrile (**2**) (16 mg, 1.5 equiv.), Zn (40 mg, 3.0 equiv.), NH_4Cl (32 mg, 3.0 equiv.), HME (12 mg, 5.0 mol%), MeCN (c = 0.1 M), 16 h. [b]Yields determined by GC analysis.

5.2. Screening of surfactants

A) HME-catalyzed reactions

Entry	Surfactants	Yield of **3**[b] [%]
1	CTAC	10
**2**	**DTAC**	**33**
3	CTAB	25
4	DOSS	15
5	SLES	25
6	Potassium laurate	10
7	TPGS-750-M	13

Reaction conditions: epoxide (**1**) (0.2 mmol, 1.0 equiv.), acrylonitrile (**2**) (16 mg, 1.5 equiv.), Zn (40 mg, 3.0 equiv.), NH_4Cl (32 mg, 3.0 equiv.), HME (12 mg, 5.0 mol%), surfactant (2.5 eq.), MeCN (c = 0.1 M), Blue LED (single diode 3 W), 16 h. [b]Yields determined by GC analysis.

B) B_{12}-catalyzed reactions

Entry	Surfactants	Yield of **3**[b] [%]
1	DTAC	64
**2**	**DTAC (without NH_4Cl)**	**59**
3	CTAB	73
4	CTAB (without NH_4Cl)	59
5	SLES	46
6	Potassium laurate	45
7	OTAI	56
8	CTAC	58
9	DTAB	47
10	1-Hexadecylpyridinium bromide	31

Reaction conditions: epoxide (**1**) (0.2 mmol, 1.0 equiv.), acrylonitrile (**2**) (16 mg, 1.5 equiv.), Zn (40 mg, 3.0 equiv.), NH_4Cl (32 mg, 3.0 equiv.), B_{12} (13.5 mg, 5.0 mol%), surfactant (2.5 eq.), H_2O (5 mL), Blue LED (single diode 3 W), 16 h. [b]Yields determined by GC analysis.

C) The amount of the surfactant

Entry	DTAC (equiv.)	Yield of **3**[b] [%]
1	1	68
2	2.5	59
**3**	**5**	**76**
4	7.5	64
5	10	69

Reaction conditions: epoxide (**1**) (0.2 mmol, 1.0 equiv.), acrylonitrile (**2**) (16 mg, 1.5 equiv.), Zn (40 mg, 3.0 equiv.), B_{12} (13.5 mg, 5.0 mol%), DTAC, H_2O (5 mL), Blue LED (single diode 3 W), 16 h. [b]Yields determined by GC analysis.

5.3. B_{12} – Impact of the catalyst loading

Entry	Catalyst loading [%]	Yield of **3**[b] [%]
1	2.5	66
**2**	**5**	**76**
3	7.5	67
4	10	58

Reaction conditions: epoxide **(1)** (0.2 mmol, 1.0 equiv.), acrylonitrile (**2**) (16 mg, 1.5 equiv.), Zn (40 mg, 3.0 equiv.), B_{12}, DTAC (264 mg, 5.0 equiv.), H_2O (5 mL), Blue LED (single diode 3 W), 16 h. [b]Yields determined by GC analysis.

5.4. Optimization of the substrates' ratio

Entry	**1** (equiv.)	**2** (equiv.)	Yield of **3**[b] [%]
**1**	**1**	**1.5**	**76**
2	1	2	61
3	1	3	65
4	2	1	60
5	1	1	72

Reaction conditions: epoxide **(1),** acrylonitrile (**2**), Zn (40 mg, 3.0 equiv.), B_{12} (13.5 mg, 5.0 mol%), DTAC (264 mg, 5.0 equiv.), H_2O (5 mL), Blue LED (single diode 3 W), 16 h. [b]Yields determined by GC analysis.

5.5. The influence of the amount of Zn

Entry	Zn (equiv.)	Yield of **3**[b] [%]
1	1	65
2	1.5	64
**3**	**3**	**76**
4	5	65

Reaction conditions: epoxide (**1**) (0.2 mmol, 1.0 equiv.), acrylonitrile (**2**) (16 mg, 1.5 equiv.), Zn, B_{12} (13.5 mg, 5.0 mol%), DTAC (264 mg, 5.0 equiv.), H_2O (5 mL), Blue LED (single diode 3 W), 16 h. [b]Yields determined by GC analysis.

5.6. Concentration of epoxide (1)

Entry	[mol/dm^3]	Yield of **3**^{b} [%]
1	0.08	70
**2**	**0.04**	**76**
3	0.03	70

Reaction conditions: epoxide (**1**) (0.2 mmol, 1.0 equiv.), acrylonitrile (**2**) (16 mg, 1.5 equiv.), Zn (40 mg, 3.0 equiv.), B_{12} (13.5 mg, 3.0 equiv.), DTAC (264 mg, 5.0 equiv.), H_2O, Blue LED (single diode 3 W), 16 h. ^{b}Yields determined by GC analysis.

5.7. Screening of additives

Entry	Additives	Yield of **3**^{b} [%]
1	-	76
2	MeOH	76
**3**	**EtOH**	**85**
4	Propanol	81
5	*i*-Propanol	80
6	Butanol	77
7	*t*-Butanol	64

Reaction conditions: epoxide (**1**) (0.2 mmol, 1.0 equiv.), acrylonitrile (**2**) (16 mg, 1.5 equiv.), Zn (40 mg, 3.0 equiv.), B_{12} (13.5 mg, 5.0 mol%), DTAC (264 mg, 5.0 equiv.), H_2O (4.5 mL), additive (0.5 mL), Blue LED (single diode 3 W), 16 h. ^{b}Yields determined by GC analysis.

6. Optimization of reactions parameters of aryl epoxides with olefins

Procedure: Each reaction was performed in a glass vial (10 mL) sealed with an aluminum cap with a rubber septum equipped with a magnetic stirring bar. It was charged with activated Zn^0 dust (40 mg, 0.6 mmol, 3.0 equiv.), DTAC (132 mg, 2.5 equiv.) and B_{12} (5.0 mol%, 13.5 mg). Then water (4.5 mL) and ethanol (0.5mL) were added. The resulting mixture was degassed by purging with argon with simultaneous sonication in an ultrasonic bath for 15 min. An epoxide (0.2 mmol, 1.0 equiv.) was added dropwise *via* a syringe followed by a Michael acceptor (5.0 equiv.). The resulting mixture was irradiated with blue LED light (tape, 32 W; λ = 460 nm) at room temperature for 16 h. The resulting mixture was diluted with AcOEt (~ 3 mL) and washed with brine (20 mL). The organic phase was dried over Na_2SO_4, then filtered through the cotton wool and concentrated *in vacuo*. A crude product was purified by means of column chromatography.

Model reaction:

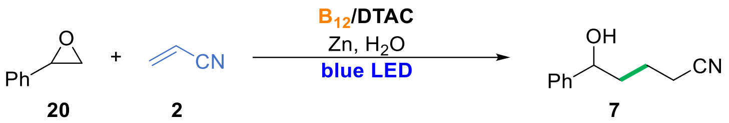

Reaction conditions: epoxide (**20**) (0.1 mmol, 1.0 equiv.), acrylonitrile (**2**) (16 mg, 1.5 equiv.), Zn (40 mg, 3.0 equiv.), B_{12} (13.5 mg, 5.0 mol%), DTAC (132 mg, 2.5 equiv.), EtOH (0.5 ml), H_2O (4.5 mL), Blue LED, 16h.

6.1. The influence of light

Entry	Light	Yield of **7**[b] [%]
**1**	**Blue LEDs (tape)**	**40**
2	Blue LED (single diode, 3 W)	27
3	Blue LED (single diode, 7 W)	20
4	Blue LED (single diode, 25 W)	35
5	Blue LED (single diode, 10 W, 1 h)	<10

Reaction conditions: epoxide (**20**) (0.2 mmol, 1.0 equiv.), acrylonitrile (**2**) (16 mg, 1.5 equiv.), Zn (40 mg, 3.0 equiv.), B_{12} (13.5 mg, 5.0 mol%), DTAC (264 mg, 5.0 equiv.), H_2O (4.5 mL), EtOH (0.5 mL), 16 h. [b]Yields determined by GC analysis.

6.2. Screening of surfactant

Entry	DTAC (equiv.)	Yield of **7**[b] [%]
1	1	35
**2**	**2.5**	**41**
3	5	40
4	7.5	29
5	10	35

Reaction conditions: epoxide (**20**) (0.2 mmol, 1.0 equiv.), acrylonitrile (**2**) (16 mg, 1.5 equiv.), Zn (40 mg, 3.0 equiv.), B_{12} (13.5 mg, 5.0 mol%), DTAC, H_2O (4.5 mL), EtOH (0.5 mL), Blue LED, 16 h. [b]Yields determined by GC analysis.

6.3. The influence of the amount of Zn

Entry	Zn (equiv.)	Yield of **7**[b] [%]
1	1	22
2	1.5	34
**3**	**3**	**41**
4	5	38

Reaction conditions: epoxide (**20**) (0.2 mmol, 1.0 equiv.), acrylonitrile (**2**) (16 mg, 1.5 equiv.), Zn, B_{12} (13.5 mg, 5.0 mol%), DTAC (132 mg, 2.5 equiv.), H_2O (4.5 mL), EtOH (0.5 mL), Blue LED, 16 h. [b]Yields determined by GC analysis.

6.4. Screening of additives

Entry	Additives	Yield of **7**[b] [%]
1	-	24
2	MeOH	26
**3**	**EtOH**	**41**
4	Propanol	25
5	*i*-Propanol	32
6	Butanol	31
7	*t*-Butanol	27

Reaction conditions: epoxide (**20**) (0.2 mmol, 1.0 equiv.), acrylonitrile (**2**) (16 mg, 1.5 equiv.), Zn (40 mg, 3.0 equiv.), B_{12} (13.5 mg, 5.0 mol%), DTAC (132 mg, 2.5 equiv.), H_2O (4.5 mL), additive (0.5 mL), Blue LED (single diode 3 W), 16 h. [b]Yields determined by GC analysis.

6.5. The amount of EtOH

Entry	EtOH v/v	Yield of **7**[b] [%]
1	5 %	31
**2**	**10 %**	**41**
3	20 %	33
4	40 %	16
5	50 %	30

Reaction conditions: epoxide (**20**) (0.2 mmol, 1.0 equiv.), acrylonitrile (**2**) (16 mg, 1.5 equiv.), Zn (40 mg, 3.0 equiv.), B_{12} (13.5 mg, 5.0 mol%), DTAC (132 mg, 2.5 equiv.), H_2O (4.5 mL), EtOH, Blue LED, 16 h. [b]Yields determined by GC analysis.

6.6. B_{12} – Impact of the catalyst loading

Entry	Catalyst loading [%]	Yield of **7**[b] [%]
1	2.5	31
**2**	**5**	**41**
3	10	15

Reaction conditions: epoxide (**20**) (0.2 mmol, 1.0 equiv.), acrylonitrile (**2**) (16 mg, 1.5 equiv.), Zn (40 mg, 3.0 equiv.), B_{12}, DTAC (132 mg, 2.5 equiv.), H_2O (4.5 mL), EtOH (0.5 ml), Blue LED (single diode 3 W), 16 h. [b]Yields determined by GC analysis.

6.7. Optimization of the substrates' ratio

Entry	**20** (equiv.)	**2** (equiv.)	Yield of **7**[b] [%]
1	1	1.5	41
2	1	3	35
**3**	**1**	**5**	**48**
4	1	10	39
5	1	1	17

Reaction conditions: epoxide (**20**), acrylonitrile (**2**), Zn (40 mg, 3.0 equiv.), B_{12} (13.5 mg, 5.0 mol%), DTAC (132 mg, 2.5 equiv.), H_2O (4.5 mL), EtOH (0.5 ml), Blue LED (single diode 3 W), 16 h. [b]Yields determined by GC analysis.

7. Background experiments for alkyl aziridine reaction with olefin

<u>Model reaction:</u>

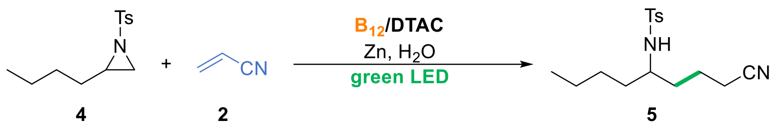

Entry	Deviation from the Standard Conditions	Yield of **5**[b] [%]
**1**	**none**	**83**
2	No Co-cat.	0
3	No Zn	0
4	No light	0
5	No DTAC	29

Reaction conditions: aziridine (**4**) (0.2 mmol, 1.0 equiv.), acrylonitrile (**2**) (16 mg, 1.5 equiv.), Zn (40 mg, 3.0 equiv.), B_{12} (7 mg, 2.5 mol%), DTAC (185 mg, 3.5 equiv.), *i*PrOH (0.5 ml), H_2O (4.5 mL), green LED, 24 h. [b]Yields determined by GC analysis.

8. Optimization of reactions parameters of alkyl aziridines with olefins

Procedure: Each reaction was performed in a glass vial (10 mL) sealed with an aluminum cap with a rubber septum equipped with a magnetic stirring bar. It was charged with activated Zn^0 dust (40 mg, 0.6 mmol, 3.0 equiv.), DTAC (185 mg, 3.5 equiv.) and B_{12} (2.5 mol%, 6.75 mg). Then water (4.5 mL) and isopropanol (0.5 mL) were added. The resulting mixture was degassed by purging with argon with simultaneous sonication in an ultrasonic bath for 25 min. Then, aziridine (0.2 mmol, 1.0 equiv.) was added dropwise *via* a syringe followed by a Michael acceptor (3.0 equiv.). The resulting mixture was irradiated with green LED light (Kessil, 40 W; λ = 525 nm) for 24 h at room temperature. The resulting mixture was diluted with AcOEt (~ 3 mL) and washed with brine (20 mL). The organic phase was dried over Na_2SO_4, then filtered through the cotton wool and concentrated *in vacuo*. A crude product was purified by means of column chromatography.

Model reaction:

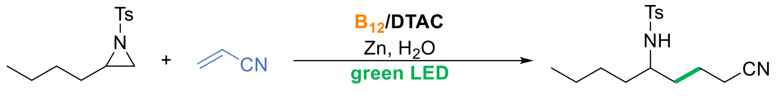

Reaction conditions: aziridine (**4**) (0.2 mmol, 1.0 equiv.), acrylonitrile (**2**) (16 mg, 1.5 equiv.), Zn (40 mg, 3.0 equiv.), B_{12} (7 mg, 2.5 mol%), DTAC (185 mg, 3.5 equiv.), *i*PrOH (0.5 ml), H_2O (4.5 mL), green LED, 24 h.

8.2. The influence of light

Entry	Light	Yield of **5**[b] [%]
1	White LEDs (tape)	31
2	Yellow LEDs (tape)	26
3	Green LEDs (tape)	37
4	Violet LEDs (tape)	43
**5**	**Green Kessil lamp (40 W)**	**61**
6	Blue Kessil lamp (40 W)	38
7	Green LED (23 W)	60
8	Green LED (single diode, 3 W)	56
9	Blue LED (single diode, 3 W)	57
10	Blue LED (single diode, 7 W)	51

Reaction conditions: aziridine (**4**) (0.2 mmol, 1.0 equiv.), acrylonitrile (**2**) (16 mg, 1.5 equiv.), Zn (40 mg, 3.0 equiv.), B_{12} (13.5 mg, 5.0 mol%), DTAC (264 mg, 5.0 equiv.), H_2O (5 mL), X LED, 24 h. [b]Yields determined by GC analysis.

8.3. Screening of surfactants

Entry	Surfactants	Yield of **5**[b] [%]
1	DTAB	60
**2**	**DTAC**	**61**
3	CTAB	43
4	CTAC	54
5	SLES	50
6	Potassium Laurate	38
7	Sodium 1-octanesulfonate monohydrate	39
8	TPGS-750-M	52
9	1-Hexadecylpyridinium bromide	49

Reaction conditions: aziridine (**4**) (0.2 mmol, 1.0 equiv.), acrylonitrile (**2**) (16 mg, 1.5 equiv.), Zn (40 mg, 3.0 equiv.), B_{12} (13.5 mg, 5.0 mol%), X (5.0 equiv.), H_2O (5 mL), Green LED, 24 h. [b]Yields determined by GC analysis.

B) The amount of the surfactant

Entry	DTAC (equiv.)	Yield of **5**[b] [%]
1	1	37
2	1.5	38
3	2.5	50
4	3	56
**5**	**3.5**	**57**
6	5	64
7	7	56

Reaction conditions: aziridine (**4**) (0.2 mmol, 1.0 equiv.), acrylonitrile (**2**) (16 mg, 1.5 equiv.), Zn (40 mg, 3.0 equiv.), B_{12} (13.5 mg, 5.0 mol%), DTAC (X equiv.), H_2O (5 mL), Green LED, 24 h. [b]Yields determined by GC analysis.

8.4. B_{12} – Impact of the catalyst loading

Entry	Catalyst loading [%]	Yield of **5**[b] [%]
**1**	**2.5**	**61**
2	5	60
3	7.5	61
4	10	63

Reaction conditions: aziridine (**4**) (0.2 mmol, 1.0 equiv.), acrylonitrile (**2**) (16 mg, 1.5 equiv.), Zn (40 mg, 3.0 equiv.), B_{12} (X mol%), DTAC (185 mg, 3.5 equiv.), H_2O (5 mL), Green LED, 24 h. [b]Yields determined by GC analysis.

8.5. Optimization of the substrates' ratio

Entry	**4** (equiv.)	**2** (equiv.)	Yield of **5**[b] [%]
1	1	1	47
**2**	**1**	**1.5**	**57**
3	1	2	37
4	2	1	58
5	1	2.5	36
6	1	3	38

Reaction conditions: aziridine (**4**) (X), acrylonitrile (**2**) (X), Zn (40 mg, 3.0 equiv.), B_{12} (6.75 mg, 2.5 mol%), DTAC (185 mg, 3.5 equiv.), H_2O (5 mL), Green LED, 24 h. [b]Yields determined by GC analysis.

8.6. The influence of the amount of Zn

Entry	Zn (equiv.)	Yield of **5**[b] [%]
1	1	52
2	1.5	54
**3**	**3**	**57**
4	5	42

Reaction conditions: aziridine (**4**) (0.2 mmol, 1.0 equiv.), acrylonitrile (**2**) (16 mg, 1.5 equiv.), Zn (X), B_{12} (7 mg, 2.5 mol%), DTAC (185 mg, 3.5 equiv.), H_2O (5 mL), Green LED, 24 h. [b]Yields determined by GC analysis.

8.7. Concentration of aziridine

Entry	[mol/dm^3]	Yield of **5**[b] [%]
1	0.02	43
**2**	**0.04**	**57**
3	0.06	52
4	0.08	42

Reaction conditions: aziridine (**4**) (0.2 mmol, 1.0 equiv.), acrylonitrile (**2**) (16 mg, 1.5 equiv.), Zn (40 mg, 3.0 equiv.), B_{12} (7 mg, 2.5 mol%), DTAC (185 mg, 3.5 equiv.), H_2O (X), Green LED, 24 h. [b]Yields determined by GC analysis.

8.8. Screening of additives

Entry	Additives	Yield of **5**[b] [%]
1	-	57
2	MeOH	55
3	EtOH	49
4	Propanol	64
**5**	***i*-Propanol**	**83**
6	Butanol	59
7	*t*-Butanol	61

Reaction conditions: aziridine (**4**) (0.2 mmol, 1.0 equiv.), acrylonitrile (**2**) (16 mg, 1.5 equiv.), Zn (40 mg, 3.0 equiv.), B_{12} (7 mg, 2.5 mol%), DTAC (185 mg, 3.5 equiv.), H_2O (4.5 ml), additive (0.5 ml), Green LED, 24 h. [b]Yields determined by GC analysis.

9. Mechanistic consideration

9.1. Proposed mechanism

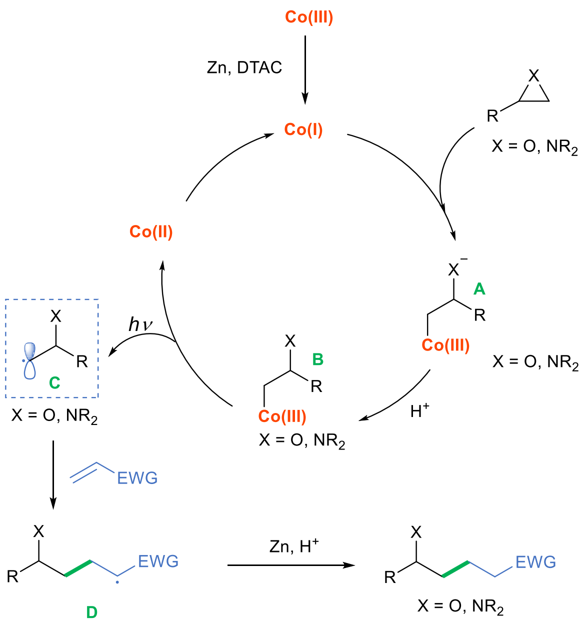

9.2. Kinetic studies of the model alkyl epoxide

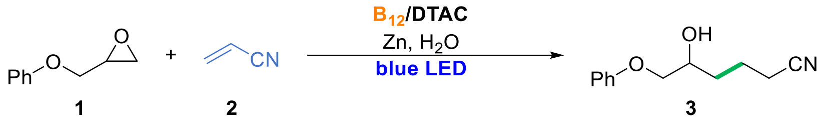

Reaction conditions: epoxide (**1**) (0.2 mmol, 1.0 equiv.), acrylonitrile (**2**) (16 mg, 1.5 equiv.), Zn (40 mg, 3.0 equiv.), B_{12} (13.5 mg, 5.0 mol%), DTAC (264 mg, 5.0 equiv.), H_2O (4.5 mL), additive (0.5 mL), Blue LED (single diode 3 W).

The reaction of the model alkyl epoxide with the olefin was set up according to the general procedure **A** on 0.2 mmol scale with the addition of dodecane as an internal standard. The reaction was monitored by GC/FID for 24 h.

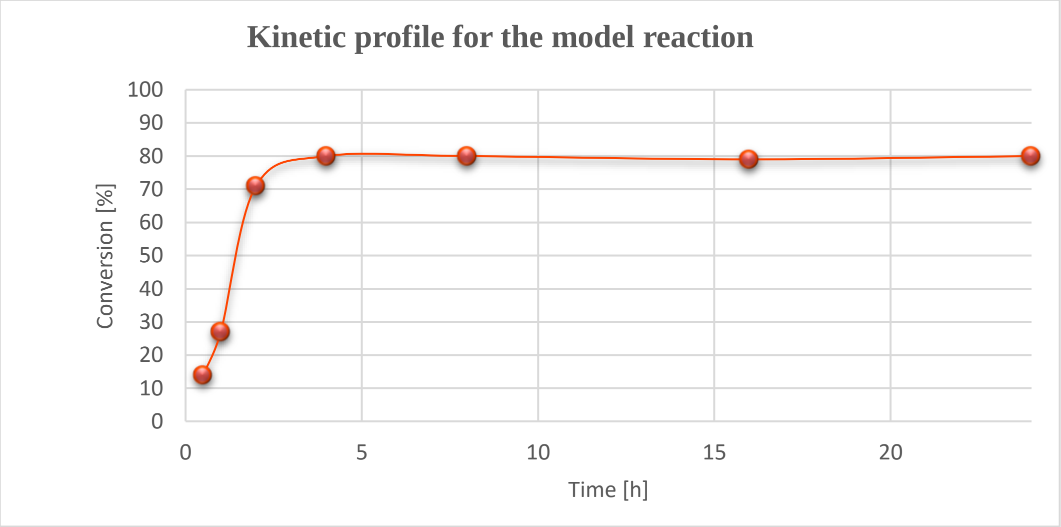

Figure 2. The rate of the conversion of 5-hydroxy-6-phenoxyhexanenitrile (**3**)

<u>Conclusion</u>: Kinetic studies indicated an optimal time of 4 h for product 3 formation, with no significant decomposition observed subsequent to 4 hours of reaction time.

9.3. Co(III)-alkyl complex formation

A) Epoxide

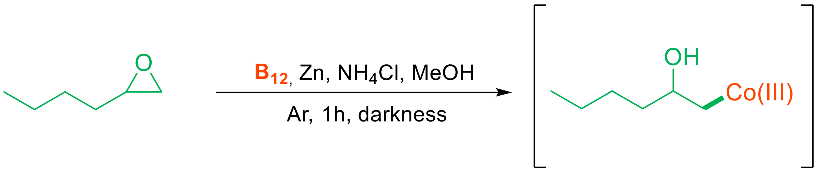

22

Reaction conditions: 2-butyloxirane (0.05 mmol, 1.0 equiv.), vitamin B_{12} (0.05 mmol, 1.0 equiv.), Zn (1.50 mmol, 30 equiv.), NH_4Cl (1.50 mmol, 30 equiv.), MeOH (1 mL), darkness, 1h.

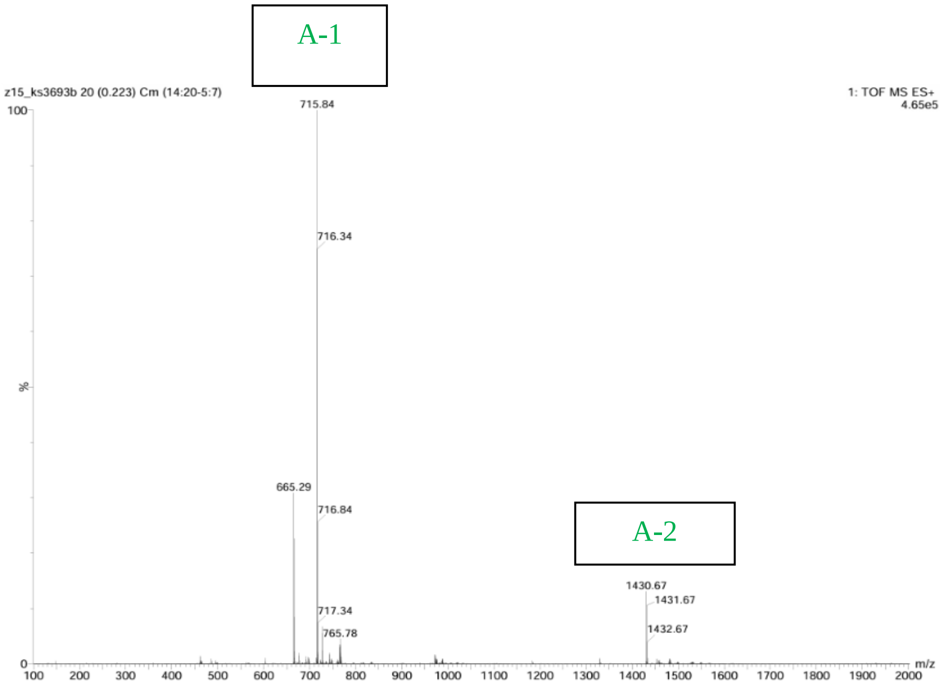

Signal A-1

$C_{63}H_{102}CoN_{13}O_{15}P^{2+}$

Calcd mass = 715,84

Found = 715,84

Signal A-2

$C_{63}H_{102}CoN_{13}O_{15}P^{+}$

Calcd mass = 1430,67

Found = 1430,67

The HRMS ESI (+) spectrum of the reaction mixture indicates the presence of two main forms of the alkylcobalamin complexes (signals A-1 and A-2). Signals A-1 and A-2 correspond to the mass of alkylcobalamin complex.

Conclusion: This experiment supports the hypothesis that the reaction involves an alkyl-cobalt complex.

B) Aziridine

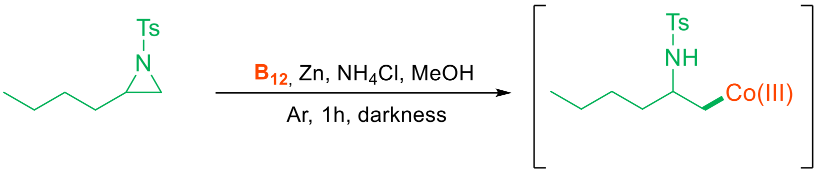

23

Reaction conditions: 2-butyl-1-tosylaziridine (0.05 mmol, 1.0 equiv.,), vitamin B_{12} (0.05 mmol, 1.0 equiv.), Zn (1.50 mmol, 30 equiv.), NH_4Cl (1.50 mmol, 30 equiv.), MeOH (1 mL), darkness, 1h.

A glass reaction tube equipped with a magnetic bar was charged with vitamin B_{12} (68 mg, 0.05 mmol, 1 equiv.) ammonium chloride (80 mg, 1.50 mmol, 30 equiv.) and activated zinc (98 mg, 1.50 mmol, 30.0 equiv.), then MeOH (1 mL) was added. Tube was sealed with a septum and the resulting mixture was degassed by purging the solution with argon for 20 minutes with simultaneous sonication in ultrasonic bath (the solution turned from pink to dark brown). Subsequently, the reaction tube was then sealed with aluminum foil, epoxide or aziridine (0.05 mmol, 1.0 equiv.) was added and the reaction was placed on a magnetic stirrer. After 60 minutes an aliquot was taken from the reaction mixture and its composition was studied by HRMS ESI(+).

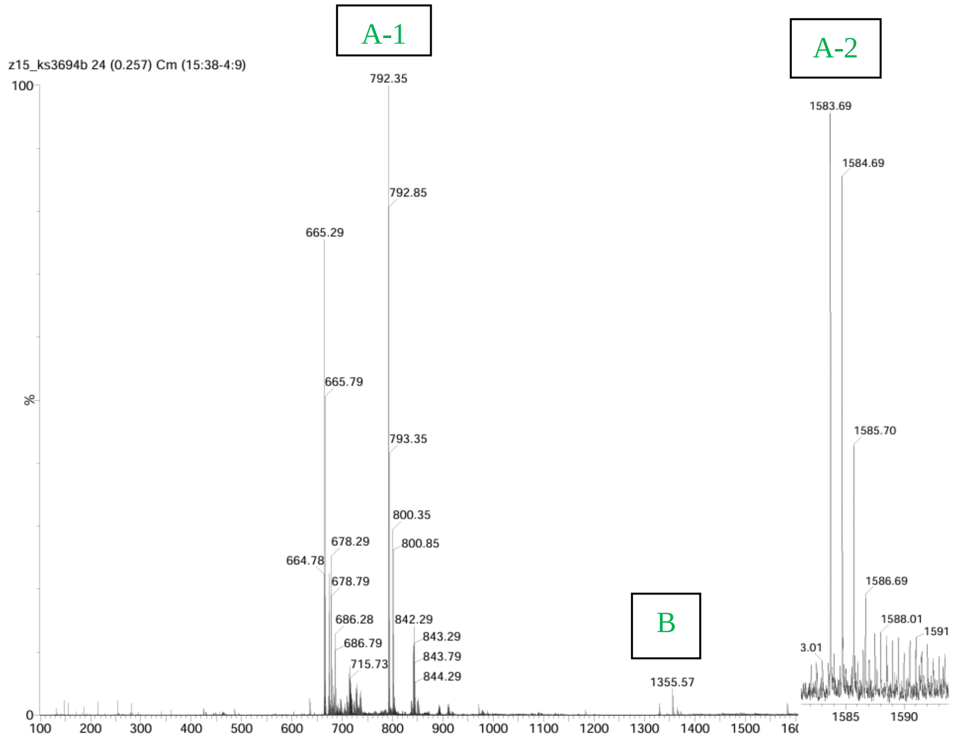

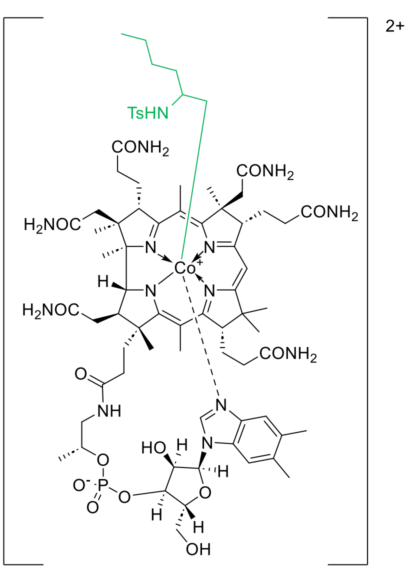

Signal A-1
$C_{75}H_{109}CoN_{14}O_{16}SP^{2+}$

Calcd mass = 792,35
Found = 792,35

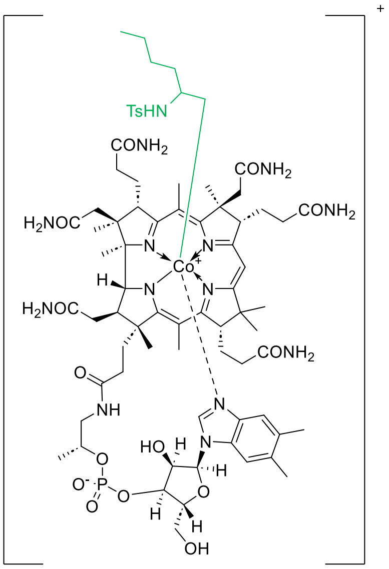

Signal A-2
$C_{75}H_{109}CoN_{14}O_{16}SP^{+}$

Calcd mass = 1583,69
Found = 1583,69

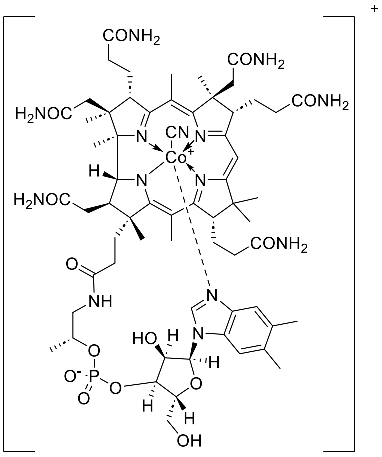

Signal B
$C_{63}H_{88}CoN_{14}O_{14}P^{+}$

Calcd mass = 1355,57
Found = 1355,57

The HRMS ESI (+) spectrum of the reaction mixture indicates the presence of three main forms of alkylcobalamin complexes (signals A-1-2 and B). Signals A-1 and A-2 correspond to the mass of alkylcobalamin complex. Signal B corresponds to the mass of the catalyst.

<u>Conclusion</u>: This experiment supports the hypothesis that the reaction involves an alkyl-cobalt complex.

9.4. Experiment with a radical trap

A) Epoxide

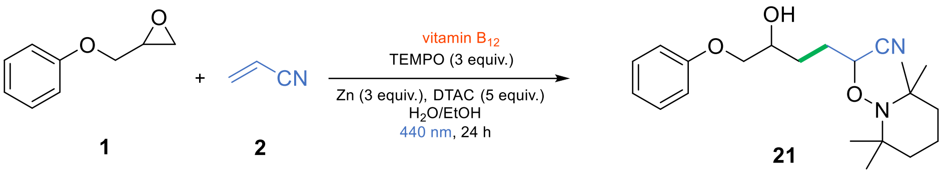

1 **2** **21**

Tempo-adduct

Reaction conditions: epoxide (**1**) (0.2 mmol, 1.0 equiv.), acrylonitrile (**2**) (16 mg, 1.5 equiv.), Zn (40 mg, 3.0 equiv.), B_{12} (13.5 mg, 5.0 mol%), DTAC (264 mg, 5.0 equiv.), H_2O (4.5 mL), EtOH (0.5 mL), 24 h, blue LED (single diode 3 W).

The reaction was set up following the general procedure A (in 4 mL of H_2O). Subsequently (after 30 min), TEMPO (3 equiv., 0.60 mmol in 1 mL of DTAC solution in H_2O) was added. Then the reaction was worked up as usual. HRMS ESI(+) analysis of the crude reaction mixture indicates the formation of the TEMPO adduct with the radical.

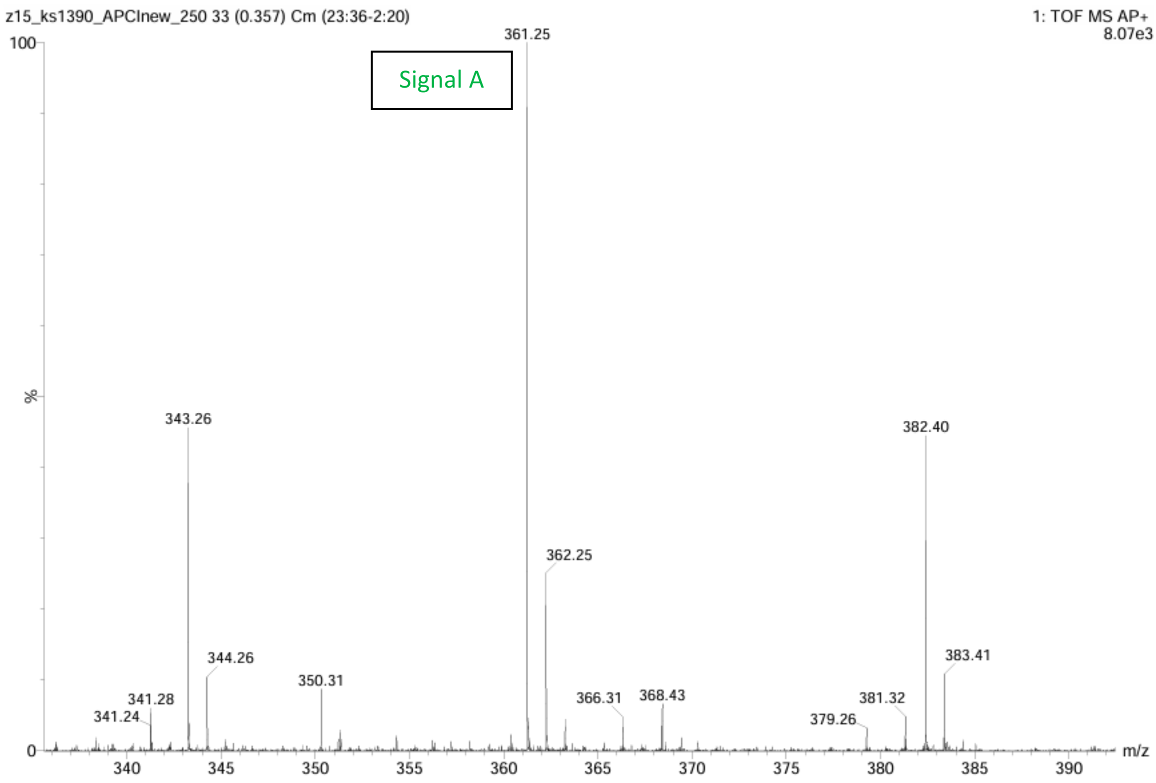

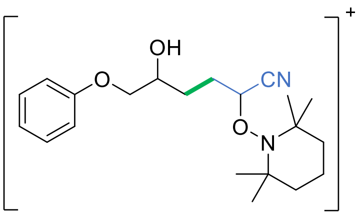

Signal A

Chemical formula: $C_{21}H_{33}N_2O_3^+$

Exact Mass: 361.2491

Found: 361.2492

The HRMS ESI (+) spectrum of the reaction mixture indicates the presence of the radical adduct (signal A). Signal A corresponds to the mass of product/TEMPO adduct **21**.

Conclusion: This experiment supports the hypothesis that the reaction involves a radical as an intermediate.

B) Aziridine

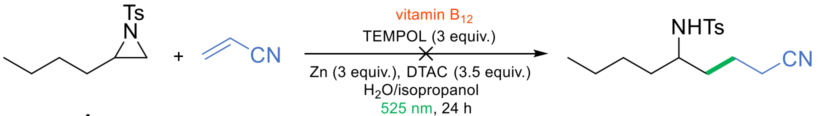

4 **2** **5** 0%

Reaction conditions: aziridine (0.2 mmol, 1.0 equiv.), acrylonitrile (0.3 mmol, 1.5 equiv.), vitamin B_{12} (0.005 mmol, 2.5 mol%), Zn (0.6 mmol, 3.0 equiv.), DTAC (0.7 mmol, 3.5 equiv.), TEMPOL (0.6 mmol, 3.0 equiv.), H_2O (4.5 mL), *i*PrOH (0.5 ml) 24 h, green Kessil LED.

The reaction was set up following the general procedure **C** with the addition of TEMPOL. Then the reaction was worked up as usual. TLC showed no conversion of the starting material, GC-FID further proved that the starting material was not converted. The reaction was halted completely by the addition of a TEMPOL.

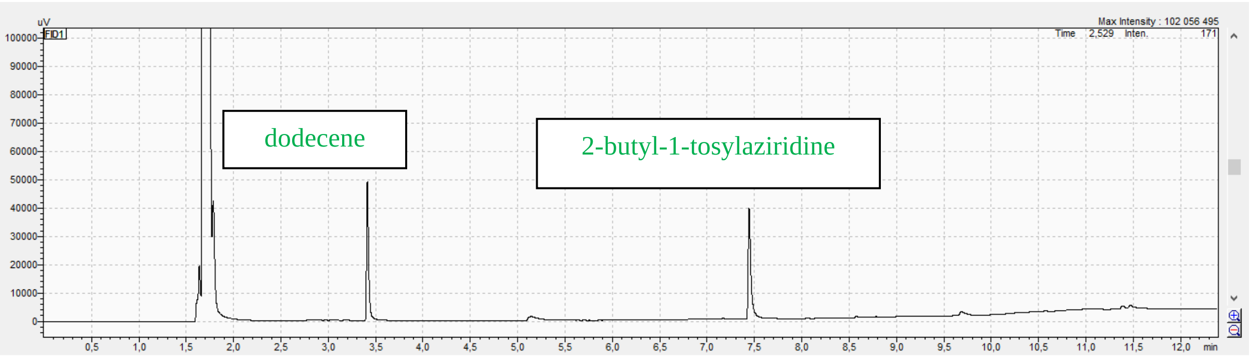

Conclusion: This experiment supports the hypothesis that a radical intermediate is involved in the reaction.

10. Preparation of starting materials (S1-S12) and characterization of new compounds

10.1. Synthesis of starting materials

Not commercially available substrates were synthesized according to the reported procedures.[2,3,4,5,6,7,8] The observed characterization data (^{1}H and ^{13}C NMR) are consistent with those previously reported.

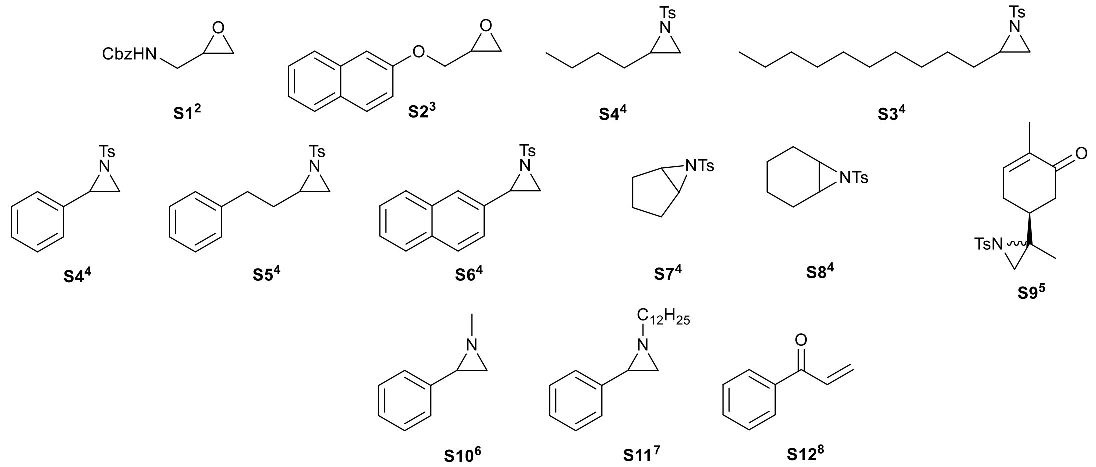

---

[2] Giordano, C.; Gallina, C.; Consalvi, V.; Scandurra, R. Irreversible Inactivation of Papain and Cathepsin B by Epoxidic Substrate Analogues. *Eur. J. Med. Chem.* **1990**, *25* (6), 479–487. https://doi.org/https://doi.org/10.1016/0223-5234(90)90142-P.

[3] Tacon, C.; Guantai, E. M.; Smith, P. J.; Chibale, K. Synthesis, Biological Evaluation and Mechanistic Studies of Totarol Amino Alcohol Derivatives as Potential Antimalarial Agents. *Bioorg. Med. Chem.* **2012**, *20* (2), 893–902. https://doi.org/https://doi.org/10.1016/j.bmc.2011.11.060.

[4] Park, S.; Koo, J.; Kim, W.; Lee, H. G. A Tandem Process for the Synthesis of β-Aminoboronic Acids from Aziridines with Haloamine Intermediates. *Chem. Commun.* **2022**, *58* (23), 3767–3770. https://doi.org/10.1039/D2CC00808D.

[5] Sureshkumar, D.; Koutha, S. M.; Chandrasekaran, S. Chemistry of Tetrathiomolybdate: Aziridine Ring Opening Reactions and Facile Synthesis of Interesting Sulfur Heterocycles. *J. Am. Chem. Soc.* **2005**, *127* (37), 12760–12761. https://doi.org/10.1021/ja052969z.

[6] Bresciani, G.; Bortoluzzi, M.; Pampaloni, G.; Marchetti, F. Diethylammonium Iodide as Catalyst for the Metal-Free Synthesis of 5-Aryl-2-Oxazolidinones from Aziridines and Carbon Dioxide. *Org. Biomol. Chem.* **2021**, *19* (18), 4152–4161. https://doi.org/10.1039/D1OB00458A.

[7] Depa WJ, Majumder S, Nadirova M, Cmoch P, Chaładaj W, Andersson MP, et al. CO2 utilization in a micellar system: synthesis of cyclic carbonates. ChemRxiv. 2024; doi:10.26434/chemrxiv-2024-jd8vk This content is a preprint and has not been peer-reviewed.

[8] Chanthamath, S.; Takaki, S.; Shibatomi, K.; Iwasa, S. Highly Stereoselective Cyclopropanation of α,β-Unsaturated Carbonyl Compounds with Methyl (Diazoacetoxy)Acetate Catalyzed by a Chiral Ruthenium(II) Complex. *Angew. Chemie Int. Ed.* **2013**, *52* (22), 5818–5821. https://doi.org/https://doi.org/10.1002/anie.201300468.

10.2. Scope limitation: unsuccessful starting materials

A) *Epoxides*

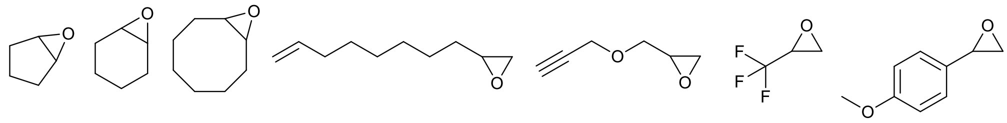

B) *Aziridines*

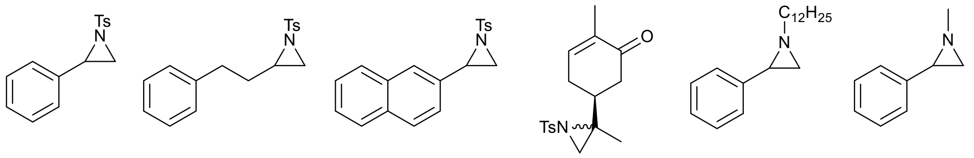

C) *Michael acceptors*

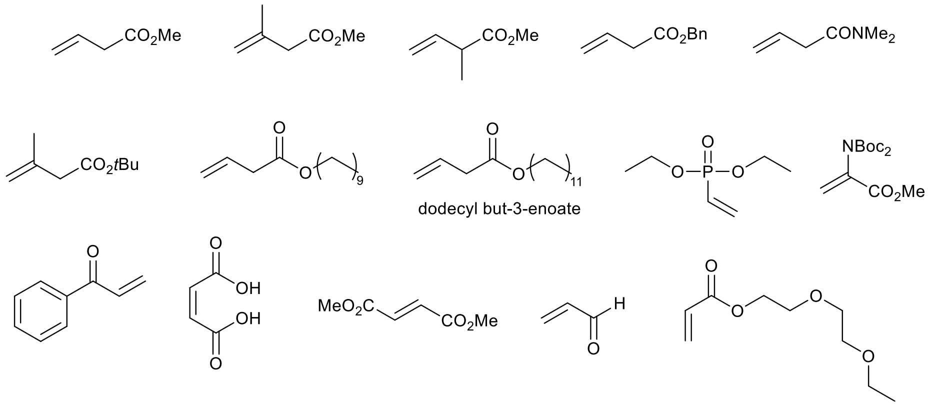

11. General Procedures

***A**. General procedure for aliphatic epoxide:*

Each reaction was performed in a glass vial (10 mL) sealed with an aluminum cap with a rubber septum equipped with a magnetic stirring bar. It was charged with activated Zn^0 dust (40 mg, 0.6 mmol, 3.0 equiv.), DTAC (264 mg, 5.0 equiv.) and B_{12} (5.0 mol%, 13.5 mg). Then water (4.5 mL) and ethanol (0.5 mL) were added. The resulting mixture was degassed by purging with argon with simultaneous sonication in an ultrasonic bath for 15 min. An epoxide (0.2 mmol, 1.0 equiv.) was added dropwise *via* a syringe followed by a Michael acceptor (1.5 equiv). The resulting mixture was irradiated with blue LED light (single diode, 3 W; λ = 460 nm at room temperature) for 16 h. The resulting mixture was diluted with AcOEt (~ 3 mL) and washed with brine (20 mL). The organic phase was dried over Na_2SO_4, then filtered through the cotton wool and concentrated *in vacuo*. A crude product was purified by means of column chromatography.

***B**. General procedure for aryl epoxide:*

Each reaction was performed in a glass vial (10 mL) sealed with an aluminum cap with a rubber septum equipped with a magnetic stirring bar. It was charged with activated Zn^0 dust (40 mg, 0.6 mmol, 3.0 equiv.), DTAC (132 mg, 2.5 equiv.) and B_{12} (5.0 mol%, 13.5 mg). Then water (4.5 mL) and ethanol (0.5mL) were added. The resulting mixture was degassed by purging with argon with simultaneous sonication in an ultrasonic bath for 15 min. An epoxide (0.2 mmol, 1.0 equiv.) was added dropwise *via* a syringe followed by a Michael acceptor (5.0 equiv). The resulting mixture was irradiated with blue LED light (tape, 32 W; λ = 460 nm) at room temperature for 16 h. The resulting mixture was diluted with AcOEt (~ 3 mL) and washed with brine (20 mL). The organic phase was dried over Na_2SO_4, then filtered through the cotton wool and concentrated *in vacuo*. A crude product was purified by means of column chromatography.

***C**. General procedure for alkyk aziridine:*

Each reaction was performed in a glass vial (10 mL) sealed with an aluminum cap with a rubber septum equipped with a magnetic stirring bar. It was charged with activated Zn^0 dust (40 mg, 0.6 mmol, 3.0 equiv.), DTAC (185 mg, 3.5 equiv.) and B_{12} (2.5 mol%, 6.75 mg). Then water (4.5 mL) and isopropanol (0.5 mL) were added. The resulting mixture was degassed by purging with argon with simultaneous sonication in an ultrasonic bath for 25 min. Then, aziridine (0.2 mmol, 1.0 equiv.) was added dropwise *via* a syringe followed by a Michael acceptor (3.0 equiv.). The resulting mixture was irradiated with green LED light (Kessil, 40 W; λ = 525 nm) for 24 h. at room temperature. The resulting mixture was diluted with AcOEt (~ 3 mL) and washed with brine (20 mL). The organic phase was dried over Na_2SO_4, then filtered through the cotton wool and concentrated *in vacuo*. A crude product was purified by means of column chromatography.

11.1 Note:

- The reaction can be easily monitored by TLC chromatography (AcOEt/Hexane) using UV visualization or the Hanessian's stain.
- Reactions require using activated zinc (unactivated zinc gives a low yield).

11.2 Procedure for the 1 mmol scale synthesis of 5-hydroxy-6-phenoxyhexanenitrile (3)

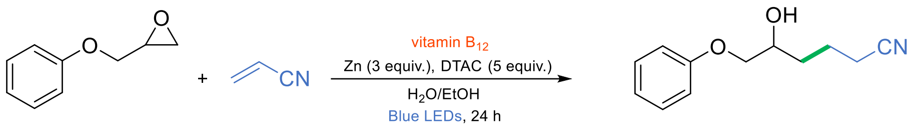

1 2 3

Reaction condition: The reaction was carried out in a 50 mL round-bottom flask sealed with a rubber septum and equipped with a magnetic stirring bar. The flask was charged with activated zinc dust (200 mg, 3 mmol, 3.0 equiv), DTAC (1.32 g, 5.0 equiv), and vitamin B_{12} (67.5 mg, 5.0 mol%). Water (22.5 mL) and ethanol (2.5 mL) were then added. The resulting mixture was degassed by purging with argon while simultaneously sonicating in an ultrasonic bath for 30 minutes. Subsequently, 2-(phenoxymethyl)oxirane **1** (150 mg, 1 mmol, 1.0 equiv) was added dropwise via syringe, followed by acrylonitrile **2** (80 mg, 1.5 mmol, 1.5 equiv). The reaction mixture was irradiated with blue LED light (Kessil lamps, 2 × 20 W, λ = 440 nm) at room temperature for 24 hours. After completion, the mixture was extracted with ethyl AcOEt and brine. The organic layer was dried over anhydrous Na_2SO_4, filtered through cotton wool, and concentrated under reduced pressure. The crude product was purified by flash column chromatography using a hexanes/AcOEt gradient to afford compound **3** (165 mg, 80%) as the final product.

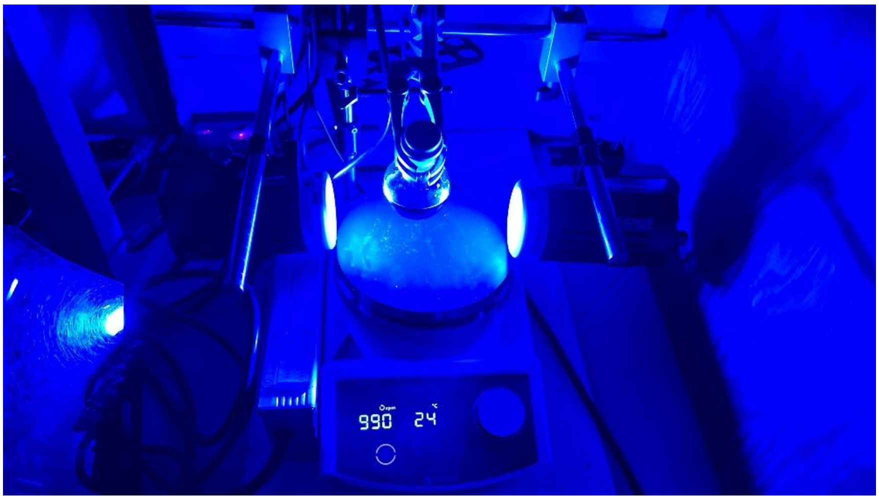

Figure 3. Set-Up for the 1 mmol-scale reaction

12. Scope and characterization of new compounds

12.1. Epoxides

5-hydroxy-6-phenoxyhexanenitrile (3)

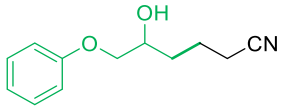

Following the general procedure **A** compound **3** was obtained from 2-(phenoxymethyl)oxirane (**1**) (30 mg, 0.20 mmol) and acrylonitrile (**2**) (16 mg, 0.30 mmol). The crude product was purified by flash chromatography (gradually from hexane to 60:40 ethyl acetate/hexane) to afford 35 mg of 5-hydroxy-6-phenoxyhexanenitrile (**3**) as colorless oil, (yield = **85%**).

^{1}H NMR (400 MHz, $CDCl_3$): δ 7.31 – 7.27 (m, 2H), 6.99 – 6.96 (m, 1H), 6.90 – 6.89 (m, 2H), 4.04 – 4.01 (m, 2H), 3.97 (dd, *J* = 9.2, 3.2 Hz, 1H), 3.84 (dd, *J* = 9.2, 7.3 Hz, 1H), 2.48 – 2.42 (m, 1H), 2.38 (d, *J* = 6 Hz, 1H), 2.00 – 1.91 (m, 1H), 1.87 – 1.78 (m, 1H), 1.77 – 1.66 (m, 2H).

^{13}C NMR (100 MHz, $CDCl_3$): δ 158.3, 129.6, 121.4, 114.5, 77.2, 71.9, 69.3, 31.7, 21.7, 17.1.

HRMS (ESI) $[M+Na]^+$ calculated for $C_{12}H_{15}NO_2Na$: 228.1000, found: 228.1001.

1-phenoxy-5-(phenylsulfonyl)pentan-2-ol (6)

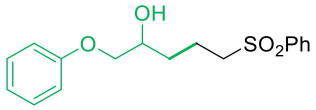

Following the general procedure **A** compound **6** was obtained from 2-(phenoxymethyl)oxirane (30 mg, 0.20 mmol) and phenyl vinyl ketone (**2**) (50 mg, 0.30 mmol). The crude product was purified by flash chromatography (gradually from hexane to 60:40 ethyl acetate/hexane) to afford 21 mg of 1-phenoxy-5-(phenylsulfonyl)pentan-2-ol (**6**) as colorless oil, (yield = **33%**).

^{1}H NMR (500 MHz, $CDCl_3$): δ 7.93 – 7.91 (m, 2H), 7.67 – 7.7.64 (m, 1H), 7.58 – 7.55 (m, 2H), 7.30 – 7.26 (m, 2H), 6.98 – 6.95 (m, 1H), 6.88 – 6.86 (m, 2H), 3.98 – 3.94 (m, 1H), 3.92 (dd, *J* = 9.2, 3.4 Hz, 1H), 3.80 (dd, *J* = 9.2, 7.2 Hz, 1H), 3.25 – 3.14 (m, 2H), 2.38 (s, 1H), 2.04 – 1.95 (m, 1H), 1.93 – 1.86 (m, 1H), 1.70 – 1.60 (m, 2H).

^{13}C NMR (125 MHz, $CDCl_3$): δ 158.3, 139.1, 133.7, 129.6, 129.3, 128.1, 121.3, 114.5, 71.8, 69.5, 56.0, 31.4, 19.2.

HRMS (ESI) $[M+Na]^+$ calculated for $C_{17}H_{20}O_4SNa$: 343.0980, found: 343.0983.

5-hydroxy-5-phenylpentanenitrile (7)

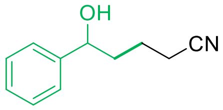

Following the general procedure **B** compound **7** was obtained from 2-phenyloxirane (24 mg, 0.20 mmol) and acrylonitrile (**2**) (16 mg, 0.30 mmol). The crude product was purified by flash chromatography (gradually from hexane to 60:40 ethyl acetate/hexane) to afford 17 mg of 5-hydroxy-5-phenylpentanenitrile (**7**) as colorless oil, (yield = **48%**).

NMR data matched those reported in the literature.[9]

^{1}H NMR (500 MHz, $CDCl_3$): δ 7.40 – 7.28 (m, 5H), 4.76 – 4.72 (m, 1H), 2.44 – 2.32 (m, 2H), 1.98 – 1.79 (m, 4H).

5-(4-fluorophenyl)-5-hydroxypentanenitrile (8)

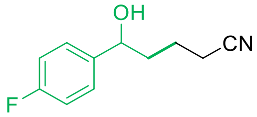

Following the general procedure **B** compound **8** was obtained from 2-(4-fluorophenyl)oxirane (28 mg, 0.20 mmol) and acrylonitrile (**2**) (16 mg, 0.30 mmol). The crude product was purified by flash chromatography (gradually from hexane to 60:40 ethyl acetate/hexane) to afford 20 mg of 5-(4-fluorophenyl)-5-hydroxypentanenitrile (**8**) as colorless oil, (yield = **53%**).

^{1}H NMR (500 MHz, $CDCl_3$): δ 7.34 – 7.29 (dd, *J* = 8.35, 5.49 Hz, 2H), 7.07 – 7.02 (t, *J* = 8.60 Hz, 2H), 4.76 – 4.69 (dd, *J* = 7.31, 4.43 Hz, 1H), 2.46 – 2.42 (m, 1H), 2.42 – 2.35 (q, *J* = 6.68 Hz, 2H), 1.92 – 1.81 (m, 4H).

^{13}C NMR (151 MHz, $CDCl_3$): δ 163.12, 161.49, 139.73, 127.37, 119.49, 118.59, 115.55, 115.41, 73.04, 37.68, 24.26, 21.79, 17.07, 16.68.

HRMS (APCI) $[M+H]^+$ calculated for $C_{11}H_{13}NOF$: 194.0981, found: 194.0982.

5-hydroxynonanenitrile (9)

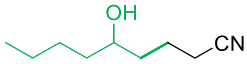

Following the general procedure **A** compound **9** was obtained from 2-butyloxirane (20 mg, 0.20 mmol) and acrylonitrile (**2**) (16 mg, 0.30 mmol). The crude product was purified by flash chromatography (gradually from hexane to 60:40 ethyl acetate/hexane) to afford 23 mg of 5-hydroxynonanenitrile (**9**) as colorless oil, (yield = **73%**).

NMR data matched those reported in the literature.[10]

^{1}H NMR (500 MHz, $CDCl_3$): δ 3.67 – 3.60 (m, 1H), 2.44 – 2.37 (m, 2H), 1.91 – 1.82 (m, 1H), 1.79 – 1.70 (m, 1H), 1.69 – 1.61 (m, 1H), 1.55 – 1.43 (m, 3H), 1.42 – 1.28 (m, 5H), 0.91 (t, *J* = 7.0 Hz, 3H).

^{13}C NMR (125 MHz, $CDCl_3$): δ 119.7, 71.1, 37.5, 35.9, 27.7, 22.6, 21.8, 17.2, 14.0.

[9] Nagaki, A.; Yamashita, H.; Hirose, K.; Tsuchihashi, Y.; Yoshida, J. Alkyllithium Compounds Bearing Electrophilic Functional Groups: A Flash Chemistry Approach. *Angew. Chem. Int. Ed.* **2019**, *58*, 4027-4030. https://doi.org/10.1002/anie.201814088.
[10] O'Shea, M. G.; Kitching, W. Organotin and -Mercury Routes to Enones, Dienones Amd Spiroacetals. *Tetrahedron* **1989**, *45* (4), 1177–1186. https://doi.org/https://doi.org/10.1016/0040-4020(89)80026-2.

5-hydroxypentadecanenitrile (10)

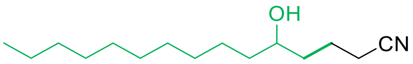

Following the general procedure **A** compound **10** was obtained from 1,2-epoxydodecane (37 mg, 0.20 mmol) and acrylonitrile (**2**) (16 mg, 0.30 mmol). The crude product was purified by flash chromatography (gradually from hexane to 60:40 ethyl acetate/hexane) to afford 19 mg of 5-hydroxypentadecanenitrile (**10**) as colorless oil, (yield = **40%**).

^{1}H NMR (500 MHz, $CDCl_3$): δ 3.65 – 3.60 (m, 1H), 2.39 (t, *J* = 7.2 Hz, 2H), 1.90 – 1.81 (m, 1H), 1.77 – 1.69 (m, 1H), 1.67 – 1.59 (m, 1H), 1.55 – 1.48 (m, 1H), 1.47 – 1.36 (m, 4H), 1.26 (m, 15H), 0.87 (t, *J* = 6.9 Hz, 3H).

^{13}C NMR (125 MHz, $CDCl_3$): δ 119.7, 71.1, 37.8, 35.9, 31.9, 29.6, 29.6, 29.3, 25.6, 22.66, 21.8, 17.2, 14.1.

HRMS (APCI) $[M+H]^+$ calculated for $C_{15}H_{30}NO$: 240.2327, found: 240.2328.

5-hydroxy-6-(naphthalen-2-yloxy)hexanenitrile (11)

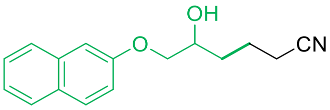

Following the general procedure **A** compound **11** was obtained from 2-((naphthalen-2-yloxy)methyl)oxirane (40 mg, 0.20 mmol) and acrylonitrile (**2**) (16 mg, 0.30 mmol). The crude product was purified by flash chromatography (gradually from hexane to 50:50 ethyl acetate/hexane) to afford 21 mg of 5-hydroxy-6-(naphthalen-2-yloxy)hexanenitrile (**11**) as colorless oil, (yield = **41%**).

^{1}H NMR (500 MHz, $CDCl_3$): δ 7.79 – 7.72 (m, 3H), 7.48 – 7.43 (m, 1H), 7.38 – 7.34 (m, 1H), 7.17 – 7.14 (m, 2H), 4.14 – 4.07 (m, 2H), 3.97 (dd, *J* = 10.0, 8.5 Hz, 1H), 2.53 – 2.40 (m, 3H), 2.00 – 1.95 (m, 1H), 1.90 – 1.85 (m, 1H), 1.82 – 1.70 (m, 2H).

^{13}C NMR (125 MHz, $CDCl_3$): δ 156.3, 134.4, 129.6, 129.2, 127.7, 126.8, 126.6, 124.0, 119.5, 118.5, 107.0, 72.0, 69.3, 31.8, 21.8, 17.2.

HRMS (ESI) $[M+Na]^+$ calculated for $C_{16}H_{17}NO_2Na$: 278.1157, found: 278.1158.

***tert*-butyl ((2S)-6-cyano-3-hydroxy-1-phenylhexan-2-yl)carbamate (12)**

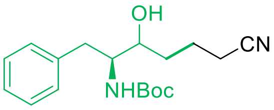

Following the general procedure **A** compound **12** was obtained from tert-butyl ((1*S*)-1-(oxiran-2-yl)-2-phenylethyl)carbamate (53 mg, 0.20 mmol) and acrylonitrile (**2**) (16 mg, 0.30 mmol). The crude product was purified by flash chromatography (gradually from hexane to 60:40 ethyl acetate/hexane) to afford 26 mg of tert-butyl ((2S)-6-cyano-3-hydroxy-1-phenylhexan-2-yl)carbamate (**12**) as a white solid, (yield = **40%**).

^{1}H NMR (500 MHz, $CDCl_3$): δ 7.32 – 7.29 (m, 2H), 7.24 – 7.22 (m, 3H), 4.58 (d, *J* = 7.4 Hz, 1H), 3.83 (br s, 1H), 3.69 – 3.68 (m, 1H), 3.32 (br s, 1H), 2.86 (dd, *J* = 14.1, 5.0 Hz, 1H), 2.82 – 2.71 (m, 1H), 2.41 (td, *J* = 7.0, 2.4 Hz, 2H), 1.98 – 1.92 (m, 1H), 1.78 – 1.73 (m, 1H), 1.61 – 1.57 (m, 2H), 1.37 (s, 9H).

^{13}C NMR (125 MHz, $CDCl_3$): δ 137.6, 129.1, 128.7, 126.7, 119.7, 80.2, 73.2, 57.2, 36.0, 31.5, 28.2, 22.1, 17.1.

HRMS (ESI) $[M+Na]^+$ calculated for $C_{18}H_{26}N_2O_3Na$: 341.1841, found: 341.1844.

benzyl (5-cyano-2-hydroxypentyl)carbamate (13)

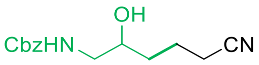

Following the general procedure **A** compound **13** was obtained from benzyl (oxiran-2-ylmethyl)carbamate (**S1**) (38 mg, 0.20 mmol) and acrylonitrile (**2**) (16 mg, 0.30 mmol). The crude product was purified by flash chromatography (gradually from hexane to 80:20 ethyl acetate/hexane) to afford 26 mg of benzyl (5-cyano-2-hydroxypentyl)carbamate (**13**) as colorless oil, (yield = **53%**).

^{1}H NMR (500 MHz, CDCl$_3$): δ 7.38 – 7.32 (m, 5H), 5.22 (br s, 1H), 5.10 (s, 2H), 3.73 (s, 1H), 3.36 – 3.29 (m, 1H), 3.17 – 3.07 (m, 1H), 2.71 (s, 1H), 2.39 – 2.36 (m, 2H), 1.89 – 1.79 (m, 1H), 1.77 – 1.71 (m, 1H), 1.56 – 1.55 (m, 1H), NH was not detected in the NMR spectrum, likely due to solvent-mediated exchange.

^{13}C NMR (125 MHz, CDCl$_3$): δ 157.4, 136.2, 128.6, 128.3, 128.1, 119.6, 70.6, 67.1, 47.1, 33.2, 21.7, 17.1.

HRMS (APCI) $[M+H]^+$ calculated for $C_{14}H_{19}N_2O_3$: 263.1396, found: 263.1398.

5-hydroxy-7-(phenylsulfonyl)heptanenitrile (14)

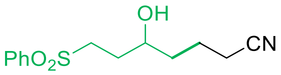

Following the general procedure **A** compound **14** was obtained from 2-(2-(phenylsulfonyl)ethyl)oxirane (42 mg, 0.20 mmol) and acrylonitrile (**2**) (16 mg, 0.30 mmol). The crude product was purified by flash chromatography (gradually from hexane to 80:20 ethyl acetate/hexane) to afford 34 mg of 5-hydroxy-7-(phenylsulfonyl)heptanenitrile (**14**) as a white solid, (yield = **63%**).

^{1}H NMR (500 MHz, CDCl$_3$): δ 7.94 – 7.89 (m, 2H), 7.69 – 7.66 (m, 1H), 7.60 – 7.57 (m, 2H), 3.84 – 3.75 (m, 1H), 3.31 – 3.22 (m, 2H), 2.39 (t, *J* = 7.0 Hz, 2H), 2.10 (d, *J* = 5.2 Hz, 1H), 2.01 – 1.92 (m, 1H), 1.89 – 1.77 (m, 2H), 1.77 – 1.67 (m, 1H), 1.64 – 1.50 (m, 2H).

^{13}C NMR (125 MHz, CDCl$_3$): δ 139.0, 133.9, 129.4, 127.9, 119.5, 69.0, 52.9, 36.0, 30.2, 21.7, 17.1.

HRMS (ESI) $[M+Na]^+$ calculated for $C_{13}H_{17}NO_3SNa$: 290.0827, found: 290.0826.

12.2. Aziridines

N-(1-cyanooctan-4-yl)-4-methylbenzenesulfonamide (5)

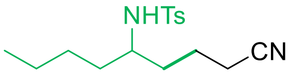

Following the general procedure **C** compound **5** was obtained from 2-butyl-1-tosylaziridine (**4**) (51 mg, 0.20 mmol) and acrylonitrile (**2**) (16 mg, 0.30 mmol). The crude product was purified by flash chromatography (gradually from hexane to 60:40 ethyl acetate/hexane) to afford 50 mg of (**5**) as *N*-(1-cyanooctan-4-yl)-4-methylbenzenesulfonamide colorless oil, (yield = **80%**).

^{1}H NMR (400 MHz, $CDCl_3$): δ = 7.74 (d, *J* = 8.3 Hz, 2H), 7.30 – 7.29 (m, 2H), 4.47 (d, *J* = 8.6 Hz, 1H), 3.23 – 3.17 (m, 1H), 2.42 (s, 3H), 2.29 (td, *J* = 6.7, 2.4 Hz, 2H), 1.74 – 1.65 (m, 1H), 1.62 (m, 2H), 1.47 – 1.39 (m, 1H), 1.34 – 1.27 (m, 1H), 1.26 – 1.20 (m, 1H), 1.15 – 1.04 (m, 3H), 1.04 – 0.96 (m, 1H), 0.73 (t, *J* = 7.1 Hz, 3H).

^{13}C NMR (100 MHz, $CDCl_3$): δ = 143.5, 138.1, 129.7, 126.9, 119.4, 53.3, 35.0, 34.1, 27.4, 22.3, 21.5, 21.4, 16.8, 13.7.

HRMS (ESI) $[M+Na]^+$ calculated for $C_{16}H_{24}N_2O_2SNa$: 331.1456, found: 331.1458.

4-methyl-*N*-(9-oxodecan-5-yl)benzenesulfonamide (15)

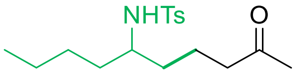

Following the general procedure **C** compound **15** was obtained from 2-butyl-1-tosylaziridine (**4**) (51 mg, 0.20 mmol) and methyl vinyl ketone (21 mg, 0.30 mmol). The crude product was purified by flash chromatography (gradually from hexane to 60:40 ethyl acetate/hexane) to afford 26 mg of (**15**) as 4-methyl-*N*-(9-oxodecan-5-yl)benzenesulfonamide colorless oil, (yield = **40%**).

^{1}H NMR (400 MHz, $CDCl_3$): δ = 7.75 (d, *J* = 8.2 Hz, 2H), 7.29 (d, *J* = 8.0 Hz, 2H), 4.35 (d, *J* = 8.3 Hz, 1H), 3.18 (h, *J* = 6.8 Hz, 1H), 2.42 (s, 3H), 2.32 (t, *J* = 7.0 Hz, 2H), 2.08 (s, 3H), 1.52 – 1.36 (m, 3H), 1.36 – 1.24 (m, 3H), 1.16-1.09 (m, 3H), 1.07 – 1.01 (m, 1H), 0.76 (t, *J* = 7.0 Hz, 3H).

^{13}C NMR (100 MHz, $CDCl_3$): δ = 208.5, 143.2, 138.3, 129.6, 127.0, 53.8, 43.1, 34.6, 34.4, 29.8, 27.4, 22.4, 21.5, 19.3, 13.8.

HRMS (APCI) $[M–H]^-$ calculated for $C_{17}H_{26}N_2O_3S$: 324.1633, found: 324.1636.

4-methyl-*N*-(1-(phenylsulfonyl)octan-4-yl)benzenesulfonamide (16)

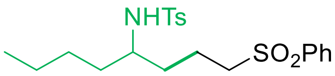

Following the general procedure **C** compound **16** was obtained from 2-butyl-1-tosylaziridine (**4**) (51 mg, 0.20 mmol) and phenyl vinyl ketone (50 mg, 0.30 mmol). The crude product was purified by flash chromatography (gradually from hexane to 60:40 ethyl acetate/hexane) to afford 14 mg of (**16**) as 4-methyl-*N*-(1-(phenylsulfonyl)octan-4-yl)benzenesulfonamide colorless oil, (yield = **17%**).

^{1}H NMR (400 MHz, CDCl$_3$): δ = 7.89 (d, *J* = 7.2 Hz, 2H), 7.71 (d, *J* = 8.2 Hz, 2H), 7.67 (t, *J* = 7.4 Hz, 1H), 7.58 (t, *J* = 7.7 Hz, 2H), 7.29 (d, *J* = 8.0 Hz, 2H), 4.27 (d, *J* = 8.4 Hz, 1H), 3.21 – 3.12 (m, 1H), 3.02 (pt, *J* = 7.9, 4.4 Hz, 2H), 2.42 (s, 3H), 1.80 – 1.64 (m, 2H), 1.49 – 1.39 (m, 1H), 1.34 – 1.16 (m, 3H), 1.14 – 1.01 (m, 3H), 1.01 – 0.92 (m, 1H), 0.73 (t, *J* = 7.1 Hz, 3H).

^{13}C NMR (100 MHz, CDCl$_3$): δ = 143.4, 139.1, 138.0, 133.7, 129.7, 129.3, 128.0, 127.0, 55.7, 53.4, 34.6, 33.7, 27.3, 22.3, 21.5, 18.7, 13.7.

HRMS (ESI) [M+Na]$^+$ calculated for $C_{21}H_{29}NO_4S_2Na$: 446.1436, found: 446.1437.

***N*-(1-cyanotetradecan-4-yl)-4-methylbenzenesulfonamide (17)**

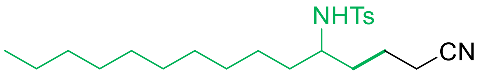

Following the general procedure **C** compound **17** was obtained from 2-decyl-1-tosylaziridine (**S3**) (68 mg, 0.20 mmol) and acrylonitrile (**2**) (16 mg, 0.30 mmol). The crude product was purified by flash chromatography (gradually from hexane to 60:40 ethyl acetate/hexane) to afford 37 mg of (**17**) as *N*-(1-cyanotetradecan-4-yl)-4-methylbenzenesulfonamide colorless oil, (yield = **47%**).

^{1}H NMR (400 MHz, CDCl$_3$): δ = 7.74 (d, *J* = 8.1 Hz, 2H), 7.31 (d, *J* = 8.1 Hz, 2H), 4.34 (d, *J* = 8.6 Hz, 1H), 3.27 – 3.16 (m, 1H), 2.43 (s, 3H), 2.31 (dt, *J* = 6.9, 3.9 Hz, 2H), 1.75 – 1.60 (m, 3H), 1.49 – 1.41 (m, 1H), 1.34 – 1.13 (m, 14H), 1.10 – 1.04 (m, 4H), 0.88 (t, *J* = 7.0 Hz, 3H).

^{13}C NMR (100 MHz, CDCl$_3$): δ = 143.5, 138.1, 129.7, 127.0, 119.4, 53.3, 35.3, 34.2, 31.9, 29.6, 29.5, 29.3, 29.3, 29.2, 25.3, 22.7, 21.5, 21.4, 16.8, 14.1.

HRMS (ESI) [M+Na]$^+$ calculated for $C_{22}H_{36}N_2O_2SNa$: 415.2395, found: 415.2399.

4-methyl-*N*-(2-oxohexadecan-6-yl)benzenesulfonamide (18)

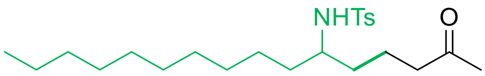

Following the general procedure **C** compound **18** was obtained from 2-decyl-1-tosylaziridine (**S3**) (68 mg, 0.20 mmol) and methyl vinyl ketone (21 mg, 0.30 mmol). The crude product was purified by flash chromatography (gradually from hexane to 60:40 ethyl acetate/hexane) to afford 33 mg of (**18**) as 4-methyl-*N*-(2-oxohexadecan-6-yl)benzenesulfonamide colorless oil, (yield = **40%**).

^{1}H NMR (400 MHz, $CDCl_3$): δ = 7.74 (d, *J* = 8.2 Hz, 2H), 7.29 (d, *J* = 8.0 Hz, 2H), 4.28 (d, *J* = 8.3 Hz, 1H), 3.18 (h, *J* = 6.7 Hz, 1H), 2.42 (s, 3H), 2.33 (t, *J* = 7.0 Hz, 2H), 2.09 (s, 3H), 1.52 – 1.38 (m, 3H), 1.37 – 1.14 (m, 15H), 1.12 – 1.09 (m, 4H), 0.88 (t, *J* = 7.0 Hz, 3H).

^{13}C NMR (100 MHz, $CDCl_3$): δ = 208.5, 143.2, 138.3, 129.6, 127.0, 53.9, 43.1, 34.9, 34.5, 31.9, 29.8, 29.6, 29.5, 29.4, 29.3, 29.3, 25.2, 22.7, 21.5, 19.3, 14.1.

HRMS (ESI) $[M-Na]^+$ calculated for $C_{23}H_{39}NO_3SNa$: 432.2548, found: 432.2553.

***N*-(cyclopent-2-en-1-yl)-4-methylbenzenesulfonamide (19)**

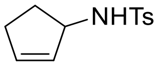

Following the general procedure **C** side product **19** was obtained from 6-tosyl-6-azabicyclo[3.1.0]hexane (**S7**) (48 mg, 0.20 mmol) and acrylonitrile (**2**) (16 mg, 0.30 mmol). The crude product was purified by flash chromatography (gradually from hexane to 60:40 ethyl acetate/hexane) to afford 29 mg of (**19**) as *N*-(cyclopent-2-en-1-yl)-4-methylbenzenesulfonamide white solid, (yield = **60%**).

NMR data matched those reported in the literature.[11]

^{1}H NMR (400 MHz, $CDCl_3$): δ = 7.77 (d, *J* = 8.3 Hz, 2H), 7.30 (d, *J* = 8.1 Hz, 2H), 5.89 – 5.81 (m, 1H), 5.44 (dt, *J* = 5.5, 2.0 Hz, 1H), 4.48 – 4.33 (m, 2H), 2.43 (s, 3H), 2.35 (m, 1H), 2.24 – 2.06 (m, 2H), 1.55 – 1.45 (m, 1H).

[11] Yamamoto, H.; Ho, E.; Sasaki, I.; Mitsutake, M.; Takagi, Y.; Imagawa, H.; Nishizawa, M. Intermolecular Amination of Allyl Alcohols with Sulfamates: Effective Utilization of Mercuric Catalyst. *European J. Org. Chem.* **2011**, *2011* (13), 2417–2420. https://doi.org/https://doi.org/10.1002/ejoc.201100054.

N-(cyclohex-2-en-1-yl)-4-methylbenzenesulfonamide (20)

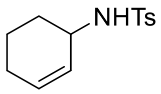

Following the general procedure **C** side product **20** was obtained from 7-tosyl-7-azabicyclo[4.1.0]heptane (**S8**) (50 mg, 0.20 mmol) and acrylonitrile (**2**) (16 mg, 0.30 mmol). The crude product was purified by flash chromatography (gradually from hexane to 60:40 ethyl acetate/hexane) to afford 35 mg of (**20**) as *N*-(cyclohex-2-en-1-yl)-4-methylbenzenesulfonamide white solid, (yield = **70%**).

NMR data matched those reported in the literature.[12]

^{1}H NMR (400 MHz, $CDCl_3$): δ = 7.77 (d, *J* = 8.3 Hz, 2H), 7.29 (d, *J* = 8.1 Hz, 2H), 5.79 – 5.74 (m, 1H), 5.37 – 5.32 (m, 1H), 4.58 (d, *J* = 8.5 Hz, 1H), 3.84 – 3.79 (m, 1H), 2.42 (s, 3H), 1.98 – 1.86 (m, 2H), 1.75 (m, 1H), 1.62 – 1.48 (m, 3H + H_2O).

[12] Wallach, D. R.; Chisholm, J. D. Alkylation of Sulfonamides with Trichloroacetimidates under Thermal Conditions. *J. Org. Chem.* **2016**, *81* (17), 8035–8042. https://doi.org/10.1021/acs.joc.6b01421.

13. NMR Spectra

5-hydroxy-6-phenoxyhexanenitrile (3)

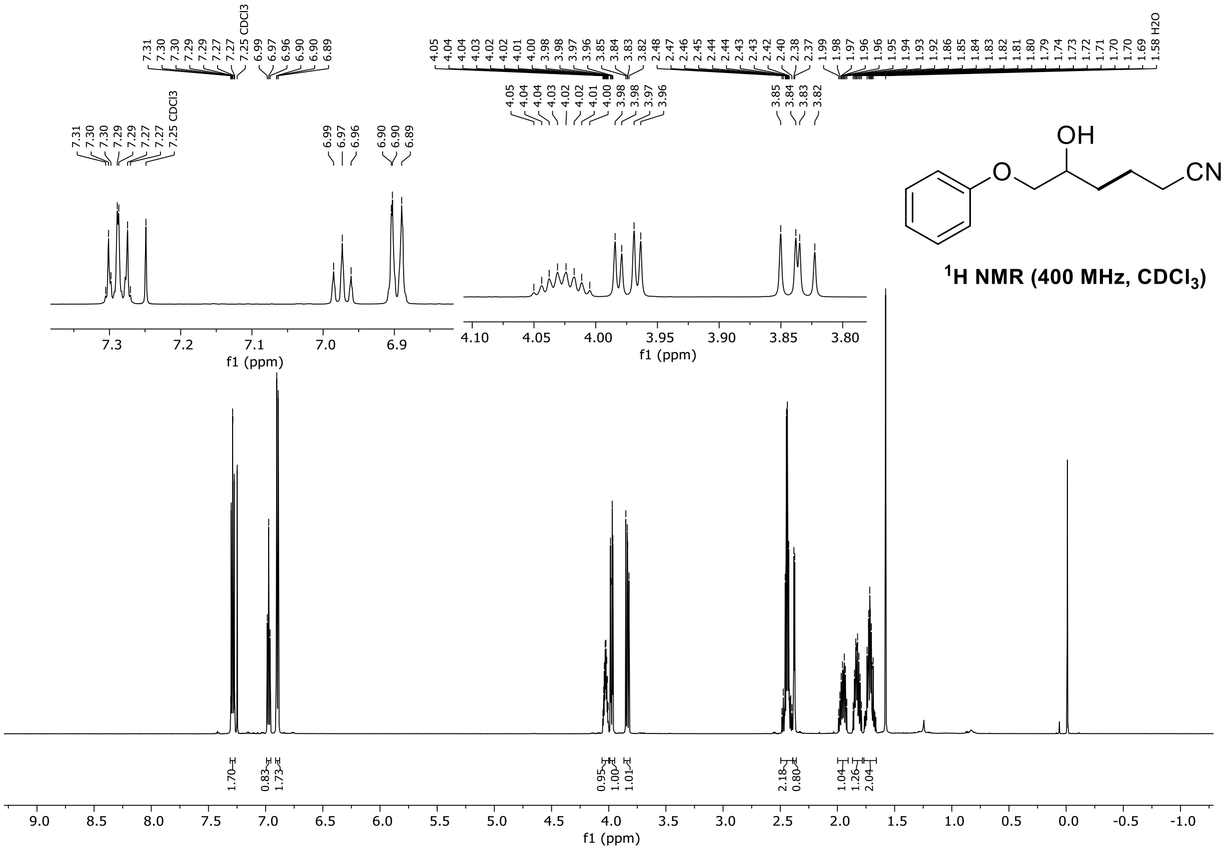

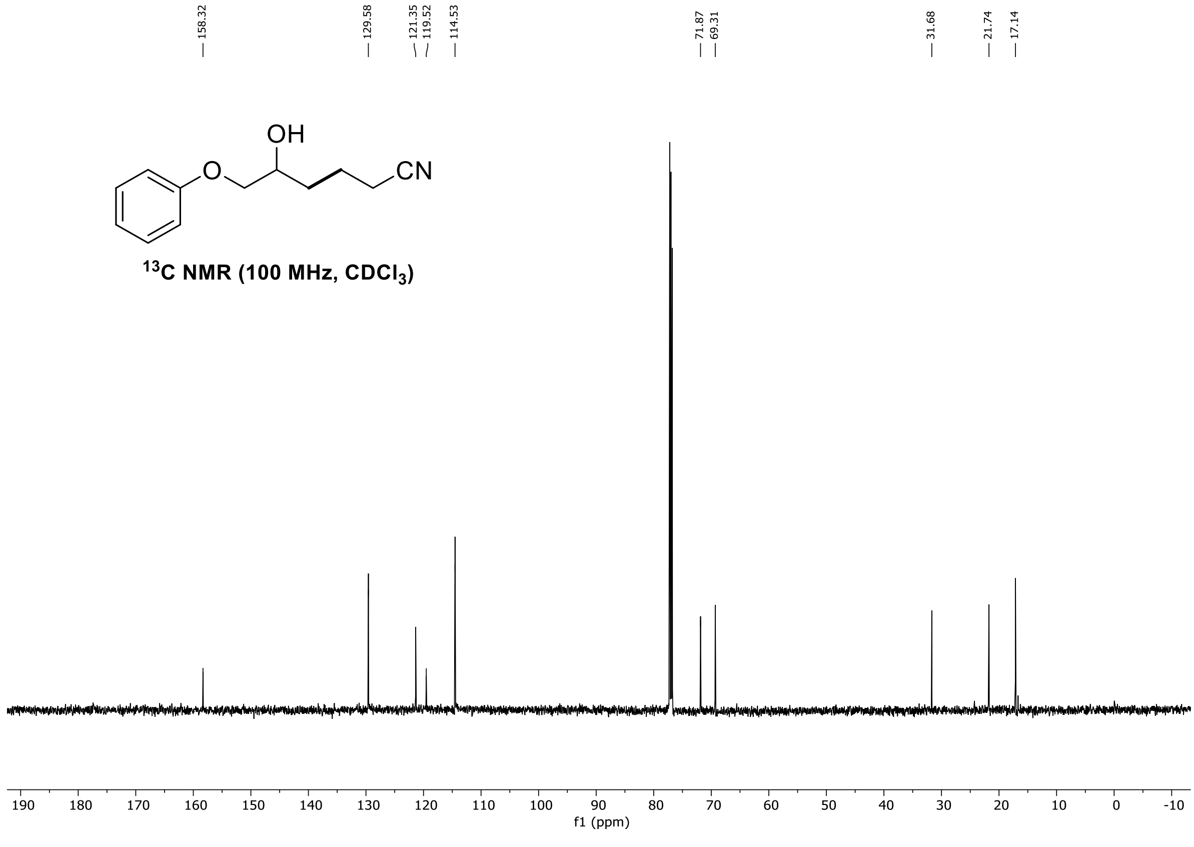

1-phenoxy-5-(phenylsulfonyl)pentan-2-ol (**6**)

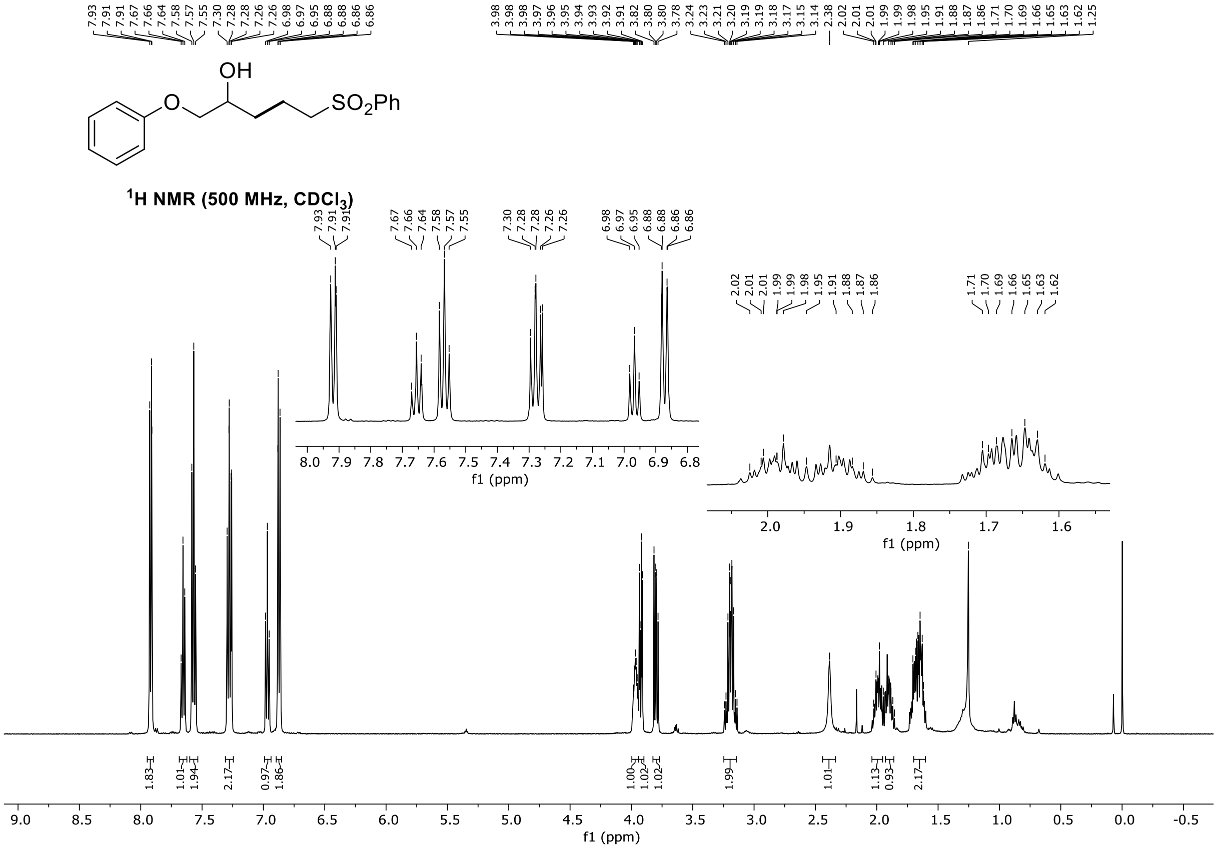

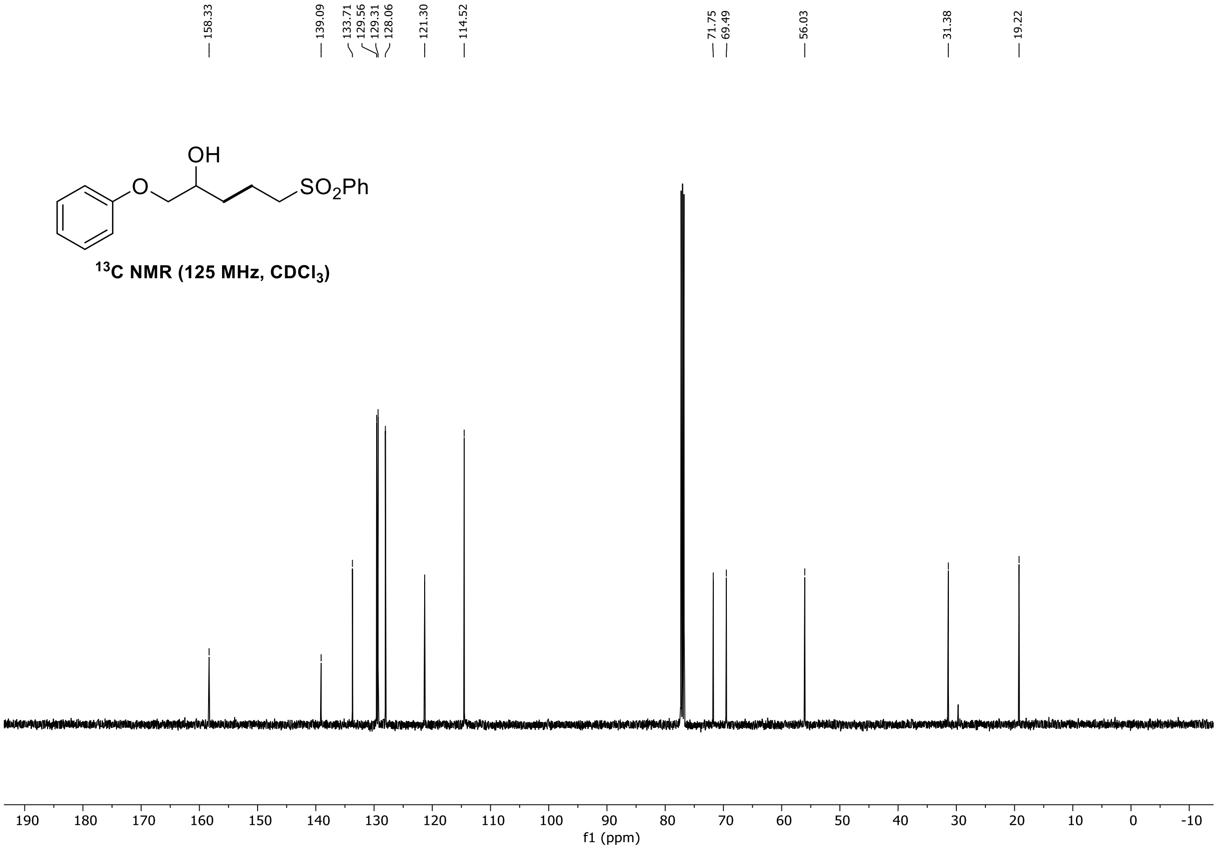

5-hydroxy-5-phenylpentanenitrile (**7**)

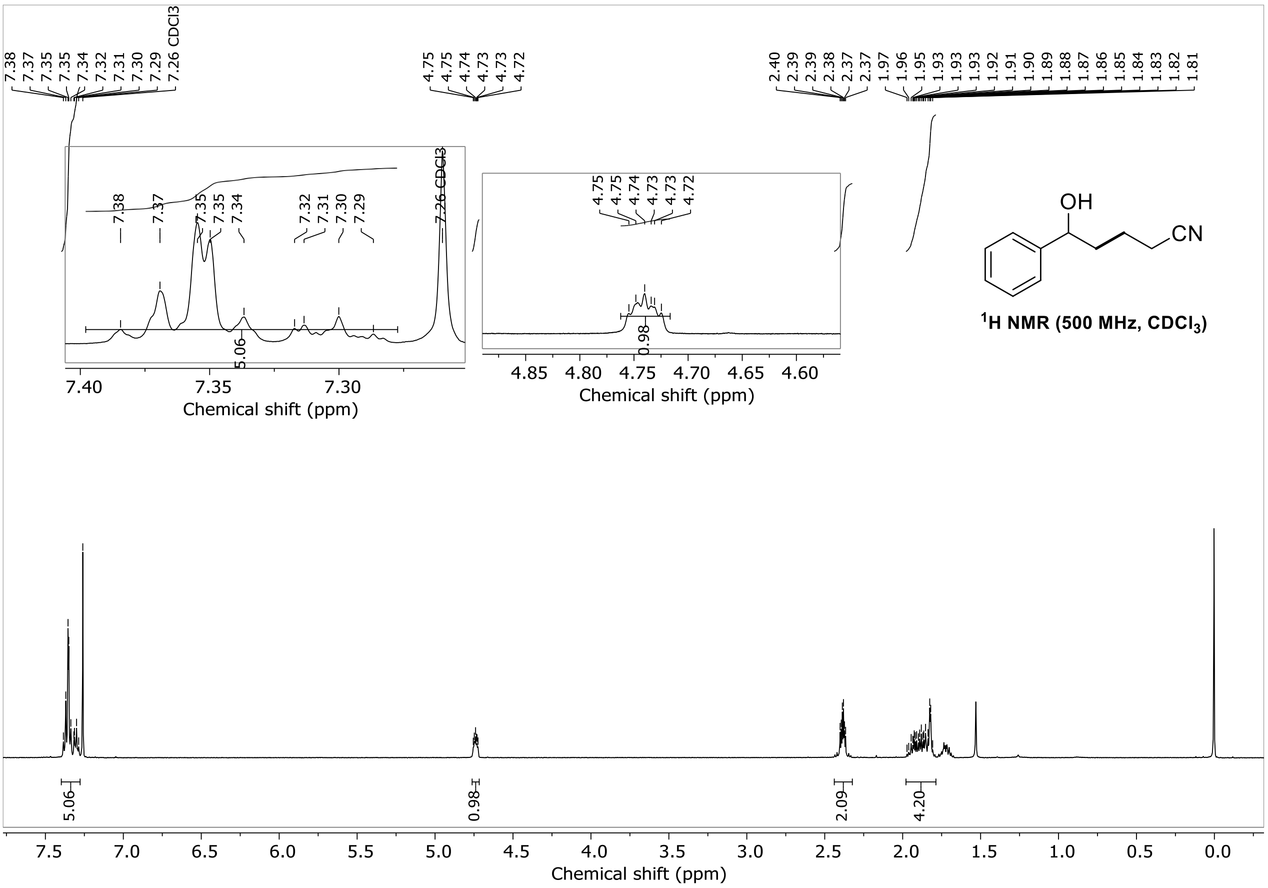

5-(4-fluorophenyl)-5-hydroxypentanenitrile (**8**)

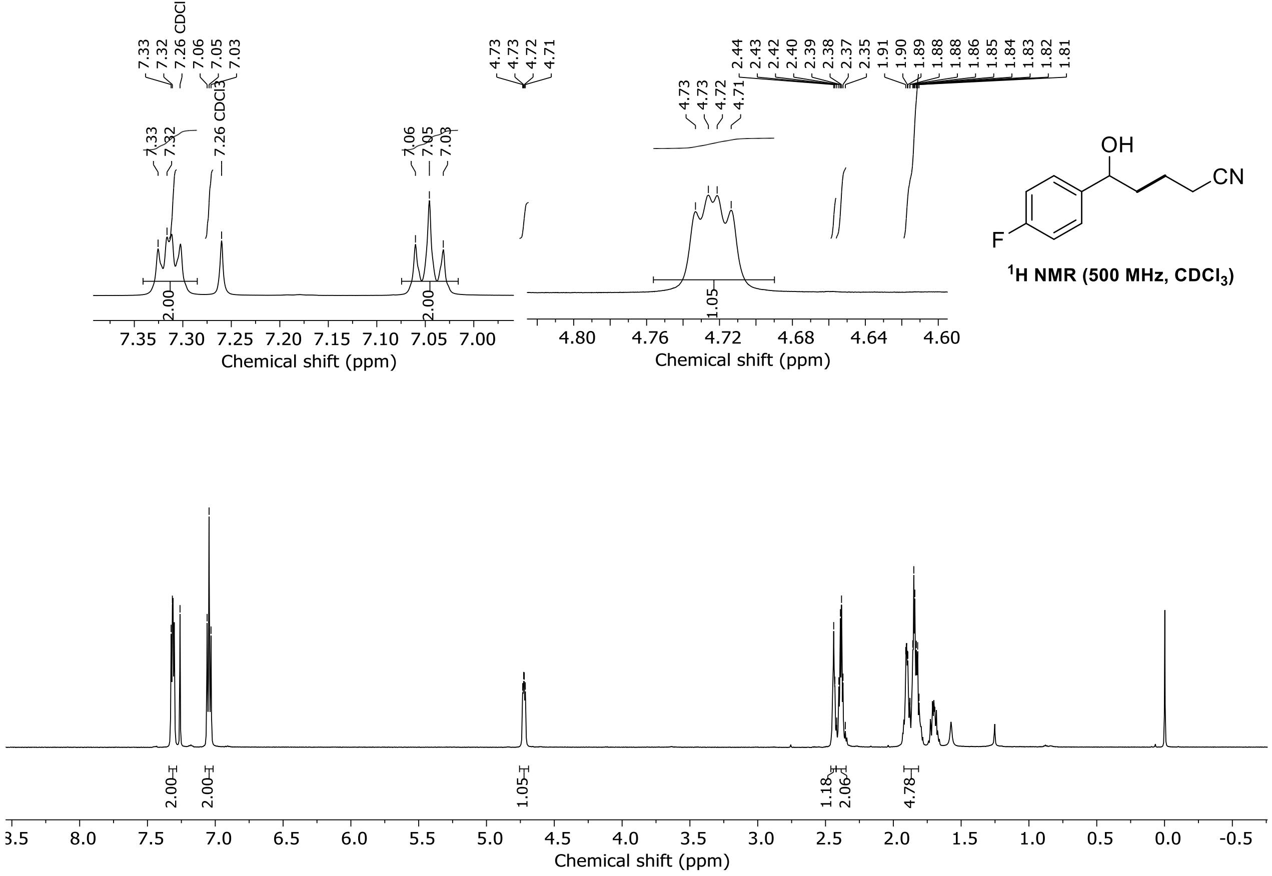

^{1}H NMR (500 MHz, $CDCl_3$)

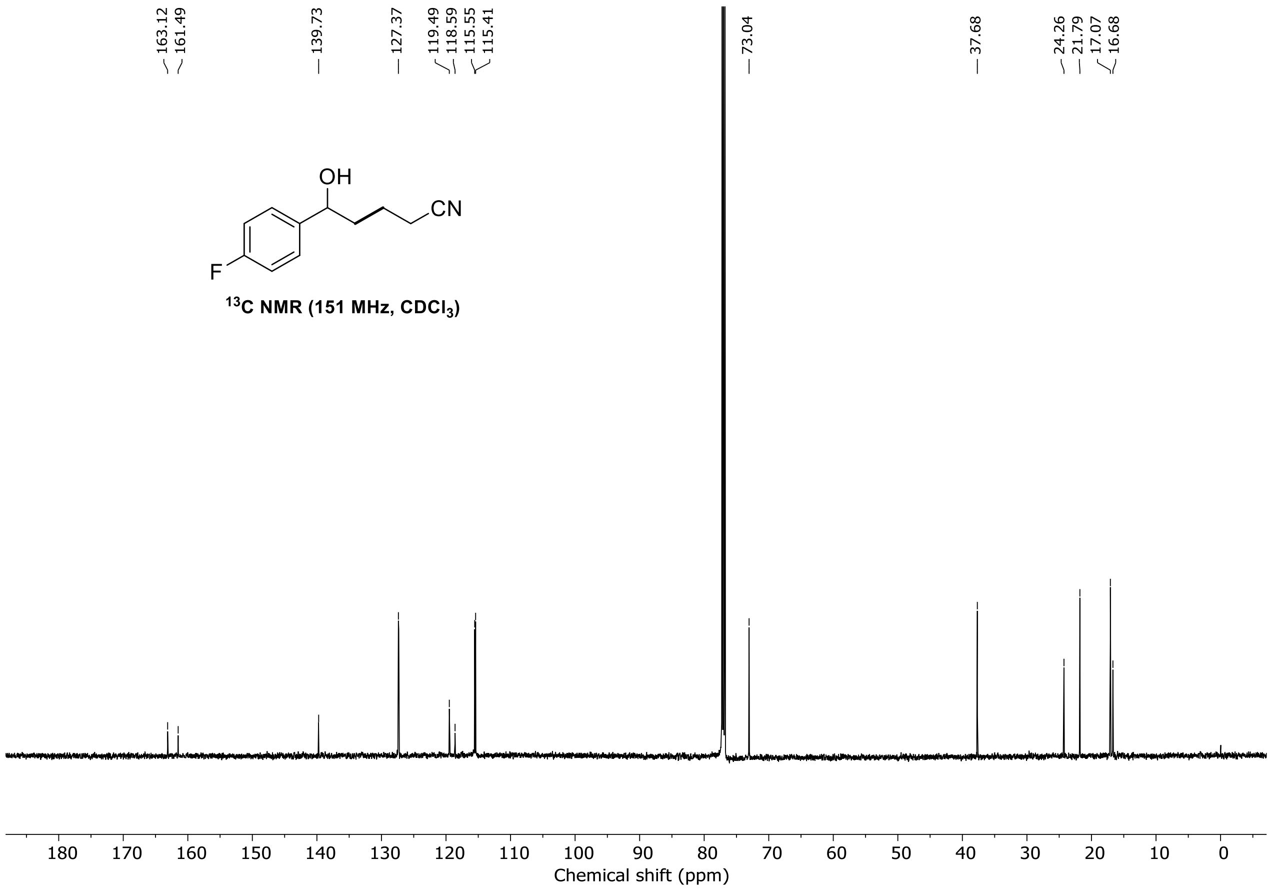

^{13}C NMR (151 MHz, $CDCl_3$)

5-hydroxynonanenitrile (**9**)

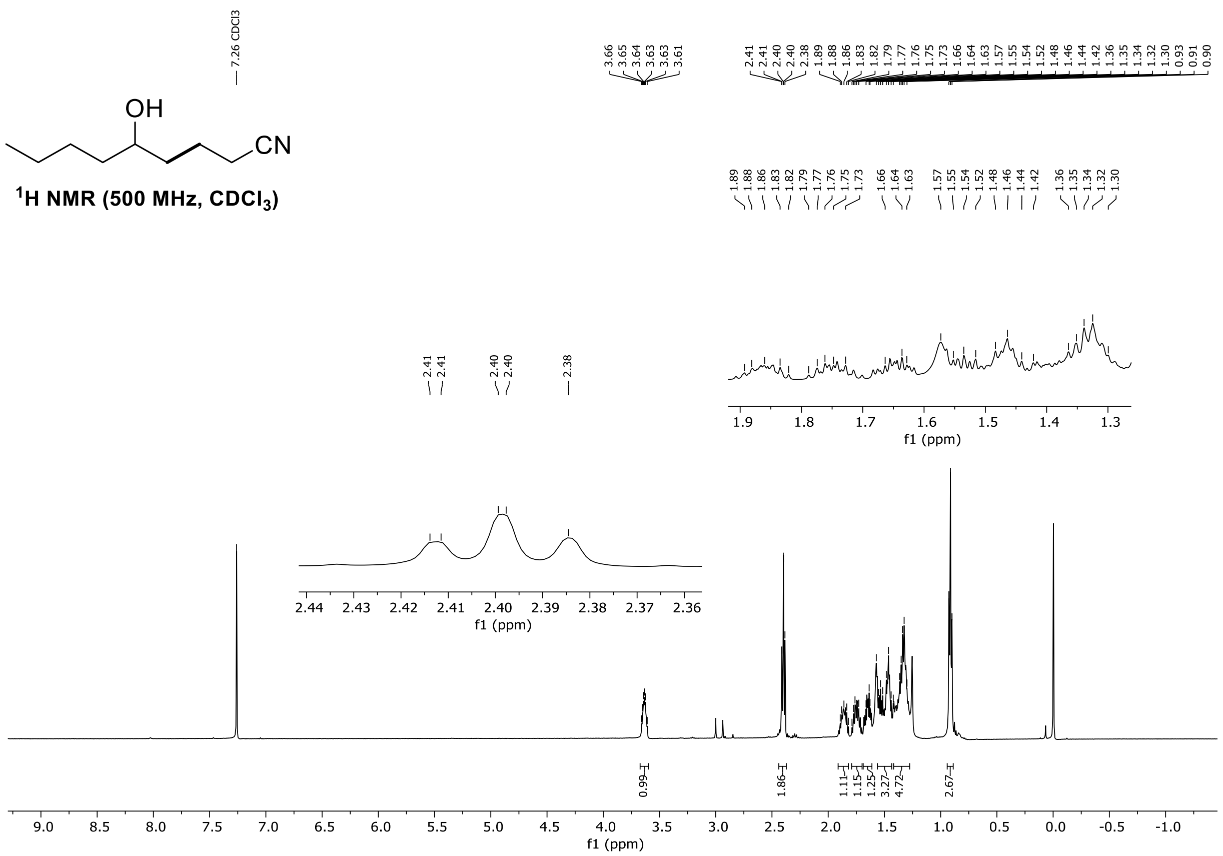

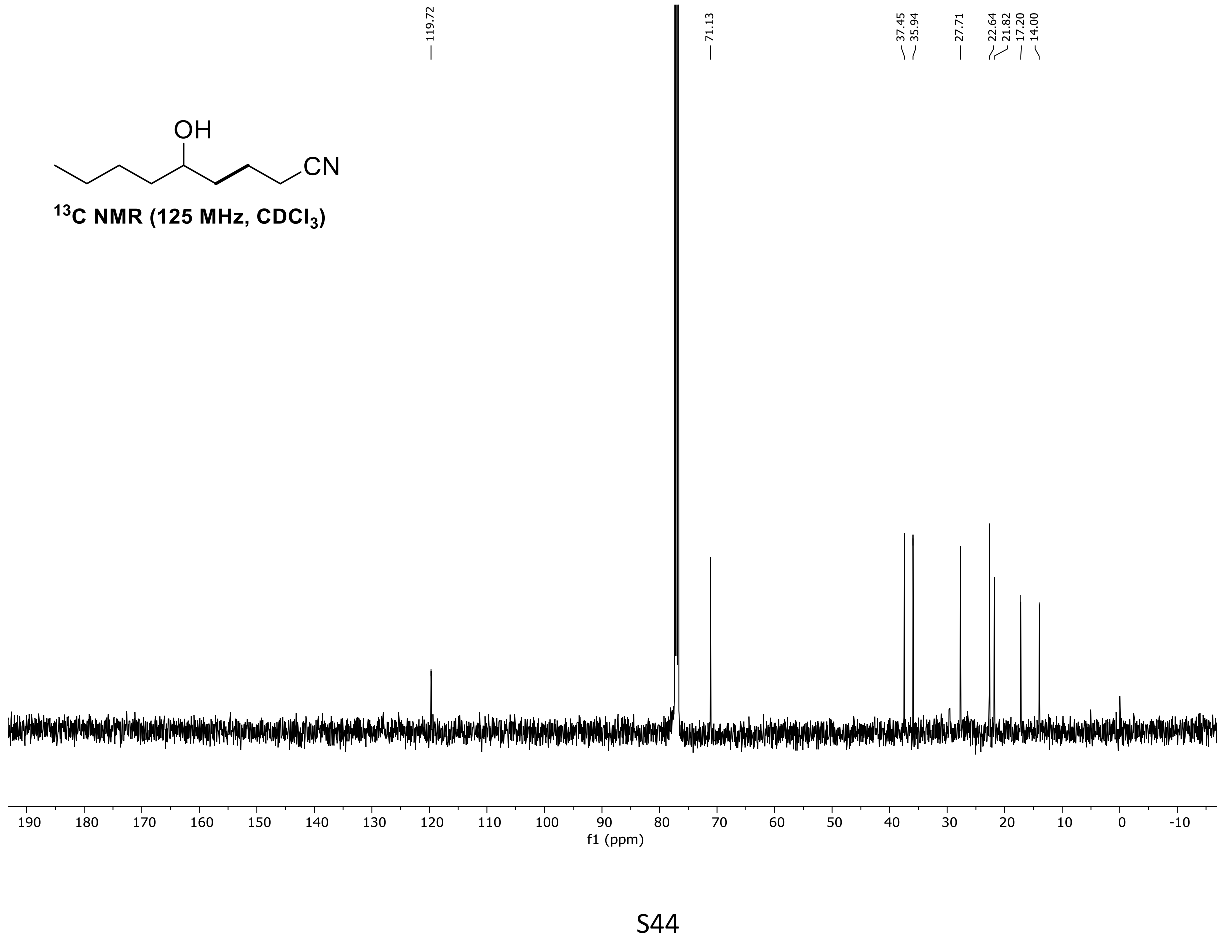

5-hydroxypentadecanenitrile (**10**)

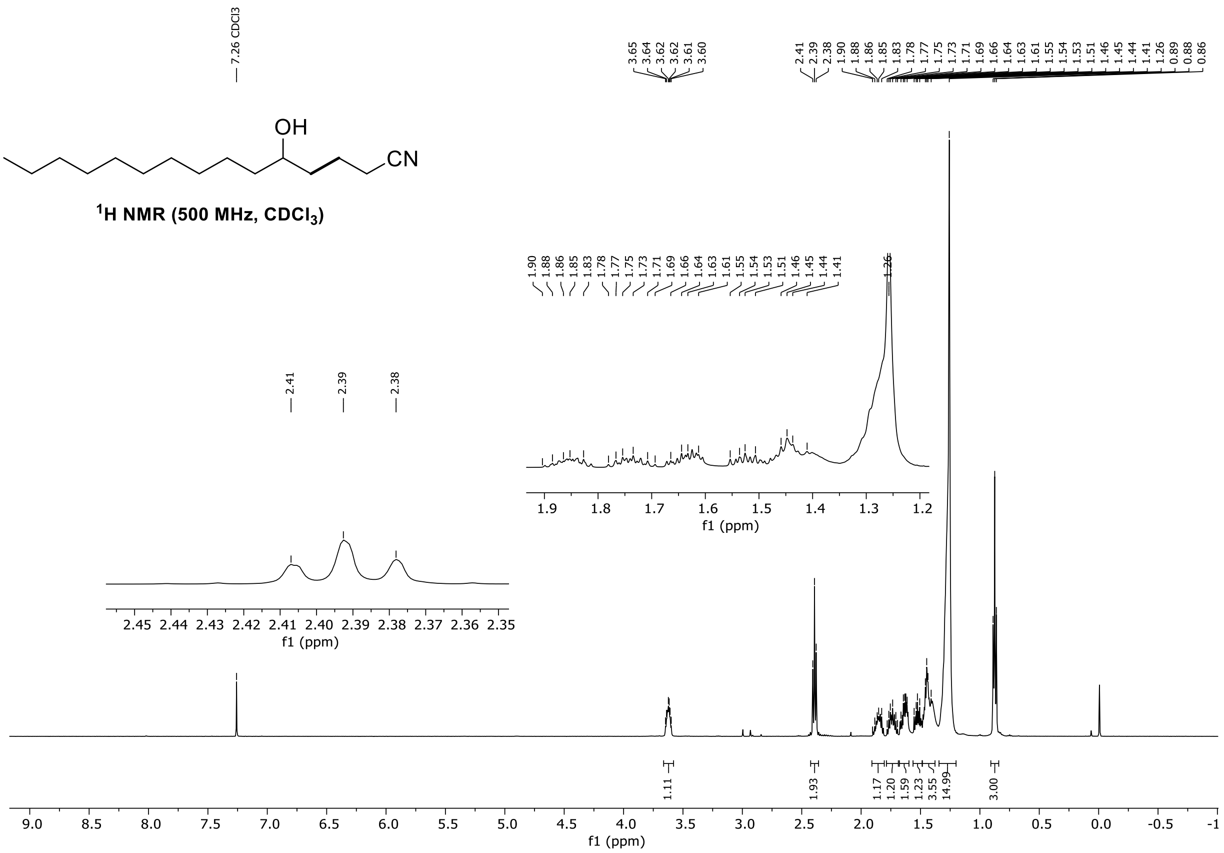

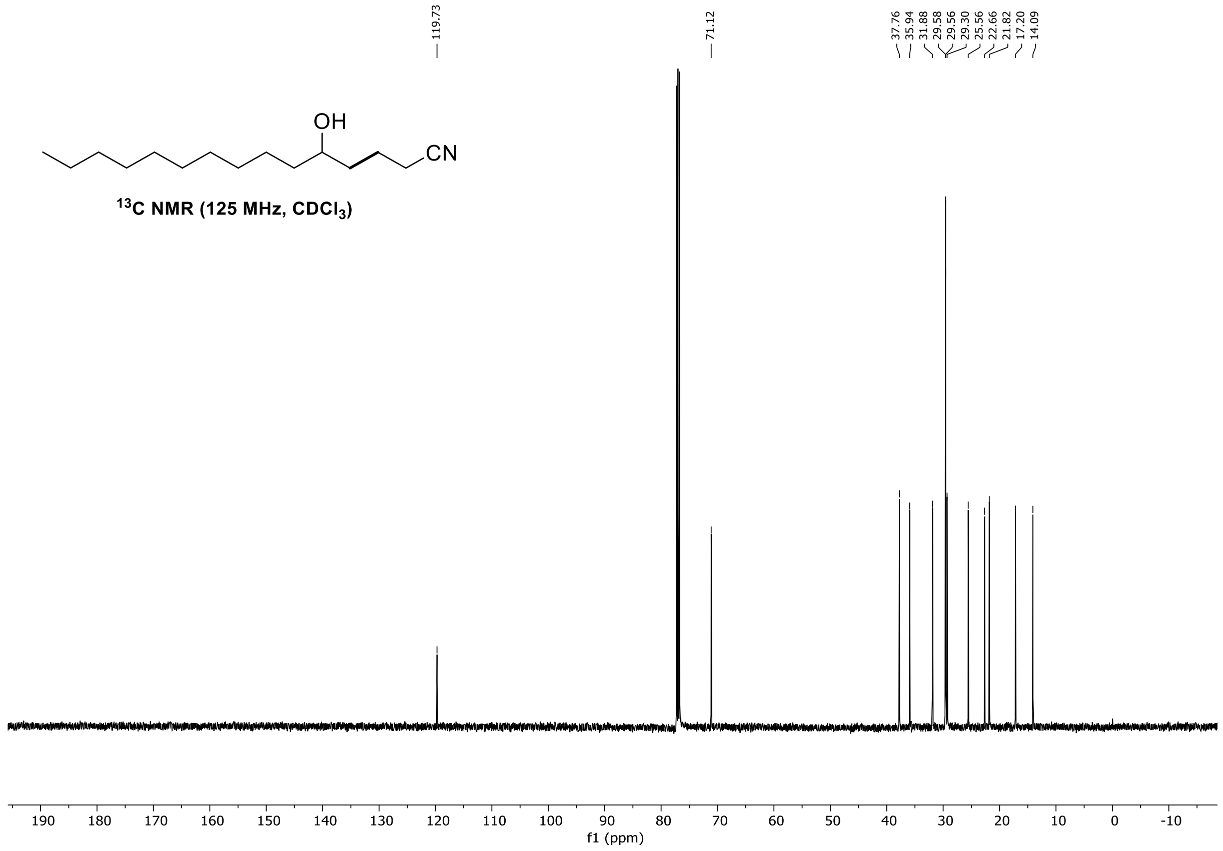

5-hydroxy-6-(naphthalen-2-yloxy)hexanenitrile (**11**)

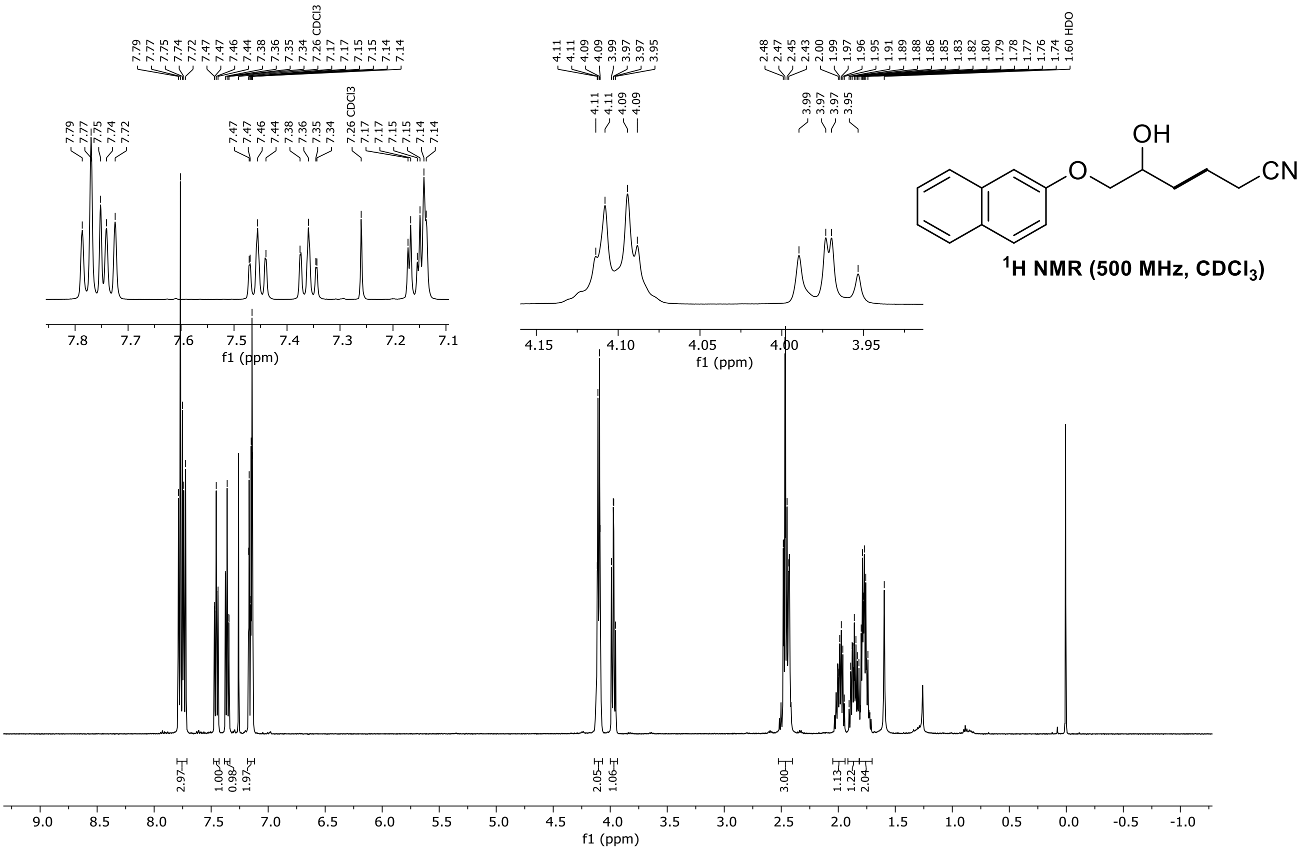

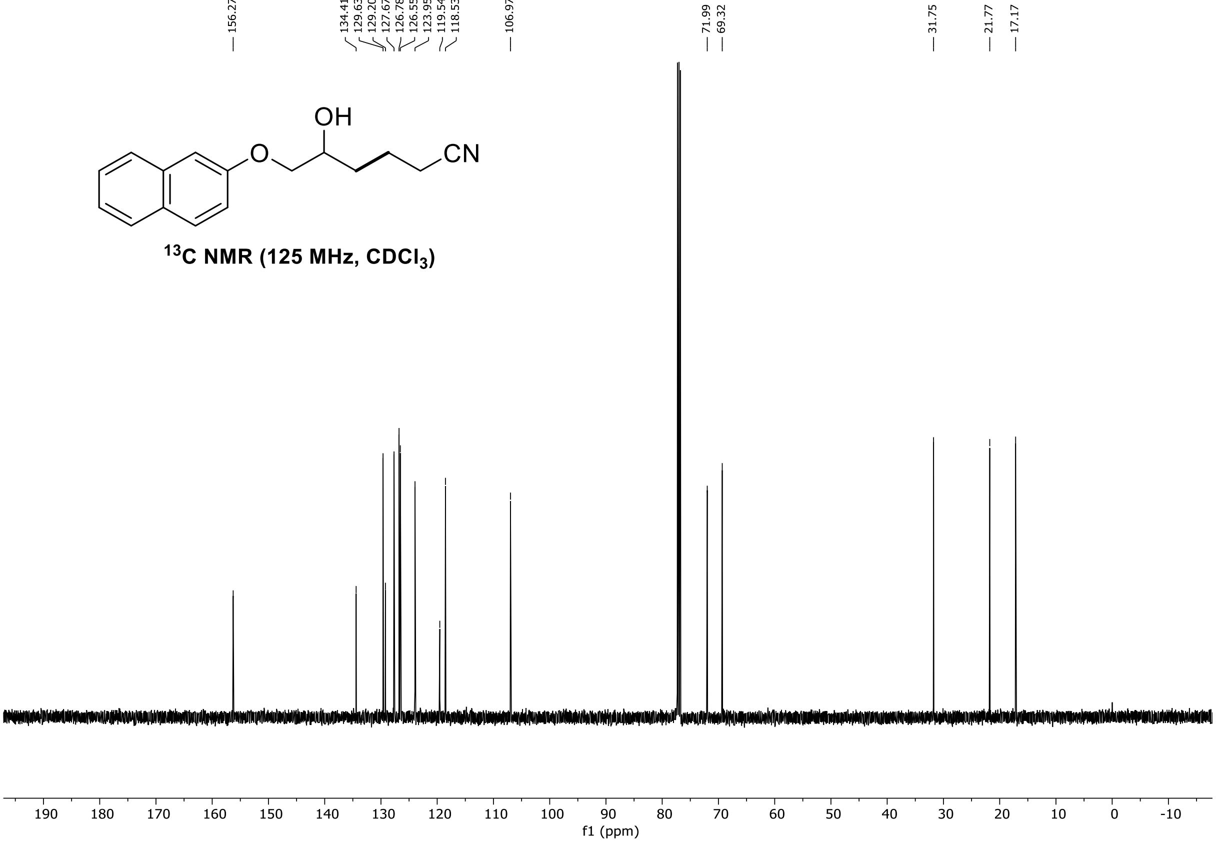

tert-butyl ((2S)-6-cyano-3-hydroxy-1-phenylhexan-2-yl)carbamate (**12**)

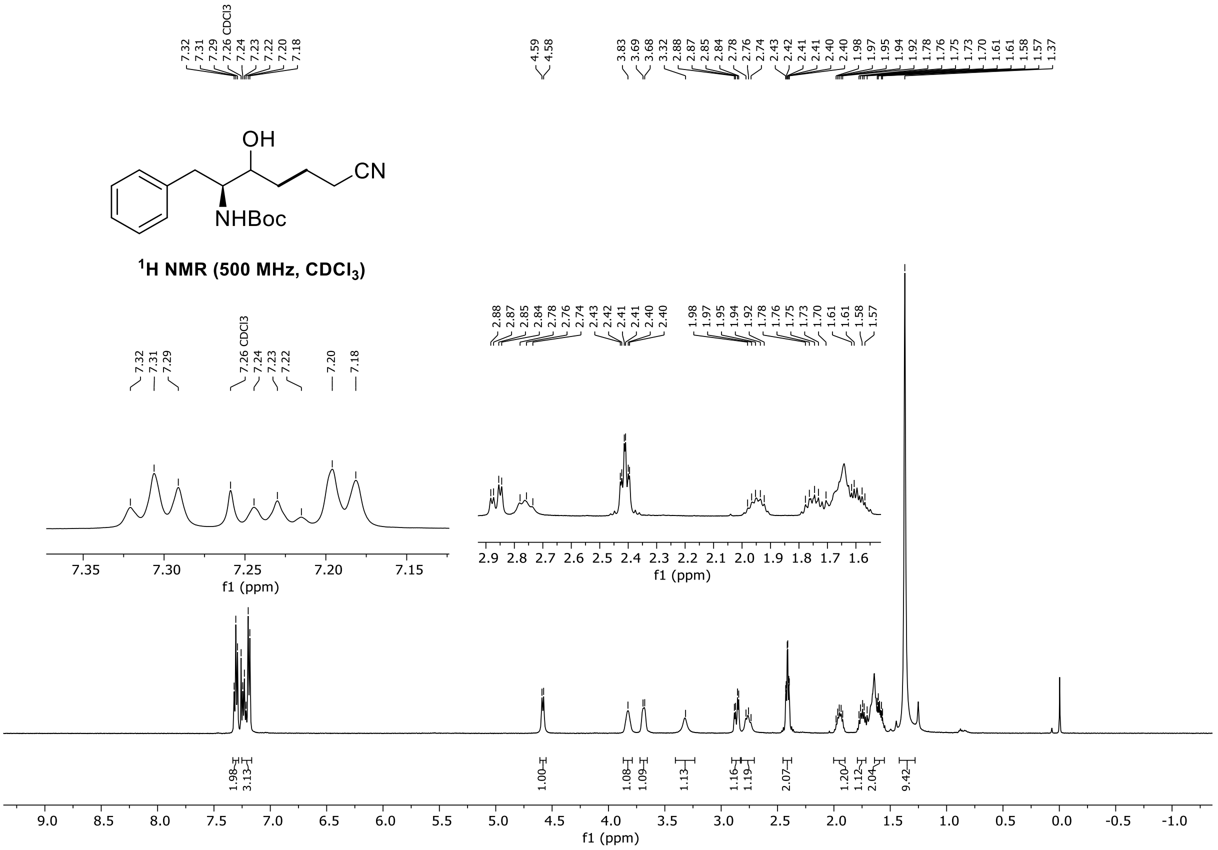

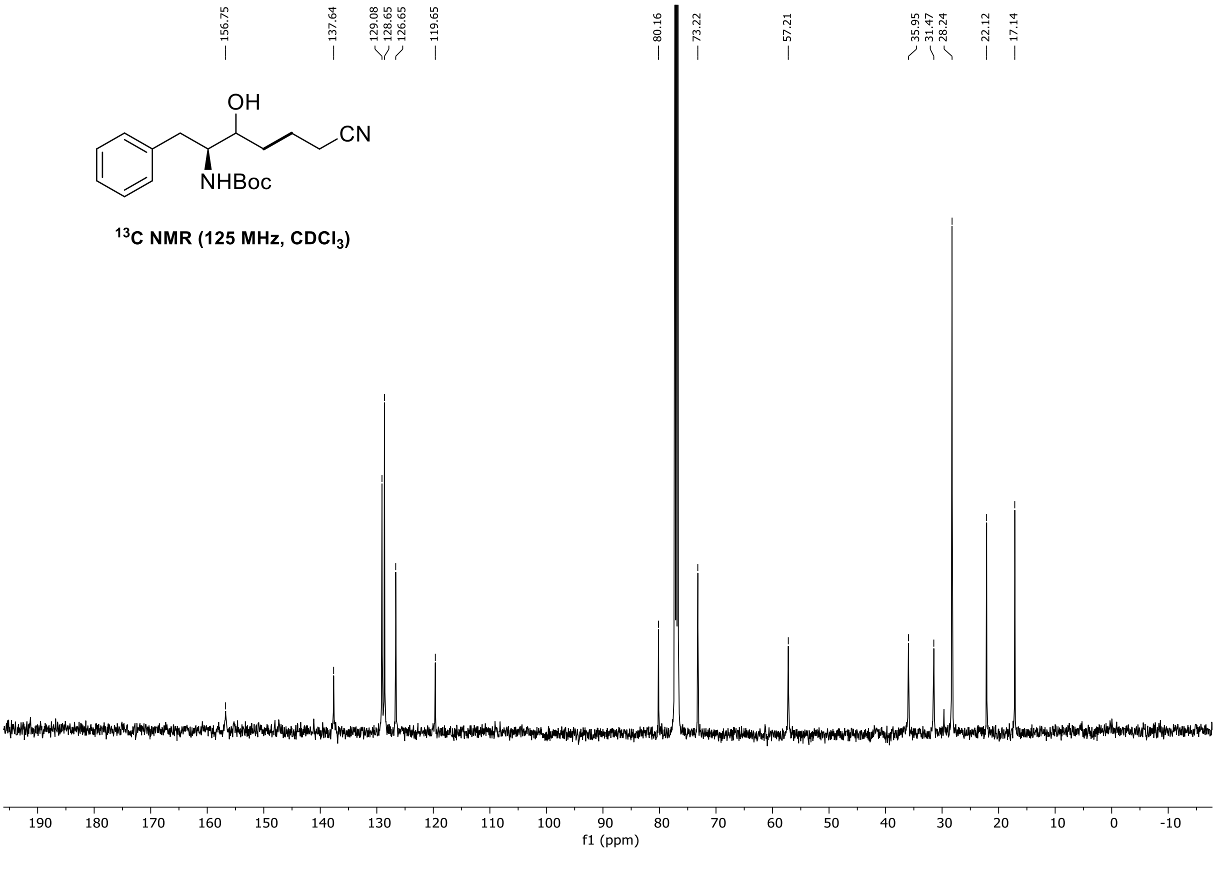

benzyl (5-cyano-2-hydroxypentyl)carbamate (**13**)

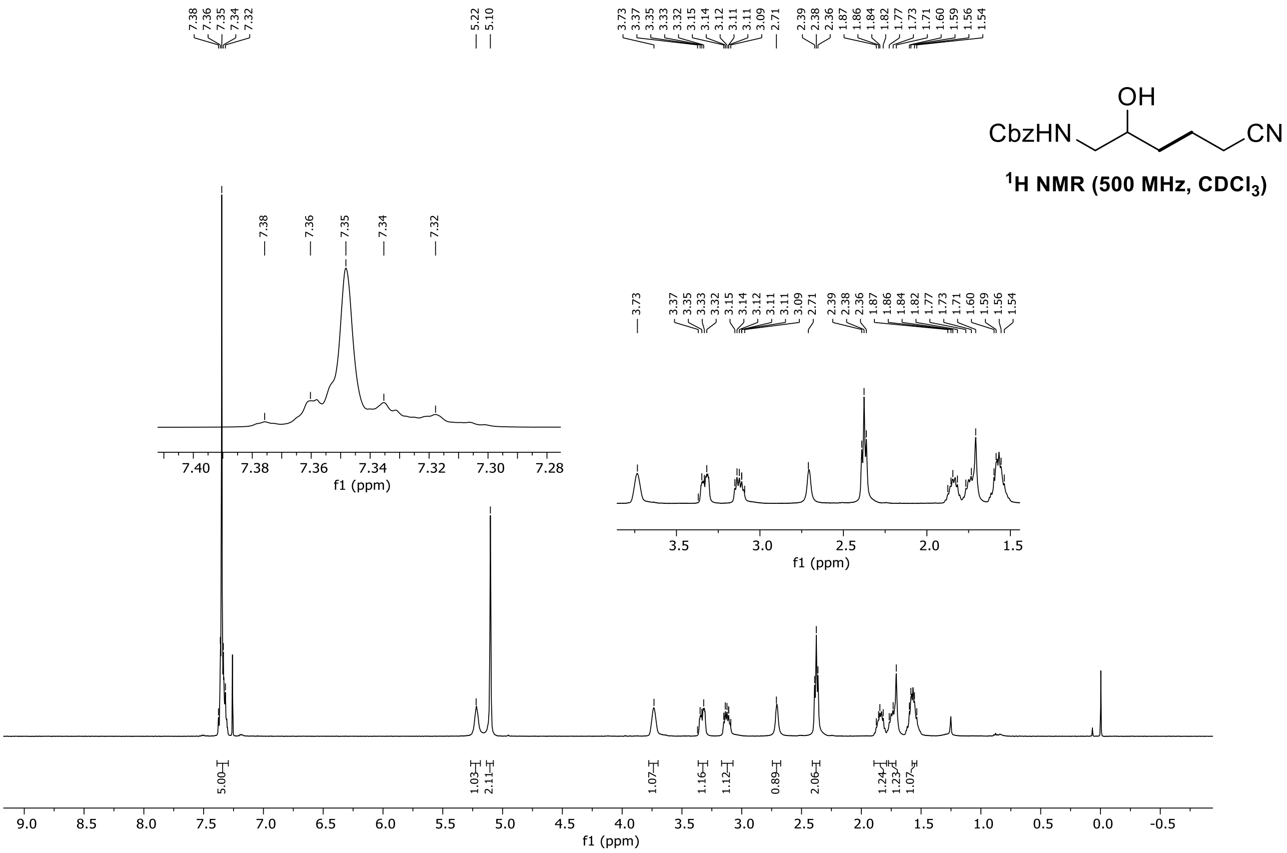

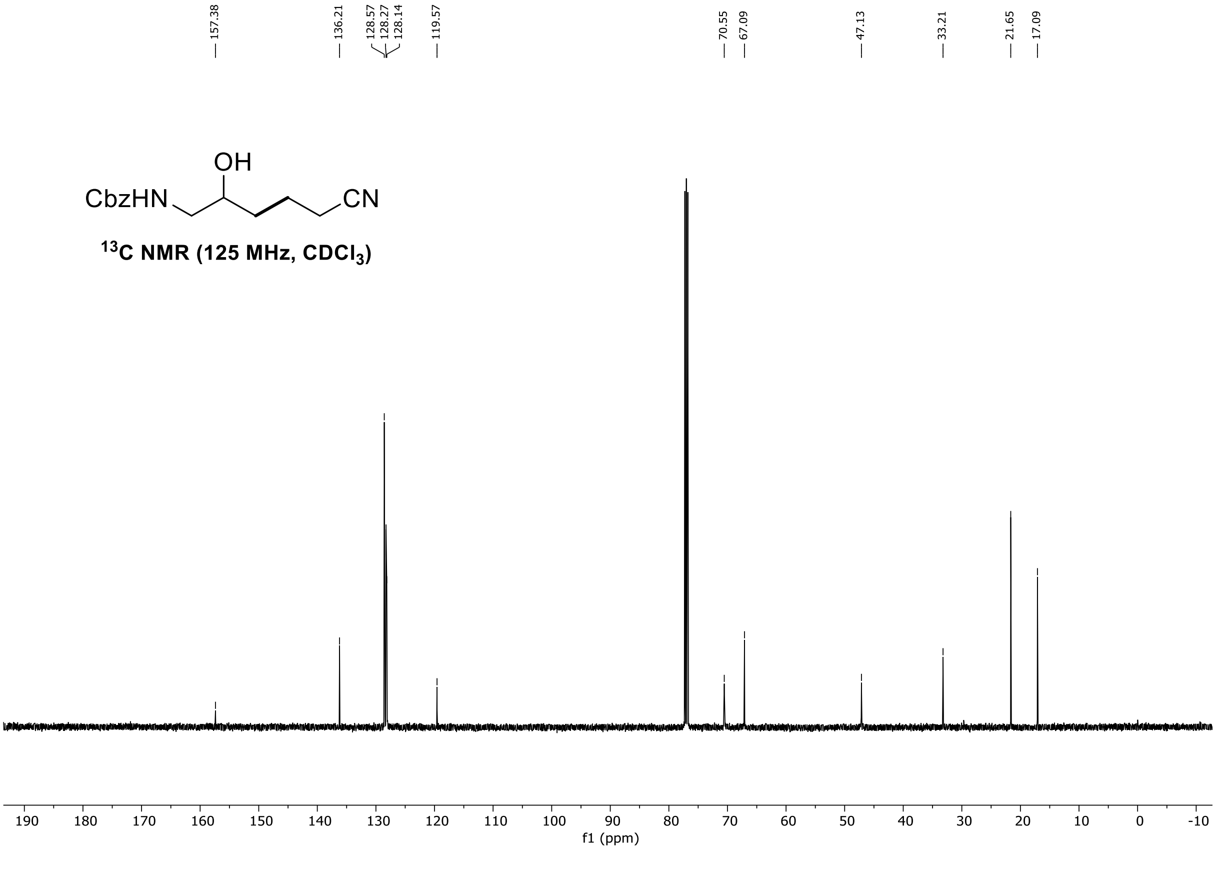

5-hydroxy-7-(phenylsulfonyl)heptanenitrile (**14**)

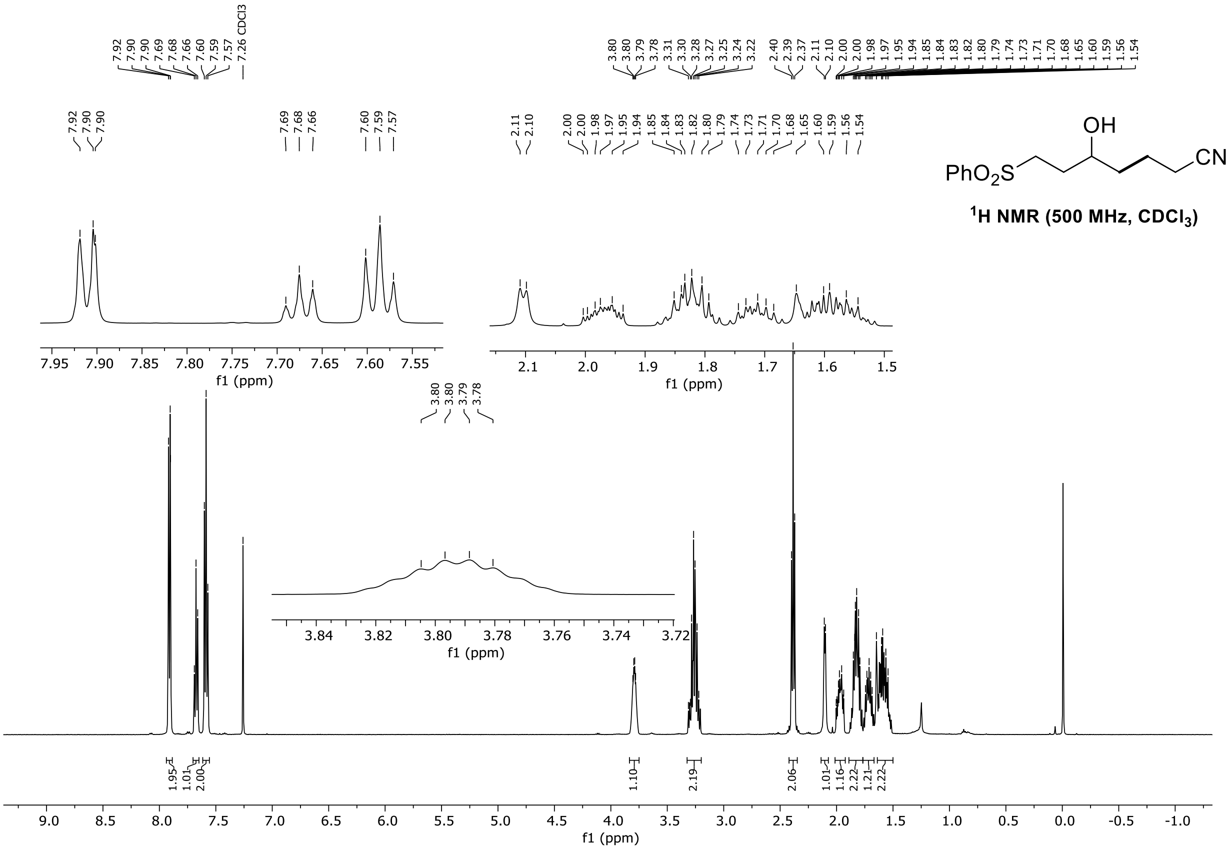

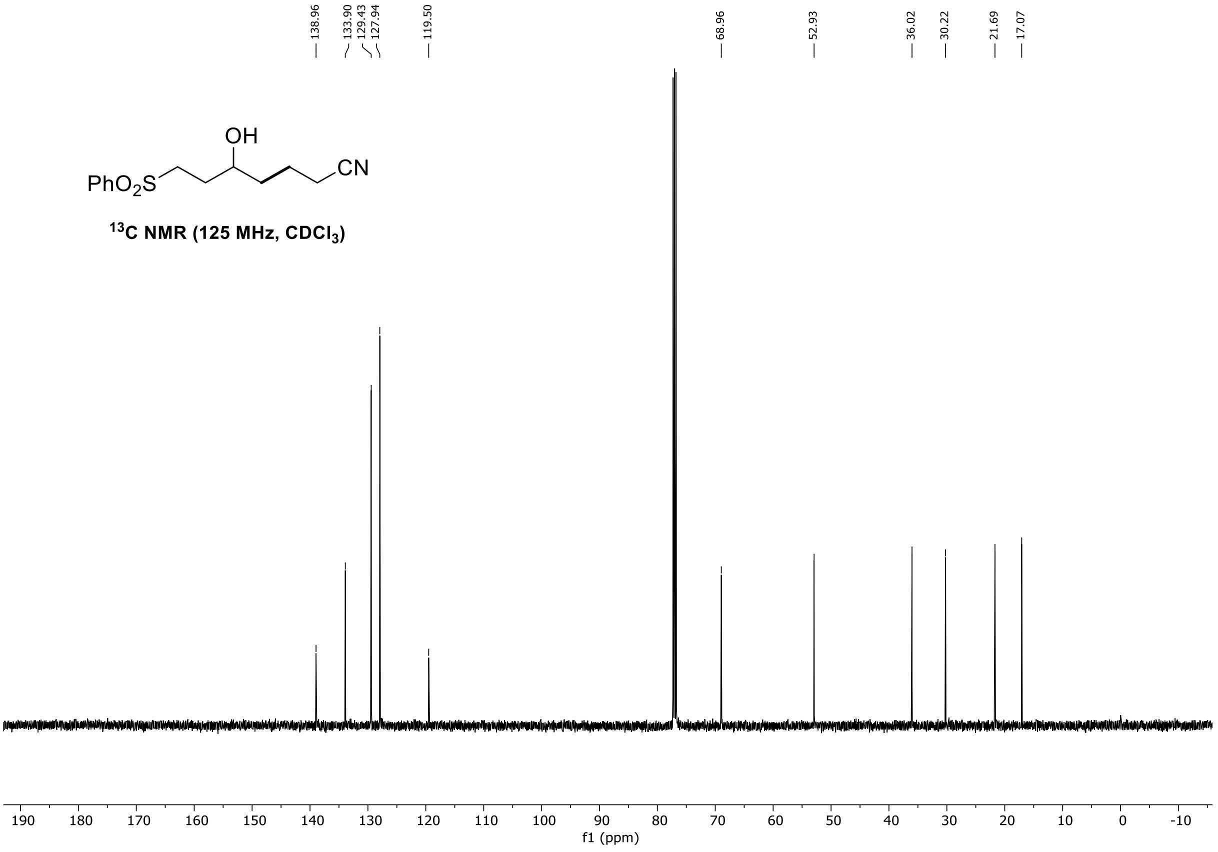

N-(1-cyanooctan-4-yl)-4-methylbenzenesulfonamide (**5**)

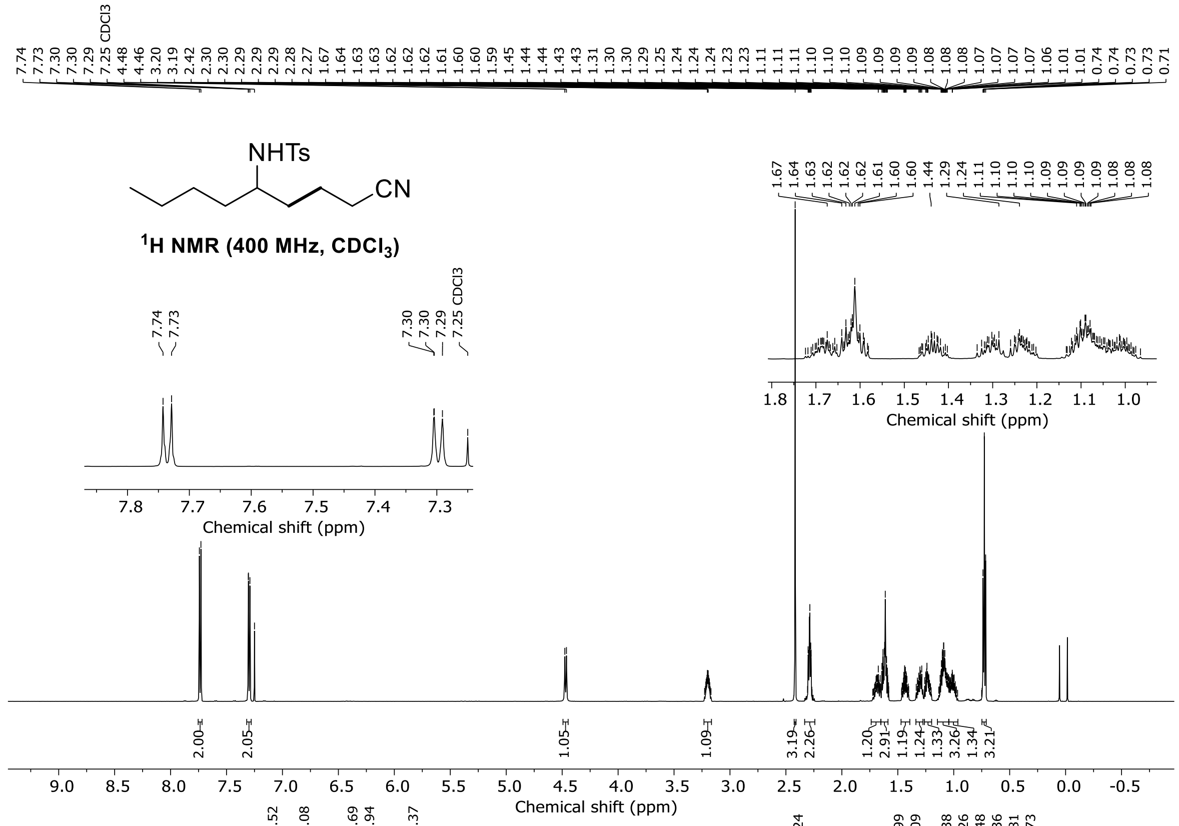

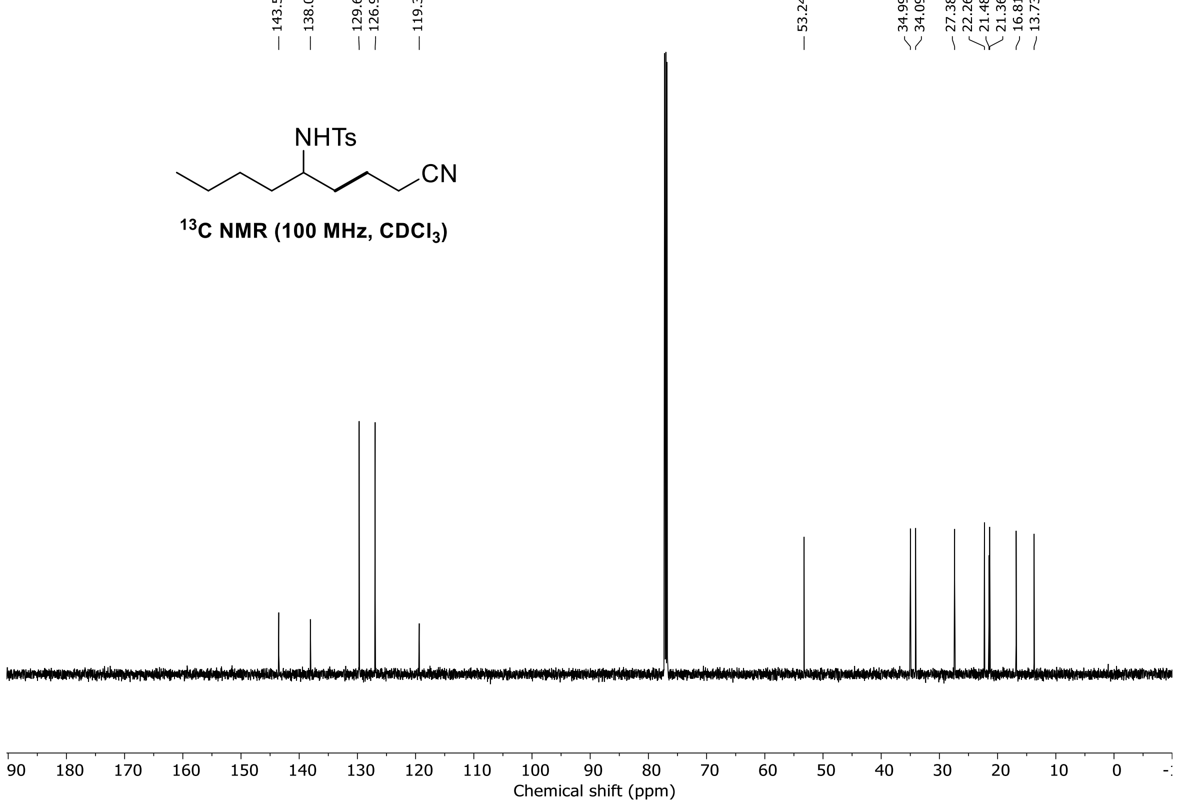

4-methyl-*N*-(9-oxodecan-5-yl)benzenesulfonamide (**15**)

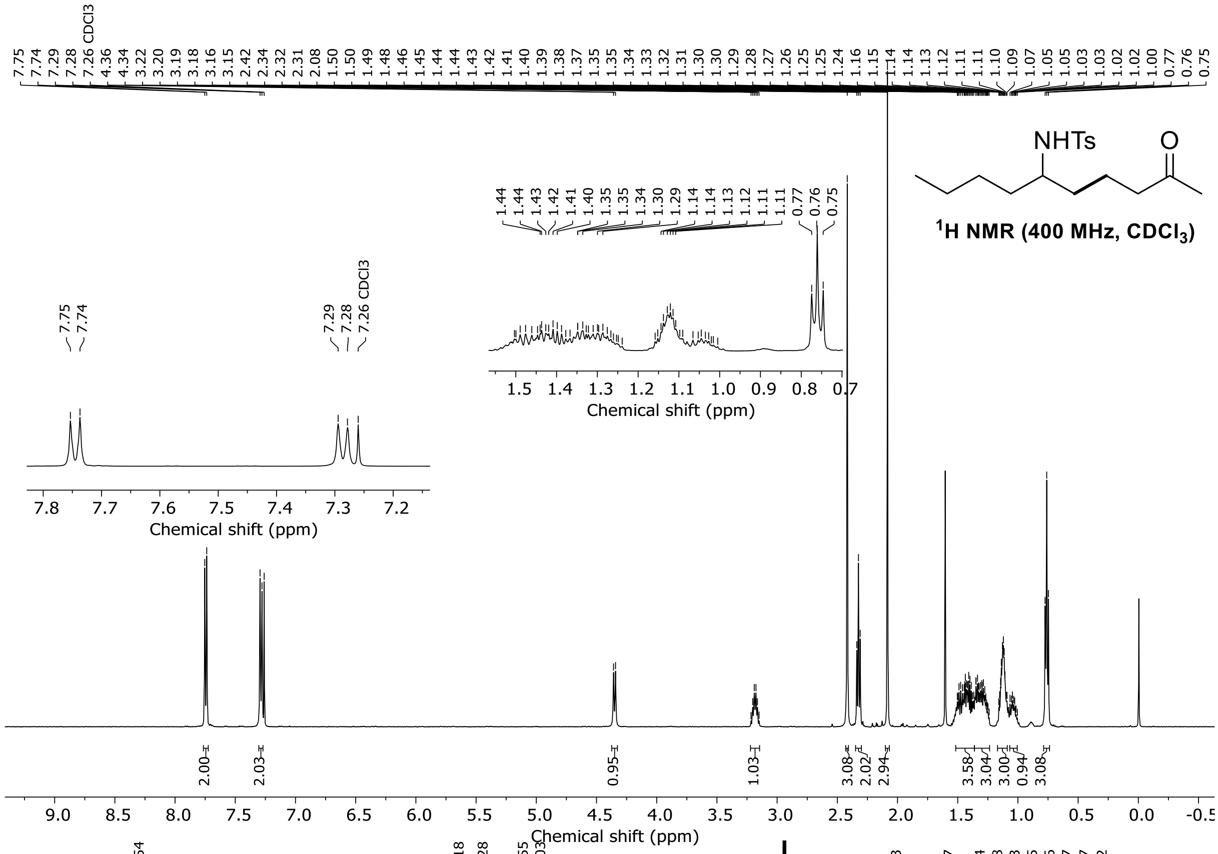

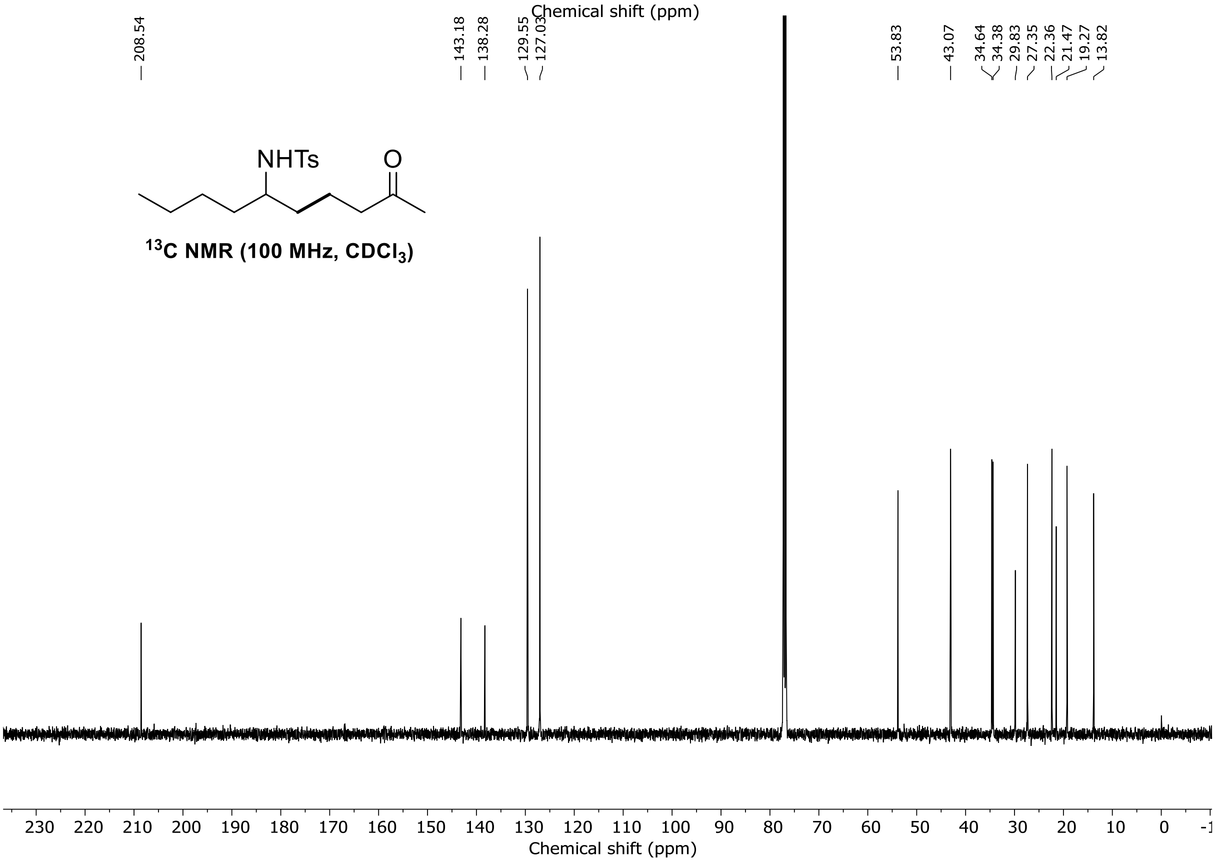

4-methyl-*N*-(1-(phenylsulfonyl)octan-4-yl)benzenesulfonamide (**16**)

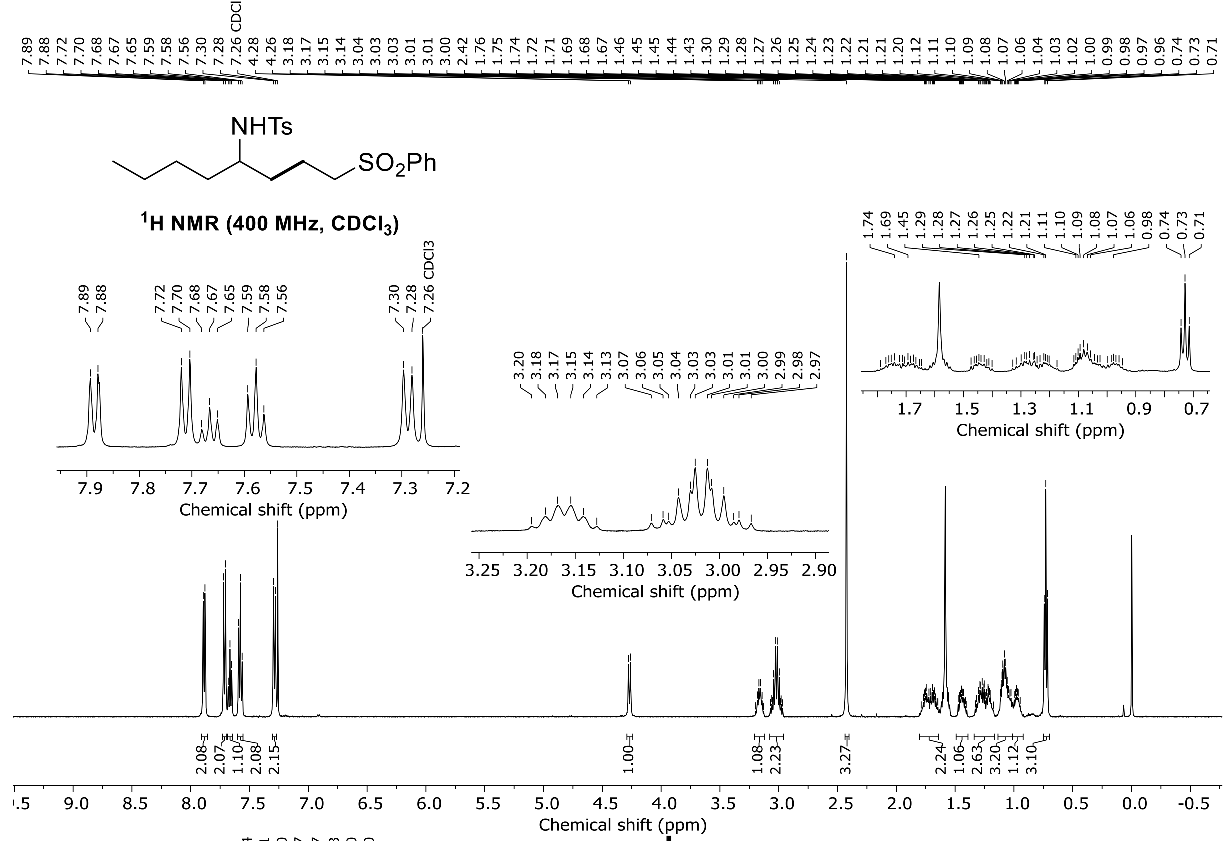

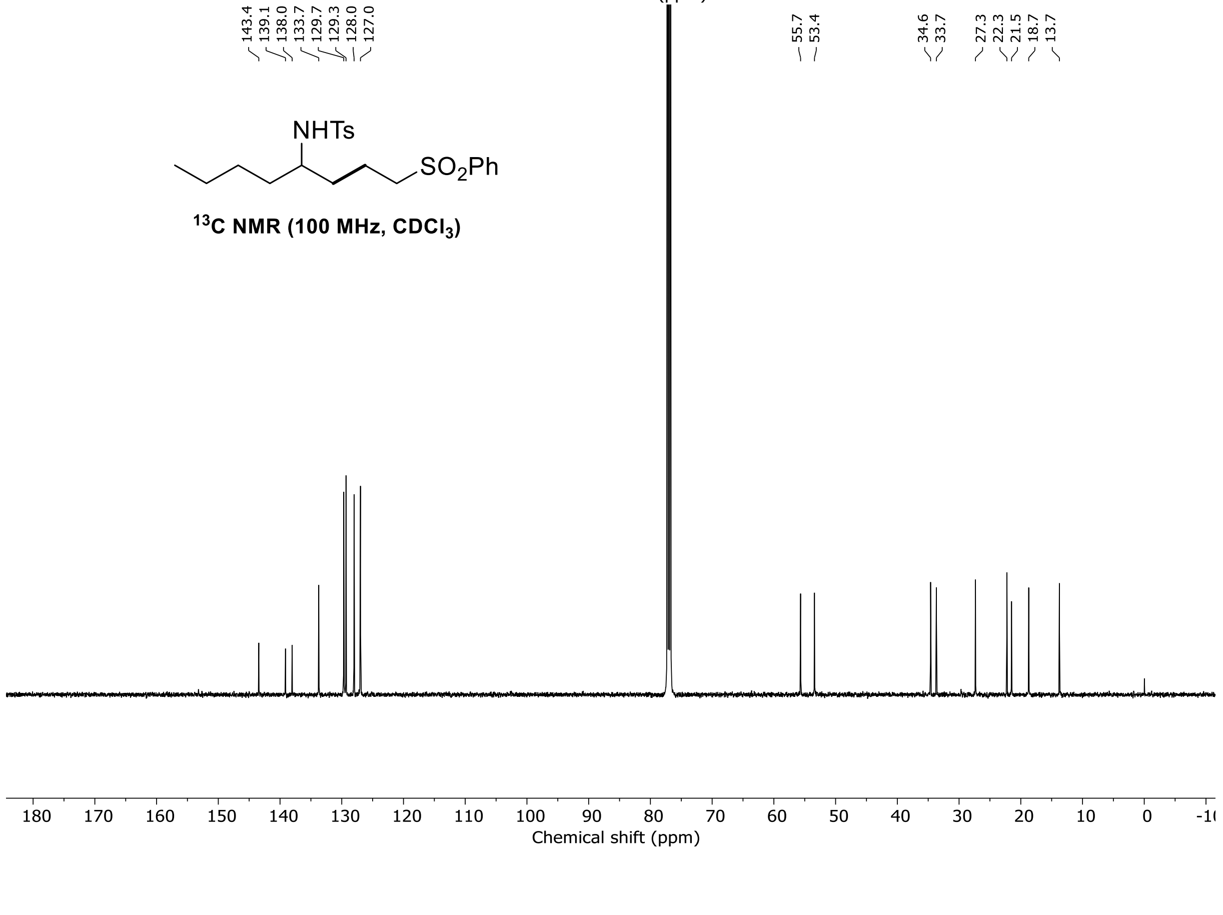

N-(1-cyanotetradecan-4-yl)-4-methylbenzenesulfonamide (**17**)

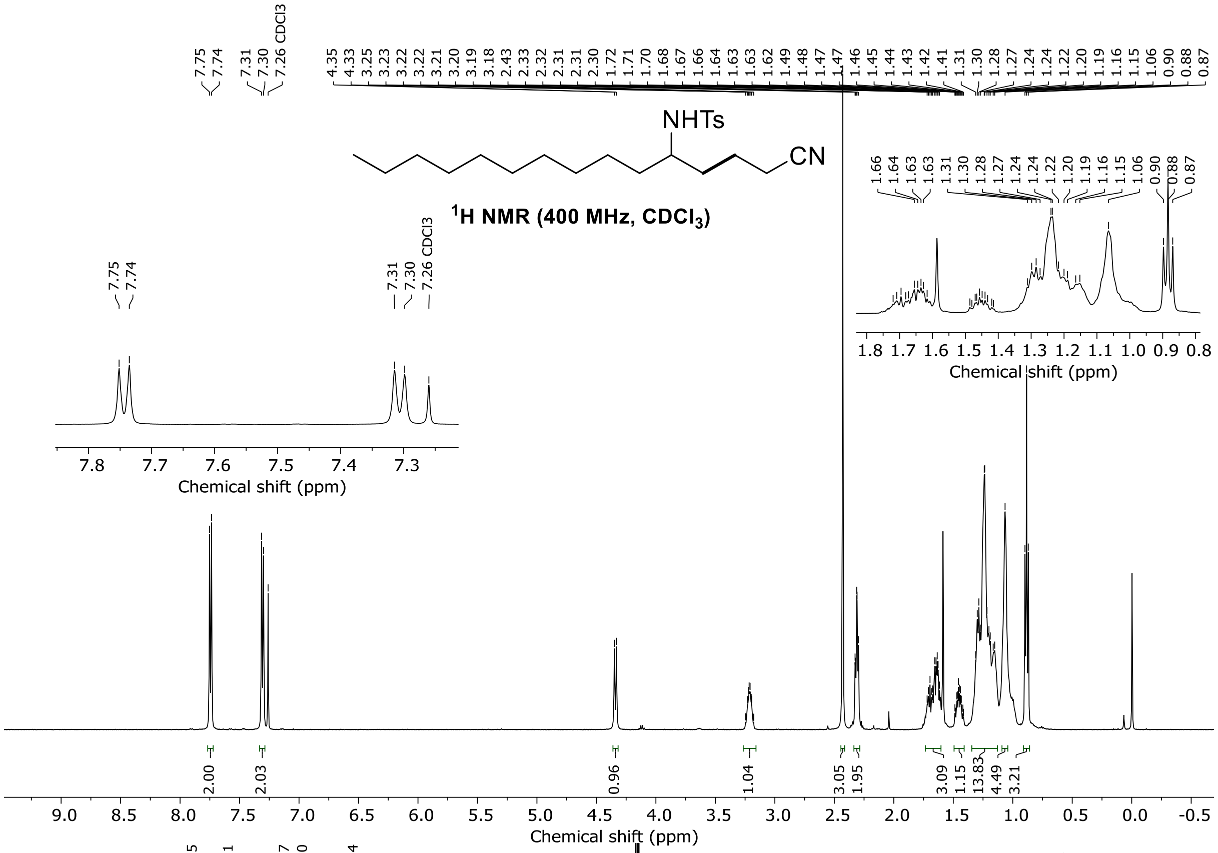

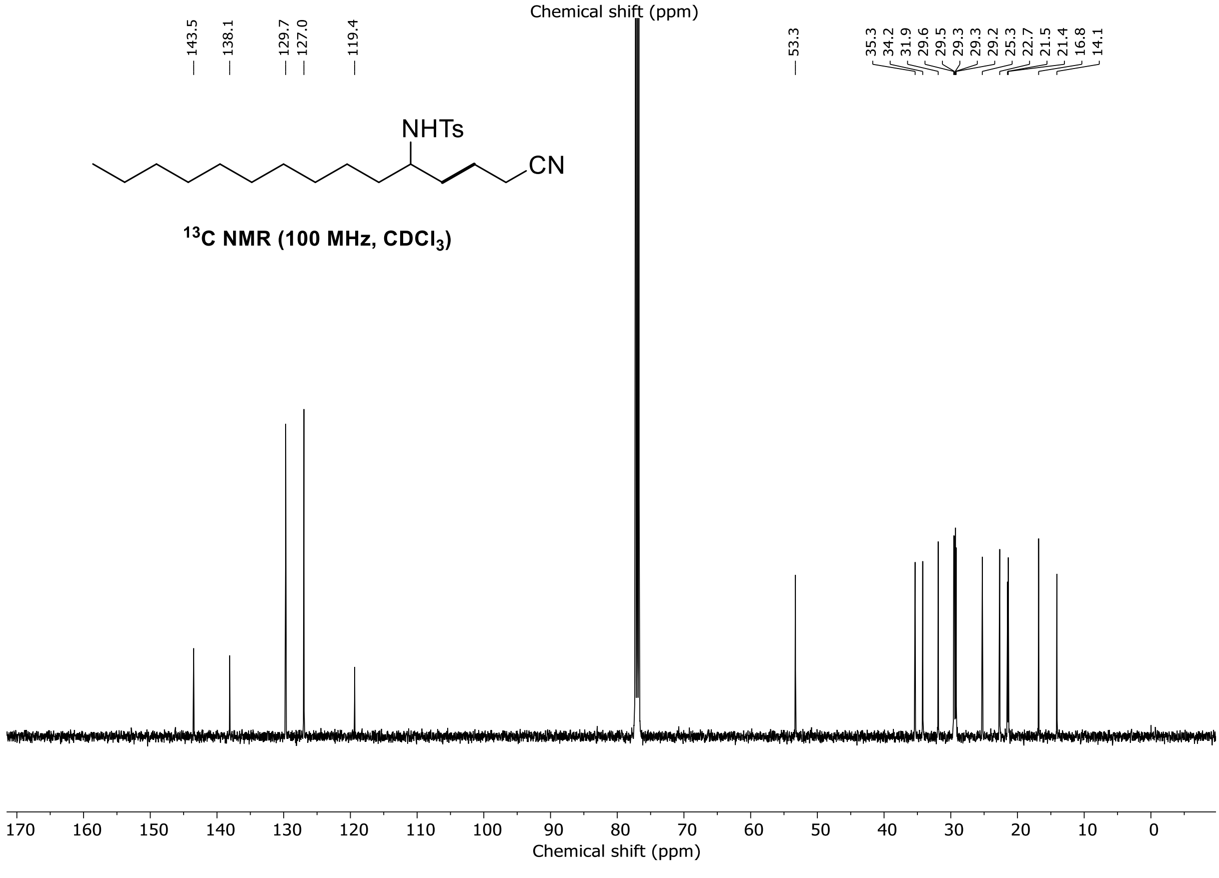

4-methyl-*N*-(2-oxohexadecan-6-yl)benzenesulfonamide (**18**)

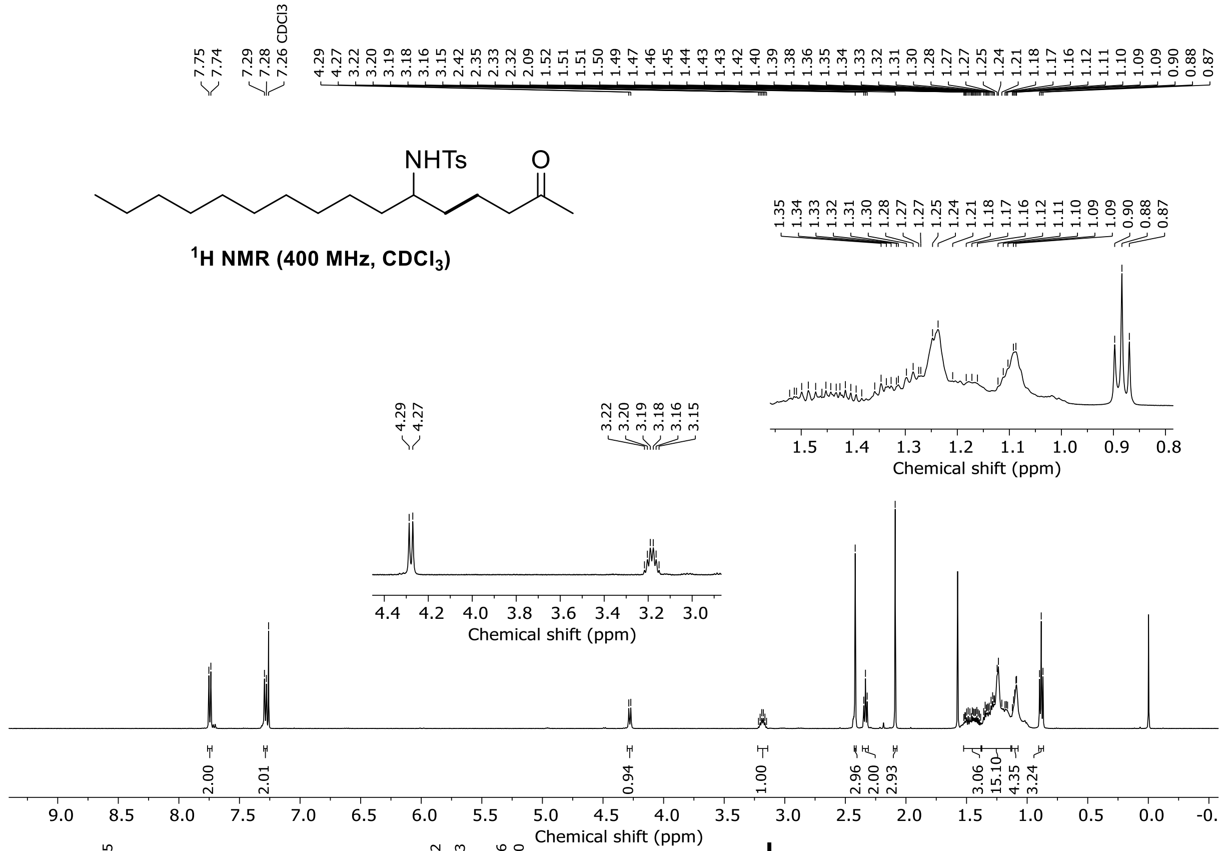

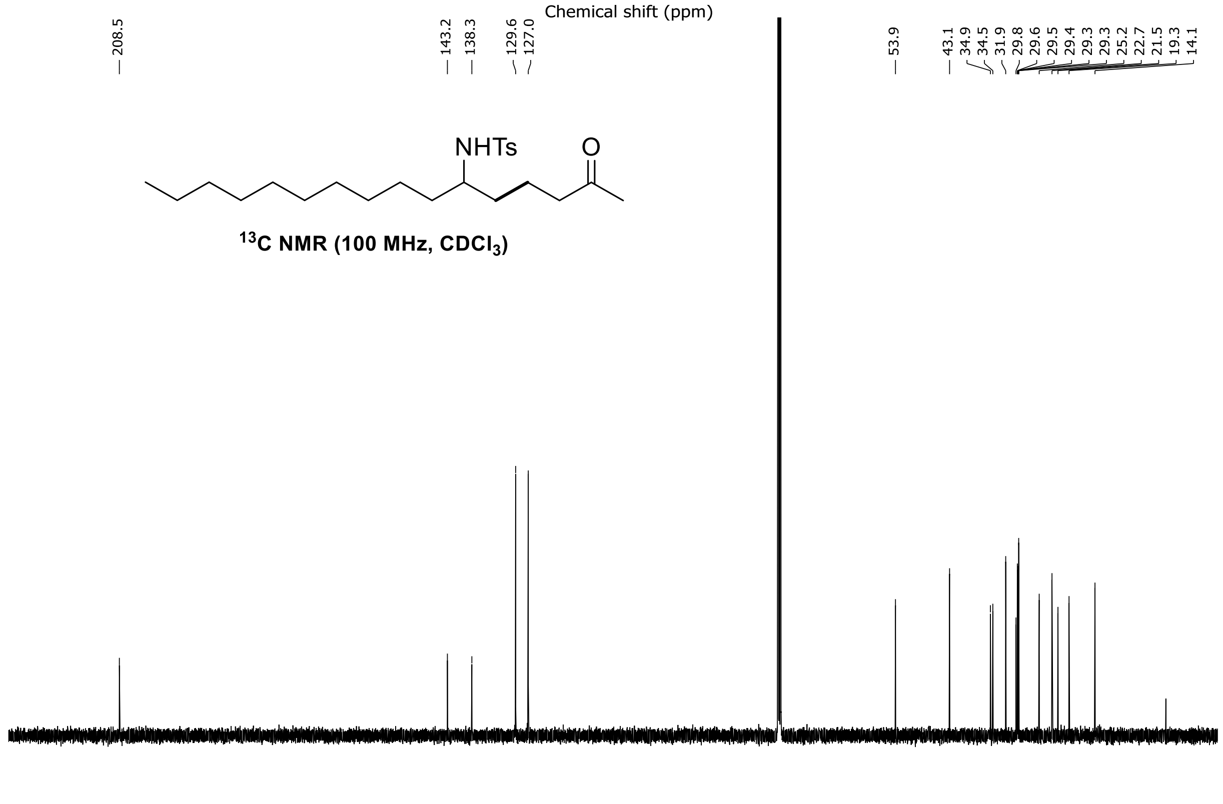

N-(cyclopent-2-en-1-yl)-4-methylbenzenesulfonamide (**19**)

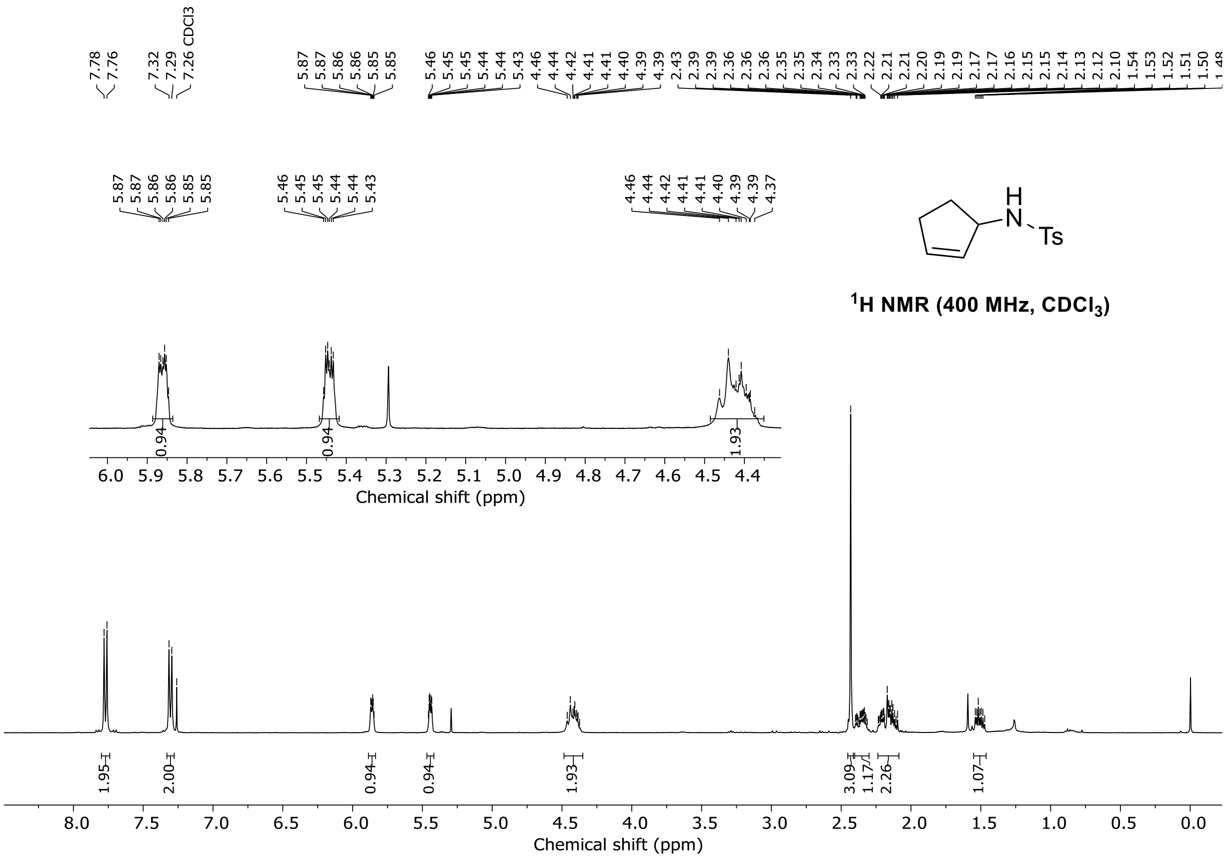

N-(cyclohex-2-en-1-yl)-4-methylbenzenesulfonamide (**20**)

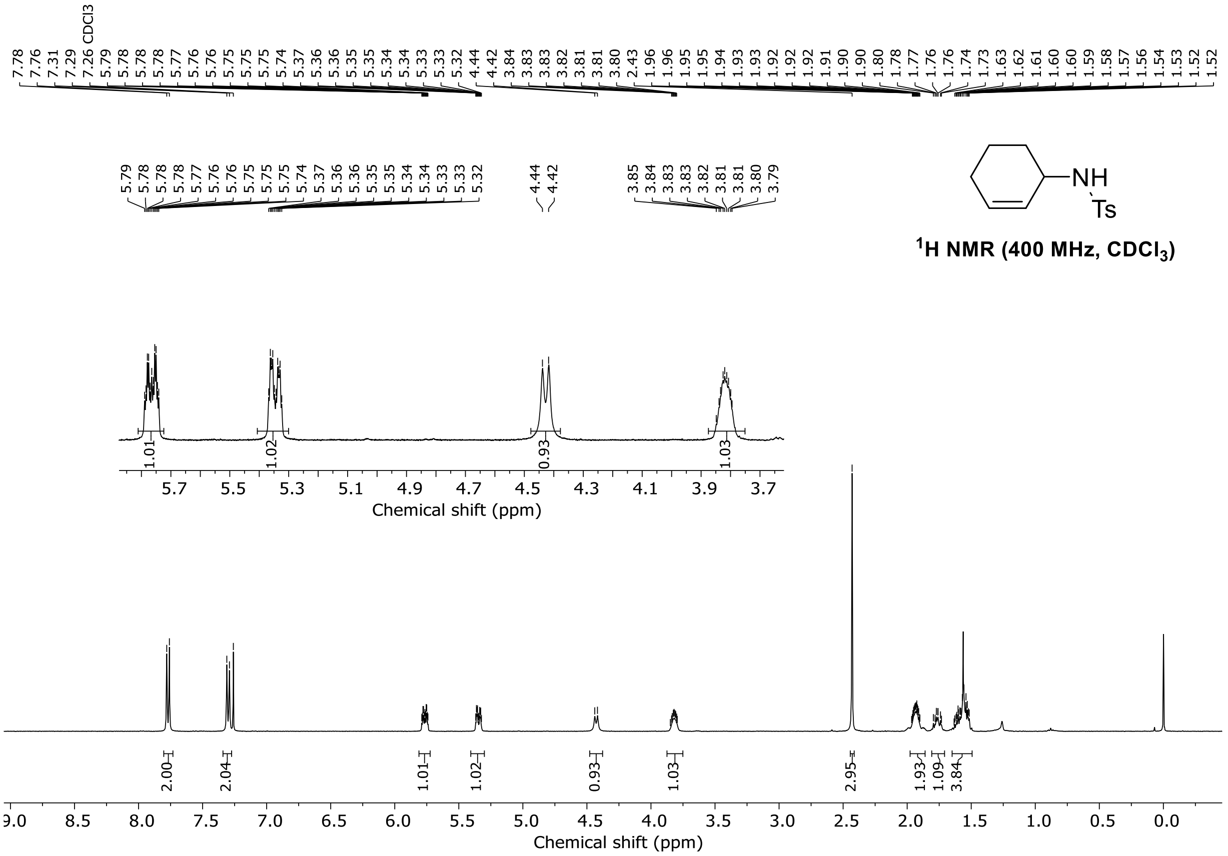

Supplement: Supplementary file 1 [file ol5c01376_si_001.pdf]
